# AutoEpiCollect, a Novel Machine Learning-Based GUI Software for Vaccine Design: Application to Pan-Cancer Vaccine Design Targeting PIK3CA Neoantigens

**DOI:** 10.3390/bioengineering11040322

**Published:** 2024-03-27

**Authors:** Madhav Samudrala, Sindhusri Dhaveji, Kush Savsani, Sivanesan Dakshanamurthy

**Affiliations:** 1 College of Arts and Sciences, The University of Virginia, Charlottesville, VA 22903, USA; 2 College of Science, Virginia Tech, Blacksburg, VA 24061, USA; 3 College of Humanities and Sciences, Virginia Commonwealth University, Richmond, VA 22043, USA; 4 Department of Oncology, Lombardi Comprehensive Cancer Center, Georgetown University Medical Center, Washington, DC 20007, USA

**Keywords:** epitope-based cancer vaccines, new GUI software, cancer vaccine design

## Abstract

Previous epitope-based cancer vaccines have focused on analyzing a limited number of mutated epitopes and clinical variables preliminarily to experimental trials. As a result, relatively few positive clinical outcomes have been observed in epitope-based cancer vaccines. Further efforts are required to diversify the selection of mutated epitopes tailored to cancers with different genetic signatures. To address this, we developed the first version of AutoEpiCollect, a user-friendly GUI software, capable of generating safe and immunogenic epitopes from missense mutations in any oncogene of interest. This software incorporates a novel, machine learning-driven epitope ranking method, leveraging a probabilistic logistic regression model that is trained on experimental T-cell assay data. Users can freely download AutoEpiCollectGUI with its user guide for installing and running the software on GitHub. We used AutoEpiCollect to design a pan-cancer vaccine targeting missense mutations found in the proto-oncogene PIK3CA, which encodes the p110ɑ catalytic subunit of the PI3K kinase protein. We selected PIK3CA as our gene target due to its widespread prevalence as an oncokinase across various cancer types and its lack of presence as a gene target in clinical trials. After entering 49 distinct point mutations into AutoEpiCollect, we acquired 361 MHC Class I epitope/HLA pairs and 219 MHC Class II epitope/HLA pairs. From the 49 input point mutations, we identified MHC Class I epitopes targeting 34 of these mutations and MHC Class II epitopes targeting 11 mutations. Furthermore, to assess the potential impact of our pan-cancer vaccine, we employed PCOptim and PCOptim-CD to streamline our epitope list and attain optimized vaccine population coverage. We achieved a world population coverage of 98.09% for MHC Class I data and 81.81% for MHC Class II data. We used three of our predicted immunogenic epitopes to further construct 3D models of peptide-HLA and peptide-HLA-TCR complexes to analyze the epitope binding potential and TCR interactions. Future studies could aim to validate AutoEpiCollect’s vaccine design in murine models affected by PIK3CA-mutated or other mutated tumor cells located in various tissue types. AutoEpiCollect streamlines the preclinical vaccine development process, saving time for thorough testing of vaccinations in experimental trials.

## 1. Introduction

In silico vaccine designs provide a cost-effective method for identifying potential immunogenic epitopes prior to clinical testing. The development of these vaccine designs has been steadily increasing due to the ability of vaccines to safely boost the immune system’s innate defense mechanisms against a broad number of diseases and pathogens. Some of the latest findings involving in silico vaccine designs include potential methods of protection against different viruses, bacteria, and some single-celled organisms [1,2,3,4,5]. However, there is a lack of powerful in silico epitope vaccine designs specifically targeting cancer. Furthermore, there is a demand for user-friendly graphical software to encourage clinical researchers in adopting in silico methods for cancer vaccine clinical trials. Some of our current methods, alongside other recent studies, manually filter a large set of epitope data using a set of strict exclusion criteria [6,7,8,9,10]. While these cancer vaccine designs identify immunogenic epitopes with favorable characteristics, much time is spent on data collection and epitope sorting. This limits the time available for further epitope analysis or preclinical testing. In one of our previous studies, we aimed to combat this issue by integrating multiple deep learning tools into a machine learning-based multivalent vaccine design called IntegralVac [11]. This comprehensive method reduces the time spent on data collection and drastically improves immunogenicity and binding affinity predictions [11]. In contrast, a study by Stranzl et al. focused on a different approach to MHC Class I epitope prediction. They introduced a ranking model named NetCTL, which is trained on known Class I ligands [12]. NetCTL ranks epitopes based on proteasomal cleavage and other epitope characteristics, offering a more efficient way to prioritize epitopes for further analysis. However, this model is limited in the number of characteristics it considers and cannot be used to predict MHC Class II epitopes.

We developed AutoEpiCollect, an automated epitope selection software that integrates a novel machine learning-based epitope ranking model with individual variable filtration. AutoEpiCollect features a user-friendly graphical user interface (GUI) designed to identify potential MHC Class I and II T-cell eliciting epitopes, specifically tailored for peptide-based cancer vaccines. The software requires only two inputs: the name of the target oncogene and a list of point mutations associated with prevalent cancer subtypes affected by the mutated gene. AutoEpiCollect then gathers mutated epitope characteristics and employs a machine learning model to rank epitopes based on their immunogenic potential. To ensure the selection of safe and stable epitopes, we incorporated individual variable filtration using exclusion criteria. Additionally, we used our PCOptim and PCOptim-CD programs to optimize the final list of epitopes before evaluating the population coverage of a vaccine containing the predicted top epitopes [6,7]. In this study, we applied AutoEpiCollect’s vaccine design process to develop a pan-cancer epitope-based vaccine targeting PIK3CA-mutated cancers.

Pan-cancer epitope vaccines target epitopes from multiple cancer types. Epitopes taken from tumor-specific antigens (TSAs) and tumor-associated antigens (TAAs) are delivered via vaccination, where antigen-presenting cells (APCs) uptake them. The epitopes are presented on major histocompatibility complex molecules as markers for T-cells to activate. As a result, cytokines are released and regulate immune responses towards the epitopes [13]. While a pan-cancer epitope vaccine targeting various PIK3CA gene mutations has not been developed yet, an in vitro study focused on the H1047R point mutation, using PBMCs from healthy individuals, induced T-cell responses [14]. Specifically, 27mers peptides from the PIK3CA H1047R point mutation stimulated CD4^+^ and CD8^+^ T-cell responses in 16% and 4.0% of donors, despite the study’s limited sample size. 

However, this study focused on a few epitopes due to the extended duration required to obtain epitopes from an extensive set of point mutations in the PI3KCA gene. Pan-cancer vaccines target a greater number of mutations, thereby increasing the number of epitopes involved. Furthermore, they facilitate safer, more precise immune responses against mutated epitopes. Cafri et al. designed an mRNA cancer vaccine containing mutated sequences from TP53, KRAS, and PIK3CA genes, among others [15]. This vaccine was capable of eliciting T-cell responses against selected neoantigens in metastatic gastrointestinal cancer patients. No significant clinical responses were observed in the patients treated with the vaccine during the trial; however, Cafri et al. were able to identify T-cell responses against in silico predicted neoantigens [15]. This study highlights the immunogenic potential of a cancer vaccine consisting of virtually predicted epitopes targeting missense mutations in PIK3CA. Based on the studies discussed above, we decided to research common PIK3CA point mutations in an effort to develop a pan-cancer vaccine with in silico tools [9,10].

Prevalent PIK3CA missense mutations found in colorectal adenocarcinoma (CRC), breast cancer (BC), endometrial carcinoma (EC), glioblastoma multiforme (GBM), and meningioma are primarily seen in exons 9 and 20 [16,17,18,19,20,21,22]. These mutations affect the helical and kinase domains of the p110ɑ protein, leading to increased cell proliferation and tumor metastasis [17]. Current treatment options aiming to prevent metastatic recurrence include endocrine therapy for HR+ tumors, aromatase inhibitors, tamoxifen (associated with a 50% reduced recurrence rate), and chemotherapy for the unfavorable prognosis of triple-negative BC [23]. Several small-molecule inhibitors targeting the PI3K protein have also demonstrated efficacy in inducing tumor regression in cells harboring PIK3CA mutations. In a study by Janku et al., the FDA-approved drug Alpelisib was used to target PIK3CA-mutated BC, resulting in improved clinical responses among patients receiving the inhibitors [24]. Despite these promising outcomes, small molecule inhibitors are characterized by their limited pathway targeting, potentially leading to restricted effectiveness. Furthermore, they may induce off-target cytotoxic effects, further complicating their use in clinical settings [25].

Due to the limited success of PIK3CA-directed treatments, patients with the cancers discussed above could potentially experience improved clinical outcomes through a pan-cancer epitope vaccine targeting prevalent PIK3CA point mutations. Our study considered various clinical variables based on prior studies that served as primary benchmarks when developing our novel epitope selection process [6,7,26,27]. These variables confirmed that AutoEpiCollect’s top predicted epitopes were immunogenic, safe, and stable. Our automated AutoEpiCollect GUI software is the first machine learning-based epitope selection method to target PIK3CA missense mutations. With its ability to streamline a significant portion of the preclinical vaccine development process, AutoEpiCollect has the potential to reduce days of preclinical epitope selection procedures to just a few hours.

## 2. Materials and Methods

### 2.1. MHC Class I and II Epitopes and Variable Collection Using AutoEpiCollect

AutoEpiCollect is a graphical user interface (GUI) software designed to automatically gather information on mutated epitopes, ultimately creating an extensive list of potential immunogenic epitopes for a pan-cancer epitope vaccine. AutoEpiCollect utilizes a combination of online and downloadable in silico tools, the IEDB-API, and our novel probabilistic logistic regression model scoring function to collect, rank, and filter epitope data. The GUI version of AutoEpiCollect is implemented in Python using PyQt5 and spans approximately 1800 lines of Python code. For detailed instructions on installation and how to run the software, refer to the documentation website found in the link provided under the ‘About this project’ section on https://github.com/mvsamudrala/AutoEpiCollect (accessed on 2 February 2024).

AutoEpiCollect obtains wild-type genes from UniProt and generates FASTA-formatted mutant sequences [28] to initiate the vaccine design process. It then inputs the mutant sequences into the IEDB-API and receives binding affinities of 9 and 10mers epitopes to the 27-allele HLA Class I reference set, as well as binding affinities of 15mers epitopes to the 27-allele HLA Class II reference set [29]. The 27-allele HLA Class I and II reference sets are located in Table 1. The binding predictions are performed using NetMHCpan-4.1 and NetMHCIIpan-4.1, which are neural networks trained on large datasets of MHC Class I and II epitope binding affinity data [30]. AutoEpiCollect automatically filters out unmutated epitopes from the dataset.

The remaining mutated epitopes and their corresponding binding affinities are categorized into four groups: “STRONG”, “NORMAL”, “WEAK”, or “N/A”, based on their binding affinity scores. Epitopes with scores less than or equal to 50 nM are labeled as “STRONG”, epitopes with scores ranging between 50 nM and 500 nM, inclusive of 500 nM, are classified as “NORMAL”, and epitopes with scores between 500 nM and 5000 nM, inclusive of 5000 nM, are considered “WEAK”. Any scores exceeding 5000 nM receive the “N/A” label. Table 2 shows the labeling scheme used for the epitope binding affinities. We established these designations after prior literature recommendations describing epitopes’ binding strength to MHC molecules based on their IC_50_ values [31].

**Figure 1:**
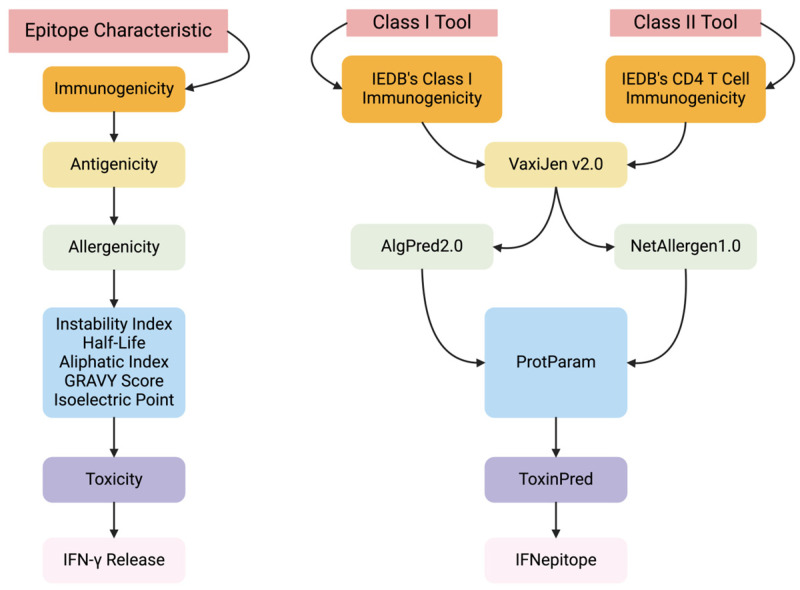
AutoEpiCollect follows a specific workflow of tools and collection steps to gather important epitope characteristics. To start, two distinct tools are used to collect immunogenicity data for MHC Class I and II epitopes, both sourced from the IEDB [32,33]. Subsequently, VaxiJen v2.0 is used to obtain antigenicity data for both MHC Class I and II epitopes [34]. This is followed by allergenicity predictions using AlgPred2.0 and NetAllergen1.0 for Class I and II epitopes, respectively [35,36]. After the compilation of these four epitope characteristics required for epitope ranking, AutoEpiCollect begins to collect additional characteristics for individual variable filtration. Instability indexes, half-lives, aliphatic indexes, GRAVY scores, and isoelectric points of MHC Class I and II epitopes are obtained using ProtParam on the ExPASy server [37]. The final in silico tools employed for gathering epitope characteristics are ToxinPred for epitope toxicity and IFNepitope for IFN-γ release data, both for Class I and II epitopes [38,39]. Additional information about each tool can be accessed by referring to the respective manuscripts cited.

After labeling binding affinity data, AutoEpiCollect employs a variety of in silico tools to gather epitope characteristic data for ranking. These specific MHC Class I and II epitope characteristics and their corresponding in silico tools are highlighted in Figure 1. 

### 2.2. Developing Scoring Functions to Rank MHC Class I and II Epitopes

Using the epitope characteristics discussed above, we initially aimed only to apply individual filters to construct a vaccine containing epitopes that met all the essential criteria. However, this process prematurely emphasized characteristics like allergenicity, resulting in the exclusion of nearly all epitopes with strong binding affinity and good immunogenic potential. We developed a machine learning algorithm to rank epitopes to address this issue and achieve a balanced selection of epitopes that can elicit a robust immunogenic response while maintaining normal physiological function. Since our model needed to work as a ranking algorithm, we evaluated the performances of two types of models: a linear regression model and a probabilistic logistic regression model. The symbolic equations representing these regression models, Equations (1)–(3), show how AutoEpiCollect uses the optimal weights assigned to each characteristic—binding affinity, immunogenicity, antigenicity, and allergenicity—and outputs a probability, indicating if a given epitope is likely to induce an immunogenic response.

(1) $$Y=\beta_{0}+\beta_{1}ɑ+\beta_{2}Ɣ+\beta_{3}\delta +\beta_{4}\varepsilon$$

In the linear regression model above, Y represents the probability of an epitope eliciting a positive T-cell response. ɑ, Ɣ, δ, and ε represent an epitope’s predicted immunogenicity, antigenicity, allergenicity, and binding affinity scores, respectively. The beta (β_0_, β_1_, β_2_, β_3_, β_4_) represents the weights to be determined after training the linear regression model on experimental data. The sign of each beta indicates whether the corresponding explanatory variable is expected to positively or negatively influence the probability of an epitope being immunogenic. The magnitude of each beta shows the relative strength of association between each predictor variable and the probability of an epitope being immunogenic.

(2) $$\pi^{*}=\beta_{0}+\beta_{1}ɑ+\beta_{2}Ɣ+\beta_{3}\delta +\beta_{4}\varepsilon$$

In the logistic regression model above,

(3) $$\pi^{*}=ln(\pi /1-\pi )=ln(P(Y=1)/P(Y=0))$$

where $\pi^{*}$ represents the log-odds of a successful epitope. π and P(Y = 1) are both defined as the probability of an epitope successfully inducing an immunogenic response. P(Y = 0) represents the probability of an epitope unsuccessfully inducing an immunogenic response. Similar to Equation (1), ɑ, Ɣ, δ, and ε represent an epitope’s predicted immunogenicity, antigenicity, allergenicity, and binding affinity scores, respectively. The beta (β_0_, β_1_, β_2_, β_3_, β_4_) represents the weights to be determined after training the probabilistic logistic regression model on experimental data. The sign of each beta indicates whether the corresponding explanatory variable is expected to positively or negatively influence the log-odds of an epitope being immunogenic. The magnitude of each beta shows the relative strength of association between each predictor variable and the log-odds of an epitope being immunogenic. 

Based on the models shown in Equations (1)–(3), we developed two scoring functions per regression model, one for MHC Class I epitopes and one for MHC Class II epitopes, resulting in four final scoring functions. We first created the training datasets for the linear regression models by incorporating a beta–binomial distribution to derive an epitope’s immunogenic potential from the amount of successful and unsuccessful experimental T-cell assay trials. The experimental T-cell assay data used to train these models were taken from the IEDB for both Class I and II epitopes [24]. We gathered data on the number of T-cell assay trials performed and the number of trials that succeeded in eliciting a positive response. These two pieces of data were labeled as “Tested” and “Responded” in the training datasets, respectively. Training data epitopes that had more positive T-cell assay results compared to negative were assigned higher potentials, while epitopes that had no positive or mostly negative results were assigned lower potentials on a scale from 0 to 1 using the beta–binomial distribution. This method, further explained by DeepImmuno, allowed us to create a continuous dependent variable for our epitope training data, known as the “Potential” [40]. This variable, represented by “Y” in Equation (1), represents the probability of an epitope eliciting a positive T-cell response. We aimed to prioritize mutated epitopes that shared similar characteristics with training data epitopes demonstrating high potentials.

We employed slightly different methods than described above to collect training data for the MHC Class I and II probabilistic logistic regression models. We collected experimental T-cell assay data from NEPdb for Class I epitopes and IEDB for Class II epitopes [29,41]. The training data epitopes were customized to align closely with the parameters of the mutated epitopes in our study. Thus, on NEPdb, we searched for only HLA-A and HLA-B epitopes with both positive and negative responses and all tumor types. The search parameters on IEDB included linear epitopes, both positive and negative T-cell assay data, MHC Class II epitopes, and human hosts. Before the data cleaning process, we kept three key characteristics for each epitope: peptide sequence, T-cell assay results (positive or negative), and MHC allele restriction. Unlike the linear regression model training datasets, the T-cell assay results were used as a binary dependent variable for training our probabilistic logistic models.

Following the training data retrieval for both the linear regression and logistic regression models, a thorough cleaning process was conducted. For MHC Class I epitopes, only 9 and 10mers were retained, whereas Class II epitopes were filtered to keep 15mers. Any peptide sequences that exceeded 15 amino acids were truncated to the first 15 amino acids. Furthermore, sequences were examined to ensure that they comprised only the 20 standard amino acid letter codes. Only epitopes that had MHC restrictions as part of the 27-allele HLA reference set were included. As mentioned above, this process was undertaken to align the training data as closely as possible to our mutated epitope data, ensuring precisely calculated weights for our final scoring functions. 

Next, we collected binding affinity, immunogenicity, antigenicity, and allergenicity values as the input variables for each epitope in the training datasets using the tools described in Section 2.1. After obtaining all of the required characteristics for each epitope, we normalized all values to reduce the impact of outliers and ensure consistent data ranges among the variables. We performed this process because the discrepancy in data ranges would significantly compromise the accuracy of the weights and resulting score potential. For instance, the predicted immunogenicity of MHC Class I epitopes produced a decimal number ranging from approximately −0.3 to 0.4, while the binding affinity values could span from 10 nM to 50,000 nM. The difference in these ranges would have drastically decreased the model’s overall accuracy. For the immunogenicity, antigenicity, and allergenicity of epitopes, we took the z-score of all the values and then performed a min–max normalization on the data to keep the ranges between 0 and 1. Class II immunogenicity scores and all allergenicity scores depicting non-allergens were characterized by lower values. Given our preference for non-allergenic epitopes, we modified the normalization process to align with this preference: lower scores were assigned values closer to 1, while higher scores were assigned values closer to 0. To normalize the binding affinity values, we used a natural log transformation of the data to create a more normal distribution and limit the range. The final normalized training datasets for the linear and logistic regression models are included in Supplementary Tables S1–S4.

To derive the weights of Class I and II scoring functions for both the linear and logistic regression models, we trained Equations (1) and (2) on the final training datasets using functions from the sklearn library. We analyzed the performance of all the scoring functions through a 5-fold cross-validation technique with a 70:30 training to test set split. After comparing the performance of the linear regression scoring functions to the probabilistic logistic regression functions, we observed that the Class I and II linear regression functions exhibited low R^2^ values, while the Class I and II logistic regression functions performed at moderately high accuracies. As a result, AutoEpiCollect uses only the MHC Class I & II probabilistic logistic regression scoring functions to rank the probability of each mutated epitope eliciting a positive T-cell response. Only the epitopes that rank within the top 20 for each point mutation undergo the next steps of individual variable filtration.

### 2.3. Individual Variable Filtration Using AutoEpiCollect

After data collection and ranking for all the desired epitope characteristics are complete, AutoEpiCollect places individual filters on the mutated epitopes based on instability, half-life, toxicity, and IFN-γ release criteria. The exclusion criteria for the individual parameters are shown in Table 3.

A threshold of less than 40 is used for filtering the instability index, as this aligns with ProtParam’s approach [37]. Additionally, we set a minimum half-life of 1 h for filtration, as this duration corresponds with the minimum half-life of amino acids observed in mammals, typically ranging from 0.8 to 1 h [6]. While filtering, the aliphatic index, GRAVY score, and isoelectric point are not considered; however, it is essential to acknowledge that these physical properties are important factors to consider when designing experiments involving immune responses to selected epitopes. IFN-γ results are considered for only MHC Class II epitopes, since IFNepitope is trained on only Class II epitope data [39].

### 2.4. Obtaining Population Coverage with AutoEpiCollect and PCOptim/PCOptim-CD

The selected top epitopes from each point mutation and cancer type, which successfully pass the ranking and individual variable filtration processes above, undergo further analysis using IEDB’s downloadable Population Coverage Epitope Analysis Tool [42]. Using this tool, AutoEpiCollect facilitates the calculation of MHC Class I and II vaccine coverages for top-filtered epitopes on world and regional populations. Since HLA allele representation varies across different ethnicities and geographical regions, a higher population coverage implies that our pan-cancer vaccine has the potential to impact a larger number of individuals, making it more applicable.

To optimize our datasets by minimizing the number of alleles while maximizing population coverage, AutoEpiCollect uses our PCOptim and PCOptim-CD programs for Class I and Class II epitopes, respectively [6,7]. However, IEDB’s population coverage tool has limitations concerning a restricted set of MHC Class II alleles, as shown in Table 4 [42]. Consequently, before submitting the top MHC Class II epitopes into PCOptim-CD, AutoEpiCollect excludes certain epitopes with alleles unsupported by the population coverage tool. After obtaining the optimal epitope/HLA allele combinations from PCOptim and PCOptim-CD, AutoEpiCollect inputs these data into IEDB’s Population Coverage Tool to calculate the optimized vaccine population coverage and average allele hit for the world population and major regions.

### 2.5. Determining Cancer-Specific PIK3CA Point Mutations 

**Figure 2:**
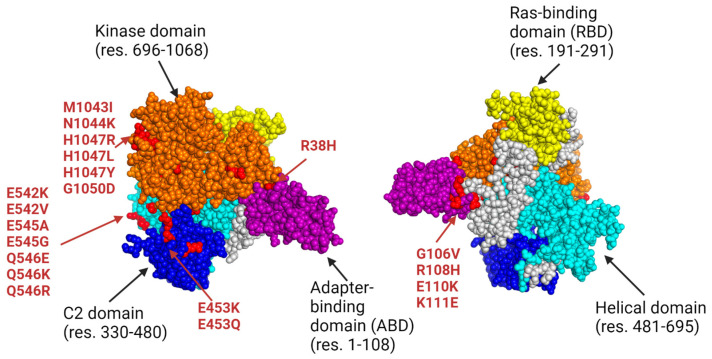
Annotated 3D structure of the p110ɑ protein with point mutations marked in red (RCSB PDB: 4L2Y). The p110ɑ protein consists of five different protein domains that regulate the kinase activity of the protein: adapter-binding domain (ABD), Ras-binding domain (RBD), C2 domain, helical domain, and kinase domain [43]. The unlabeled gray areas represent linker residues that join domains together. All mutated residues used in this study are highlighted in red on their corresponding domains [16,17,18,19,20,21,22]. The most frequently observed mutations associated with cancer are specifically labeled to indicate their affected domains. Out of the five domains, the RBD holds no common sites of mutation. The C2 domain and the ABD are equally susceptible to point mutations, with mutations commonly occurring around residues 110 and 453 [43]. However, most of the PIK3CA hotspot mutations are located on the kinase and helical domains and generate gain-of-function mutations, leading to unregulated phosphorylation and cell signaling [38].

After developing AutoEpiCollect’s novel pan-cancer vaccine design software, we applied its design by targeting cancer subtypes susceptible to PIK3CA mutations. Common cancer-causing mutations on the PIK3CA gene are clustered on exon 9 of the helical domain and exon 20 of the kinase domain [17]. The most prevalent point mutations leading to cancer are mutations E542K, E545K, and H1047R [17]. We selected point mutations for this study based on prior studies reporting clinical data on PIK3CA mutations observed in CRC, meningioma, BC, EC, and GBM tissue samples [16,17,18,19,20,21,22]. Figure 2 shows the p110ɑ protein encoded by the PIK3CA gene, with its domains highlighted and common point mutations annotated in red. The pdb 3D model of this protein is located in the PDB Models folder within the Supplementary Files. Table 5 lists all PIK3CA gene point mutations used in this study, each associated with one or more cancer types. The gene name, common cancer subtypes, and prevalent point mutations of PIK3CA were all entered into AutoEpiCollect. In return, we received .xlsx files of the top predicted immunogenic PIK3CA MHC Class I and II epitopes and optimized epitope lists with population coverage results.

### 2.6. Three-Dimensional (3D) Analysis Using Peptide-HLA and TCR Complex Modeling

We modeled three peptide-HLA complexes and three peptide-HLA-TCR complexes to further analyze the binding potentials and TCR interactions of top PIK3CA epitope output by AutoEpiCollect. The HLA sequences were obtained through the IMGT/HLA database and input into the Swiss Model to perform homology modeling, which compared the original sequence to similar templates. The Swiss Model takes in amino acid sequences as input and searches for templates obtained from its library, ExPDB, which removes unreliable models and splits the remaining protein chains for alignment from the PDB database [44,45]. Factors considered for an ideal template include sequence similarity and model quality. We chose a template with the highest sequence similarity (at least 0.62). Once the output model was created, the Swiss Model provided information regarding the stability and reliability of the model by conducting a Ramachandran plot analysis to validate the results further. We downloaded and edited HLA structures on PYMol. These edited structures were then input into MDockPeP, where peptide-HLA binding was performed [46]. We used MolProbity to calculate the clashing score and Ramachandran plot parameters to choose the best model. Models with the lowest clashing score and Ramachandran outliers were kept [47].

Using TCRModel, we modeled the peptide-HLA-TCR complexes with the same peptide-HLA complexes’ structures discussed above and the TRAV/TRBV/TRAJ/TRBJ/CDR3 TCR gene sequences from VDJdb based on HLA type [48,49]. The models with the lowest clash scores and Ramachandran outliers calculated with MolProbity were chosen to model the peptide-HLA-TCR complexes.

## 3. Results

### 3.1. Overall Methodology Workflow

**Figure 3:**
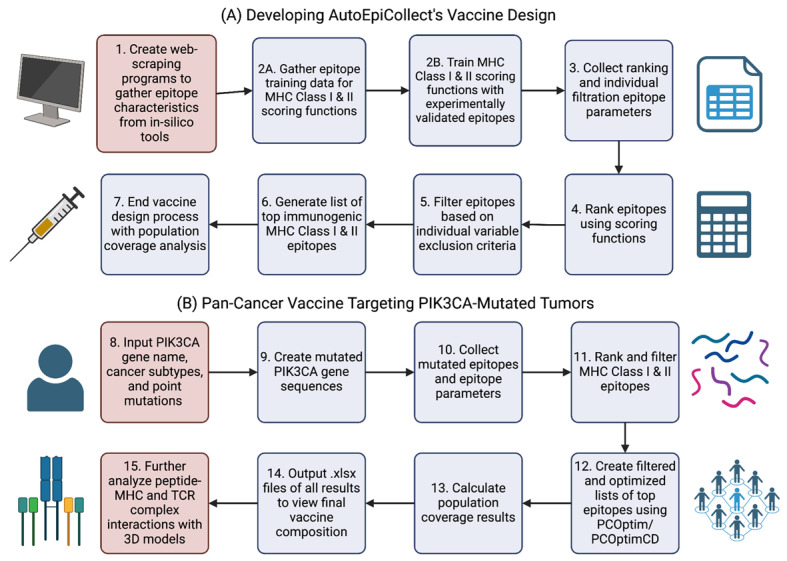
The overall methodology flowchart for the study. (**A**) is the workflow chart for AutoEpiCollect’s vaccine design process. (**B**) shows AutoEpiCollect’s vaccine design process applied to a pan-cancer vaccine targeting PIK3CA-mutated tumors. The steps in red boxes are processes done without AutoEpiCollect’s help, while the purple steps are automated.

Figure 3A illustrates the steps taken to create AutoEpiCollect’s pan-cancer vaccine design. We initiated the process by creating web-scraping programs to gather gene sequences and epitope characteristics. These programs played a role in gathering both experimental and in silico epitope training data for the MHC Class I and II linear regression and probabilistic logistic regression scoring functions. The scoring functions were then trained on the epitope training data. We decided to make AutoEpiCollect rank epitopes with the logistic regression scoring functions due to higher performance metrics than the linear regression models. The web-scraping programs mentioned in the first step were integrated into AutoEpiCollect to automatically collect the in silico characteristics needed for epitope selection. Using these epitope characteristics, we ranked the mutated Class I and II epitopes with the scoring functions and further filtered the data based on exclusion criteria. The combination of epitope ranking and individual variable filtration generated our list of top immunogenic MHC Class I and II epitopes. We completed AutoEpiCollect’s vaccine design by creating a population coverage tool using PCOptim/PCOptim-CD and IEDB’s Population Coverage Analysis tool. AutoEpiCollect’s final pan-cancer vaccine design algorithm is a combination of all these tools. 

**Figure 4:**
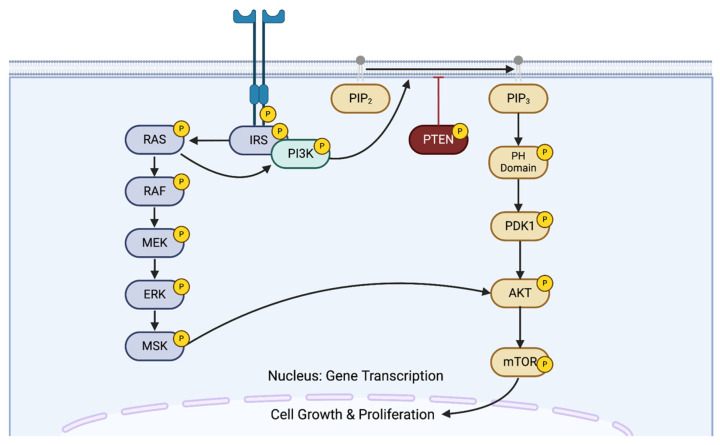
PI3K/Akt/mTOR intracellular signal transduction pathway. This pathway facilitates multiple cellular functions, such as cell growth and proliferation. Upon binding with growth factors, cytokines, or hormones, tyrosine residues on receptor tyrosine kinases (RTKs) undergo autophosphorylation, allowing the p85 subunit of PI3K to bind to the RTKs. Via the p110ɑ subunit, PI3K catalyzes the phosphorylation of the lipid PIP2, producing PIP_3_, which serves as a secondary messenger within the signal cascade for proteins such as Akt. The phosphatase PTEN plays a pivotal role in suppressing this pathway by dephosphorylating PIP_3_ [50].

This study applied AutoEpiCollect’s final vaccine design to develop a pan-cancer vaccine targeting the PIK3CA gene. We chose this gene due to its widespread prevalence across multiple cancer types. Furthermore, PIK3CA plays an important role in the PI3K/Akt/mTOR intracellular signaling pathway. Figure 4 shows the full pathway in greater detail. Mutations affecting PIK3CA upregulate the kinase activity of the PI3K protein, leading to uncontrolled cell growth and proliferation [50]. After determining common PIK3CA-mutated cancer types and prevalent cancer-inducing PIK3CA point mutations, we input these cancers and mutations into AutoEpiCollect. Figure 3B depicts the steps taken by AutoEpiCollect to create a pan-cancer vaccine with this data. After generating point-mutated gene sequences, AutoEpiCollect produced mutated epitopes and gathered epitope characteristics. It then ranked and filtered Class I and II epitopes using the trained probabilistic logistic regression scoring functions coupled with individual variable filtration. 

The lists of top immunogenic MHC Class I and II epitopes were optimized for population coverage using PCOptim and PCOptim-CD. AutoEpiCollect inputs the filtered and optimized lists of predicted immunogenic epitopes into the population coverage analysis tool to calculate the filtered and optimized population coverages of a pan-cancer vaccine containing mutated PIK3CA epitopes. The final lists of the top MHC Class I and II epitopes and population coverage results were output as .xlsx files for us to analyze. To further analyze epitope binding potential and TCR complex interactions, we created peptide-HLA and peptide-HLA-TCR models with three selected immunogenic epitopes. From our overall methodology process, we received results regarding the final weights and performances of Class I and II scoring functions, the final list of top epitopes generated by AutoEpiCollect, the calculated pan-cancer vaccine population coverage results, and the 3D peptide-HLA and peptide-HLA-TCR complex interactions.

### 3.2. Linear Regression Model Weights and Performance

The Class I and II linear regression model training datasets each contained 1370 epitopes. Equations (4) and (5) show the final linear regression scoring functions with the weights assigned to each variable after training. Equation (1) provides more information on what each variable in the function represents:

(4) $$Y_{MHCI}=0.335+0.142ɑ+0.457Ɣ-0.072\delta -0.005\varepsilon$$

(5) $$Y_{MHCII}=0.681+0.149ɑ-0.016Ɣ+0.134\delta -0.029\varepsilon$$

Looking at Equations (4) and (5), we can see that the Class I scoring function assigns higher probabilities to epitopes with higher antigenicities and higher allergenicities. The Class II scoring function assigns higher probabilities to epitopes with lower antigenicities and lower allergenicities. Both the Class I and II scoring functions assign higher probabilities to epitopes with higher immunogenicities and lower binding affinities. The prioritization of antigenicity and allergenicity scores are inconsistent between the two functions. These differences are likely due to inconsistencies in the training data for the two scoring functions. This is shown by the positive weight assigned to these epitope characteristics. To assess the performances of these two functions, we ran a 5-fold cross-validation process with a 70:30 training to test split using the sklearn library. The training and validation sets for the linear regression models consisted of scores for the four distinct epitope characteristics mentioned above, as well as a predicted probability of an epitope eliciting a positive T-cell response based on the number of times an epitope elicited a T-cell response during multiple experimental T-cell assays. The linear regression models aimed to accurately predict immunogenic potential, based on a linear combination of the four epitope characteristics. Therefore, R^2^ was chosen as the best metric to evaluate the fit of the models. The cross-validation process revealed R^2^ values of 0.060 for the Class I function and 0.028 for the Class II function. 

### 3.3. Probabilistic Logistic Regression Scoring Function Weights and Performance

We employed two probabilistic logistic scoring functions, each involving four variables, designed for MHC Class I and II epitopes. Like the linear regression models, one scoring function was trained on MHC Class I data, while the other scoring function was trained on Class II data. The training dataset for MHC Class I contained 357 epitopes, while the Class II training dataset contained 713 epitopes. Equations (6) and (7) show the final scoring functions with weights derived after training the probabilistic logistic regression model. Equation (2) discusses what each variable in the scoring functions represents:

(6) $${\pi_{MHCI}}^{*}=2.311+0.893ɑ+0.197Ɣ-3.426\delta -0.479\varepsilon$$

(7) $${\pi_{MHCII}}^{*}=0.654-0.010ɑ+0.381Ɣ+0.831\delta -0.096\varepsilon$$

The equations above reveal that for the Class I scoring function, high immunogenicity scores and high allergenicity scores lead to high epitope probabilities, while high probabilities are assigned to Class II epitopes with low immunogenicity and low allergenicity scores. High antigenicity values and low binding affinities are prioritized in both the Class I and II probabilistic logistic regression scoring functions. The inconsistencies between the Class I and II scoring functions are likely due to the different in silico tools used to calculate the Class I and II immunogenicity and allergenicity training data. Next, we evaluated the scoring functions through a 5-fold cross-validation process with a 70:30 training to test set split using sklearn. The training and validation sets for the probabilistic logistic regression models were slightly different than the linear regression sets. Instead of a predicted probability, these datasets contained a straightforward binary value, indicating whether an epitope elicited a T-cell response during experimental T-cell assays. Due to this classification approach, AUC and overall dataset accuracy were chosen as the performance metrics for the probabilistic logistic regression models. Our analysis revealed the following performance metrics for the Class I scoring function: an accuracy of 0.76 and an AUC of 0.73. The Class II scoring function exhibited slightly different performance indicators, with an accuracy of 0.56 and an AUC of 0.62. Compared to the low R^2^ values for the Class I and II linear regression scoring functions, the probabilistic logistic regression scoring functions exhibited more robust performance metrics. As a result, we decided to incorporate solely the MHC Class I and II probabilistic logistic regression scoring functions into AutoEpiCollect’s epitope ranking algorithm.

### 3.4. Operating AutoEpiCollect’s Graphical User Interface

**Figure 5:**
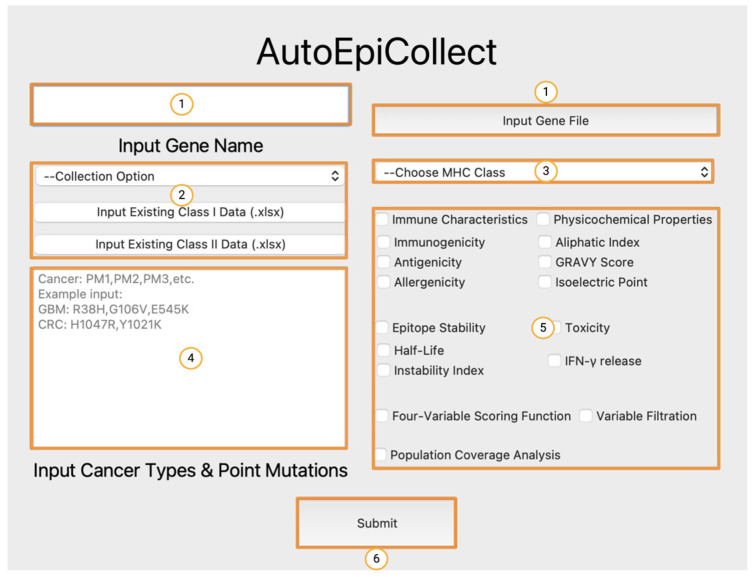
Home screen of AutoEpiCollect user interface. Users must first input their gene name or gene file into the boxes labeled 1, followed by entering a collection option in the area labeled 2, and the desired epitope MHC Class in the dropdown menu labeled 3. The text box labeled 4 is where the cancers and genes of interest must be input. The user can toggle different epitope data collection settings by checking the boxes in the area labeled 5 before clicking the “Submit” button labeled 6 to begin running the program.

**Figure 6:**
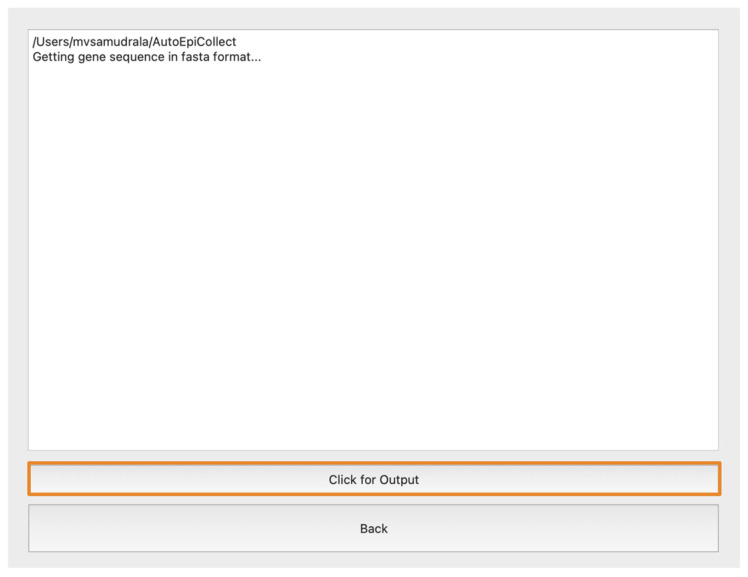
Output screen of AutoEpiCollect. The software’s progress is displayed in real time in the textbox for the user to view. When the background program states that it is complete, the user can click on the “Click for Output” button to display the top epitopes gathered by AutoEpiCollect. Clicking the “Back” button returns the user to the home screen.

To operate the AutoEpiCollect GUI, users only need to input the target gene, relevant point mutations with cancer subtypes, and desired collection options into the numbered fields labeled in Figure 5. Moreover, users can add to existing epitope data to target additional point mutations. This is done by selecting the “Update Existing Data” option from the dropdown menu in the area labeled 2 and inputting a .xlsx with a list of point mutations and top epitopes previously output by AutoEpiCollect. There are a variety of epitope characteristics to choose from when designing a pan-cancer vaccine using AutoEpiCollect. These characteristics address the safety, stability, and immunogenic properties of the top epitopes. Only the characteristics the user selects will be considered when designing the vaccine. Population coverage analysis is also optional but recommended for further analysis of the applicability of the final vaccine. At its completion, AutoEpiCollect will output .xlsx files of final epitope data and population coverage results for a pan-cancer vaccine containing the predicted immunogenic epitopes. The software’s progress can be monitored through the output page shown in Figure 6. A complete and detailed installation/user guide of AutoEpiCollectGUI can be found on the documentation website under the “About this project” section at https://github.com/mvsamudrala/AutoEpiCollect (accessed on 2 February 2024).

### 3.5. Top Immunogenic PIK3CA-Mutated Epitopes Identified by AutoEpiCollect

After analyzing 49 PIK3CA point mutations across five different cancer subtypes, we received 361 potentially immunogenic MHC Class I epitope/HLA pairs and 219 MHC Class II epitope/HLA pairs. Supplementary Tables S5–S14 show the unfiltered and unranked MHC Class I and II epitope/HLA pairs by cancer subtype. Supplementary Tables S15 and S16 detail the final lists of predicted immunogenic Class I and II epitopes by point mutation, respectively. Table 6 presents an overview of the number of point mutations that generated MHC Class I and II immunogenic epitopes. Certain point mutations led to the generation of both Class I and Class II epitopes, indicating that vaccines incorporating these epitopes may elicit both CD8^+^ and CD4^+^ T-cell responses in patients. 

### 3.6. Pan-Cancer Population Coverage Results for MHC Class I and II Epitopes Calculated by AutoEpiCollect

AutoEpiCollect calculated the world and regional population coverage for a pan-cancer vaccine containing all the potentially immunogenic epitopes outlined in this study. Supplementary Tables S19–S22 show the full filtered and optimized population coverages and average hits for MHC Class I and II epitopes. The optimized lists of Class I and II epitope/HLA pairs used to calculate the optimized population coverage results are in Supplementary Tables S17 and S18, respectively. Furthermore, the filtered and optimized MHC Class I and II population coverage graphs can be found in Supplementary Figures S1–S4. 

The world coverage for the Class I epitope dataset reached an optimized value of 98.09%, whereas the Class II dataset achieved an optimized coverage of 81.81%. Table 7 shows the world population coverage results for filtered and optimized epitope datasets. Population coverage refers to the predicted percentage of the population who have HLA alleles covered by the vaccine. The average epitope hit is the average number of epitope/HLA combinations recognized by the population. PC90 refers to the minimum number of epitope/HLA combinations recognized by 90% of the population. The Class I data exhibited an average world and regional coverage of 88.92%, with Europe attaining the highest coverage at 99.45% and Central America having the lowest at 7.76%. The Class II data showed lower coverage, with an average world and regional coverage of 65.57%. North America exhibited the highest coverage at 87.89%, while South Africa had the lowest coverage at 32.1%. The optimized Class I dataset had an average of 3.82 epitope/HLA combinations recognized by the world population and a minimum number of 2.02 epitope/HLA combinations recognized by 90% of the world population. The optimized Class II dataset had an average of 1.11 epitope HLA/combinations recognized and a minimum number of 0.55 epitope/HLA combinations recognized by 90% of the world population. When assessing the population coverage of MHC Class II epitopes and HLA pairs using the IEDB Population Coverage Analysis tool, the web tool did not identify certain HLA alleles. These unaccounted alleles are detailed in Table 5. 

### 3.7. Three-Dimensional (3D) Peptide-HLA and Peptide-HLA-TCR Complex Interaction Results

**Figure 7:**
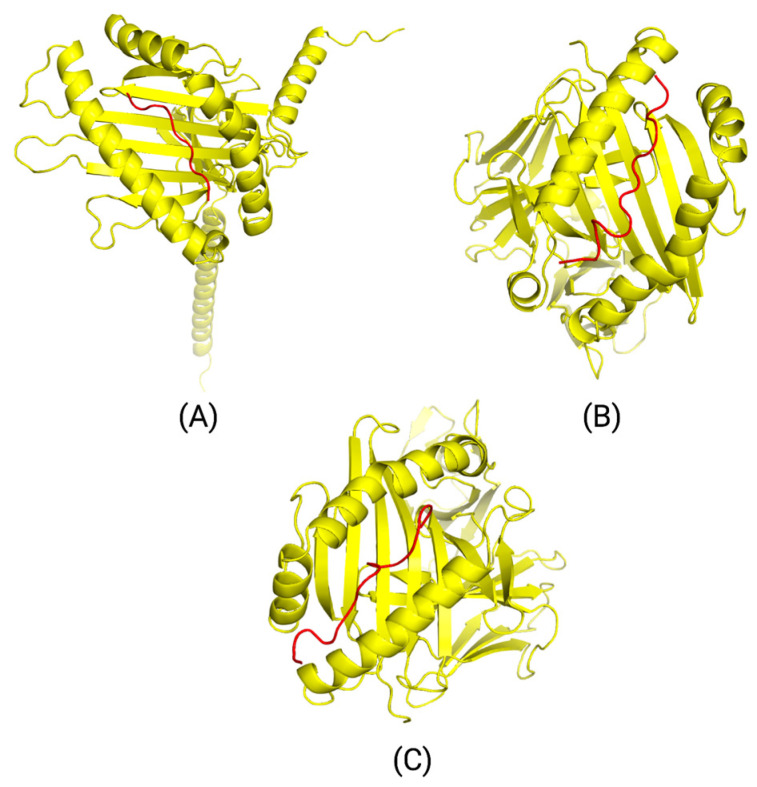
3D models for peptide-HLA complexes of potentially immunogenic epitopes. MHC Class I molecule HLA-A*31:01 interaction with endometrial carcinoma epitope AHHGDWTTK (**A**). MHC Class II molecule HLA-DPA1*01:03/DPB1*02:01 interaction with endometrial carcinoma epitope DWTTKMDWIFHTIKQ (**B**). MHC Class II molecule HLA-DQA1*05:01/DQB1*03:01 interaction with colorectal adenocarcinoma epitope HGLQDLLNPIGVTGS (**C**). Red represents the peptide bound to the yellow HLA allele. These three models show relatively linear peptide binding efficiently to the grooves of the HLA allele.

Three peptide-HLA interactions were modeled through MDockPep using the HLA template from the Swiss model: AHHGDWTTK binding to HLA-A*31:01, DWTTKMDWIFHTIKQ binding to HLA-DPA1*01:03/DPB1*02:01, and HGLQDLLNPIGVTGS binding to HLA-DQA1*05:01/DQB1*03:01. The peptide-HLA models are shown in Figure 7, along with their .pdb 3D-coordinate files located in the PDB Models folder within the Supplementary Files. The corresponding HLA alleles and peptides were then bound to TCR complexes through TCRmodel. The TCR α/β sequences corresponding to each HLA allele are included in Supplementary Table S26. 

**Figure 8:**
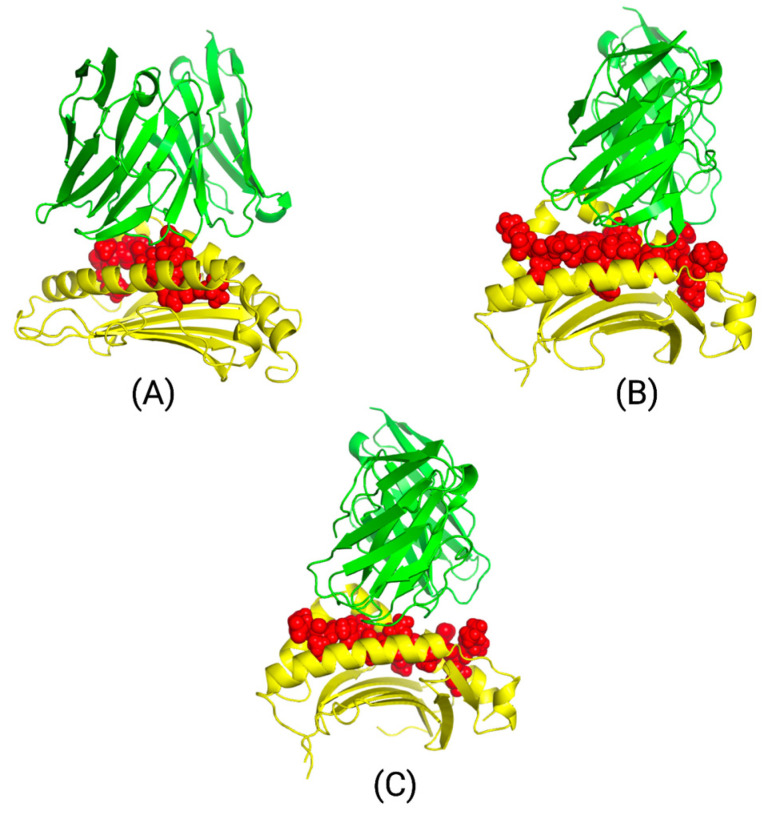
HLA-A*31:01 interaction with peptide AHHGDWTTK and TCR receptor (**A**). HLA-DPA1*01:03/DPB1*02:01 interaction with peptide DWTTKMDWIFHTIKQ and TCR receptor (**B**). HLA-DQA1*05:01/DQB1*03:01 interaction with peptide HGLQDLLNPIGVTGS and TCR receptor (**C**). Green represents the TCR complex α/β chains bound to its corresponding yellow HLA allele. Red represents the peptide. Based on the models, we can see that peptide-HLA complexes bind effectively to the TCR complexes.

The finalized peptide-HLA-TCR models, along with their corresponding .pdb 3D-coordinates, are depicted in Figure 8 and can be found in the PDB Models folder within the Supplementary Files. The MolProbity scores verifying the binding quality of each TCR complex structure are located in Supplementary Tables S23–S25. Epitope AHHGDWTTK bound to HLA-A*31:01 and its corresponding TCR complex had a clashing score of 0.71, resulting in less steric strain in the structure. MolProbity uses a reference set of high-resolution protein structures to compare the input to and provides a Rama z-score that represents how the structure deviates from the “gold standard” structures [51]. This indicates that the protein overall is folded accurately, though it has Ramachandran outliers that could result from unfavorable backbone or side-chain conformations, among other issues. Ramachandran outliers can be removed by utilizing programs such as Phenix that refine macromolecular structures [52]. Models B and C have optimal clashing scores, Ramachandran outliers, favored residues, and Rama z-scores, indicating a stable structure that has properly folded with minimal backbone and side-chain issues.

## 4. Discussion

AutoEpiCollect was developed to accurately predict safe and potentially immunogenic MHC Class I and II epitope sequences. This tool is specifically designed for the expedited development of pan-cancer vaccines targeting any oncogene of interest. AutoEpiCollect aims to accelerate the tedious task of epitope collection and analysis, with our vaccine design ultimately allowing the validation of a greater number of pan-cancer vaccines in clinical settings. Previous cancer vaccine designs subjected mutated epitopes targeting one cancer subtype of interest to manual variable filtration based on a predetermined set of exclusion criteria [6,7,8,9,10]. However, these methods are time-consuming and cannot efficiently analyze a large set of mutated epitopes. It is crucial that vaccines are capable of targeting a broad spectrum of mutations, since tumors mutate rapidly. We attempted to mitigate this issue with our deep learning-based method, IntegralVac. While IntegralVac is a powerful machine learning-based program that drastically increases the accuracy of MHC Class I in silico predictions, it is missing a comprehensive Class II prediction feature. This restricts the applicability of vaccines predicted with IntegralVac [11]. Stranzl et al. also tried to improve upon individual variable filtration through a tool called NetCTL. NetCTL identifies epitopes and ranks them according to their MHC Class I binding affinity, Transporter Associated with Antigen Processing transport efficiency, and proteasomal cleavage [12]. Although this tool prioritizes the overall effectiveness of an epitope’s binding affinity, transport, and ability to be broken down, it does not consider immunogenicity, antigenicity, allergenicity, stability, and toxicity. These variables play a major role in predicting whether or not an epitope will induce a strong immune response without triggering an allergic reaction [29,30].

AutoEpiCollect’s mutated epitope selection process incorporates a combination of machine learned-based ranking and individual variable filtration, as demonstrated by the studies discussed above [6,7,8,9,10,11,12]. We subjected epitopes to a novel ranking system using a probabilistic logistic regression model that was trained on experimentally validated epitopes. Unlike a conventional binary logistic regression classification of immunogenic or non-immunogenic, our model ranked epitopes with a probabilistic logistic regression function based on the probability of triggering a safe immune response. This prioritization is rooted in clinically relevant factors, including immunogenicity, antigenicity, allergenicity, and binding affinity, all of which are crucial determinants for the safety and efficacy of cancer vaccines [29,30]. The choice of a ranking algorithm over a classification approach stems from the understanding that epitope selection is a nuanced process, ultimately determined by researchers conducting clinical trials for vaccine testing. Instead of studying a definitive set of epitopes, researchers can use the epitopes generated by AutoEpiCollect as a starting point to identify the epitopes with the highest likelihood of eliciting an immune response. 

However, before finalizing the epitope ranking algorithm, we compared the performances of linear regression scoring functions and probabilistic logistic regression scoring functions to determine which regression model would be the best to incorporate into AutoEpiCollect. The Class I and II linear regression scoring functions had R^2^ values of 0.060 and 0.028, respectively. The probabilistic logistic regression scoring function for Class I epitopes performed at an accuracy of 76% (AUC = 0.73), while the function for Class II epitopes performed at an accuracy of 56% (AUC = 0.62) with the test data. Based on these metrics, we realized that the linear regression scoring functions were not viable for epitope prediction. The low R^2^ values for the linear regression functions may be attributed to the lack of experimentally procured epitope characteristics. The immunogenicity, antigenicity, allergenicity, and binding affinity scores for each training data epitope are based on in silico prediction tools that use the epitope’s peptide sequence in some way to calculate scores. The training data from the IEDB solely reports experimentally validated T-cell assay data. As a result, the training data matches up experimental T-cell data with virtual epitope characteristics. In addition, we utilized DeepImmuno’s beta–binomial distribution approach to compute the probability of an epitope eliciting a T-cell response based on the number of successful and unsuccessful experimental T-cell assays [40]. This was done with the hope of placing a higher priority on training data epitopes that elicited more positive T-cell responses. However, this virtual computation combined with the virtual epitope characteristics may have built-in inconsistencies within the training data for the linear regression models, leading to a lack of fit for both the Class I and II scoring functions.

While the metrics for the probabilistic logistic regression scoring functions were a significant improvement over the linear regression metrics, there were still some discrepancies in accuracy observed between Class I and II epitopes. This can be traced back to distinctions in the in silico prediction tools used for each class. Notably, we collected Class I and II binding affinity, immunogenicity, and allergenicity epitope data from different tools, leading to inherent differences in the training datasets. In essence, while operating within the same program, the Class I and II training data collection processes are distinct tools, and the discrepancy in model accuracy emerges from all the differences in the two data collection approaches. In addition, IEDB’s CD4 Immunogenicity tool only calculates the immunogenicity of 15mers peptides. Thus, we limited the training data to only 15mers when training our Class II scoring function. This reduced diversity of the Class II training dataset led to homogeneity, potentially contributing to lower model accuracy. Despite these limitations, our ranking algorithm outputs the relative immunogenic potential of an epitope. This approach allows for a more flexible and informed selection process during the early stages of vaccine development. 

Following ranking, Class I and II epitopes underwent individual variable filtration following the same process we previously used, based on half-life, instability, toxicity, and IFN-γ release [6,7]. This filtration method relied on exclusion criteria rather than a ranking system due to the crucial nature of these variables. The half-life and instability of an epitope are indicative of the effectiveness of the elicited T-cell response. Regardless of an epitope’s immunogenic ability, a low half-life and unstable nature will significantly decrease the sustained immune response, necessitating higher doses and frequent administration. Furthermore, higher doses may cause more potent side effects, contradicting one of the primary objectives of this study. Toxicity and IFN-γ release predictions are binary variables, each characterized as either positive or negative. Similar to half-life and instability, toxicity is important when considering the potential side effects of vaccine administration. Our decision to include solely non-toxic epitopes was driven by the imperative to establish pan-cancer epitope-based vaccines as a powerful immunotherapeutic option for a diverse patient population without compromising on tolerability. We took IFN-γ release predictions into account for MHC Class II epitopes due to the fact that the IFNepitope server is only trained on Class II data. Activated CD8^+^ T-cells, prompted by MHC Class I molecules, spearhead the primary antitumor response, while CD4^+^ helper T-cells, activated by Class II molecules, play a complementary role by enhancing and prolonging CD8^+^ effector function. The secretion of IFN-γ by CD4^+^ T-cells triggers the expression of chemokines necessary for recruiting CD8^+^ T-cells. Furthermore, CD4^+^ T-cells exhibit direct tumor suppression through various IFN-γ functionalities. Consequently, MHC Class II epitopes capable of inducing IFN-γ release prove instrumental in stimulating antitumor effects through multiple mechanisms [53]. Considering this information, we decided to exclusively include predicted IFN-γ-releasing Class II epitopes, aiming to amplify the immunogenic effects of the vaccine.

After finalizing our ranking and individual filtration algorithms, as well as the rest of AutoEpiCollect, we proceeded to test our machine learning-driven vaccine design on a pan-cancer vaccine targeting a common oncogene. Our study focused on the PIK3CA gene due to its pivotal role in the PI3K/Akt/mTOR intracellular signaling pathway, as illustrated in Figure 4. This pathway governs vital cellular processes such as growth, proliferation, and differentiation [50]. Mutations within the PIK3CA gene impact the p110ɑ catalytic subunit of the PI3K protein, disrupting the signaling pathway and leading to immune disorders, cardiovascular diseases, and cancer in various tissue types [50]. After inputting prevalent PIK3CA point mutations into AutoEpiCollect, we received Class I epitopes targeting 34 mutations and Class II epitopes targeting 11 mutations from the total 49 mutations initially input. These Class I and II epitopes demonstrate predicted attributes of robust immunogenicity, safety, stability, and IFN-γ release. Notably, 10 mutations yielded overlapping epitopes for both MHC Class I and II, strengthening our vaccine design. This overlap holds significant potential for eliciting heightened immunogenic responses by activating both CD8^+^ cytotoxic T-cells and CD4^+^ helper T-cells. As illustrated in Figure 2 and Table 6, a substantial portion of the epitopes in the final datasets are directed at mutations found in either the kinase or helical domain of the p110ɑ protein. These domains are recognized as hotspot regions prone to prevalent gain-of-function mutations in the PI3K kinase protein and underlie many different subtypes of cancer [12]. The targeting of these hotspot regions by a large number of our final epitopes enhances our vaccine’s utility and emphasizes its potential efficacy in addressing the major molecular drivers of PIK3CA-mutated cancers. 

Following the optimization of MHC Class I and II epitope datasets using PCOptim and PCOptim-CD, AutoEpiCollect input the epitope/HLA allele pairs into IEDB’s Population Coverage Analysis tool, resulting in world population coverages of 98.09% and 81.81%, respectively. The high Class I epitope coverage underscores the potential for our PIK3CA vaccine to impact various patients worldwide. In addition, it confirms that AutoEpiCollect’s epitope selection method generated an extensive dataset. However, both Class I and II coverages are not uniformly distributed across regions. Population coverage is contingent on the prevalence of specific HLA alleles in certain global regions and the corresponding epitopes that they can bind to. Europe, North America, and East Asia demonstrated the highest population coverages, since our predicted immunogenic epitopes bind to HLA alleles common in these areas. In contrast, Central America exhibited lower population coverages, at 7.76% for MHC Class I alleles and 49.91% for MHC Class II alleles. This discrepancy arises from the tool’s limitation in accounting for the diverse array of HLA alleles prevalent in Central America. Future vaccine designs might consider prioritizing epitopes that bind to alleles prevalent in regions historically characterized by low coverage, thereby maximizing the vaccine’s applicability. Before calculating population coverage for the Class II dataset, many epitope/HLA allele pairs had to be excluded due to constraints within IEDB’s allele dataset. These excluded alleles are detailed in Table 4. As a result, the population coverage for Class II epitope/HLA allele pairs was based on a restricted dataset and may not accurately reflect the coverage that would be attained with the inclusion of all potentially immunogenic epitope/HLA allele pairs.

We modeled the interactions between three top peptides, HLA alleles, and T-cell receptors through TCRmodel and input them into MolProbity to analyze the overall structures. Additionally, we conducted a Ramachandran plot analysis to determine the Ramachandran favored and outlier regions along with the Rama z-score. Ramachandra plot analysis is a representation of the dihedral angles of each amino acid residue. Both phi (Φ) and psi (ψ) dihedral angle combinations were analyzed to determine Ramachandran favored and outlier regions [54]. Shown in Supplemental Tables S23–S25, Models B and C had ideal Ramachandran favored and outlier regions of less than 0.05% and greater than 98%, illustrating energetically favored dihedral angle combinations. Model A had a slightly higher amount of dihedral angle combinations that were energetically unfavorable. However, since the absolute value of the Rama z-score for all three models met the goal of less than 2, this demonstrated that no significant structural issues were present and that the proteins were properly folded. Furthermore, all models had an optimal clashing score at the 90th percentile or higher. Analyzing the structure through MolProbity not only allows for a more accurate assessment of the structure but also allows for greater precision when correcting issues related to the backbone or sidechain of the structure through the use of refinement and energy minimization software [55]. 

Looking at the binding affinity, immunogenicity, and antigenicity of the final PIK3CA Class I and II epitopes, we can see that the top-ranked epitopes are characterized by strong binders with high immunogenic and antigenic potential. Therefore, AutoEpiCollect’s pan-cancer epitope-based vaccine design is predicted to elicit a strong, sustained immune response in patients with PIK3CA-mutated cancer cells. In addition, the high world population coverage for Class I epitopes shows that our pan-cancer vaccine has the potential to impact a large percentage of the population. These results support the applicability of AutoEpiCollect in developing the starting point for a pan-cancer vaccine, drastically reducing the time it takes it obtain epitopes for in vitro or in vivo studies. However, while AutoEpiCollect is a powerful tool for developing a pan-cancer vaccine, it ultimately cannot replace results validated by experimental or clinical data. AutoEpiCollect and our PIK3CA pan-cancer vaccine data should be used in future murine model trials to test the efficacy of our novel vaccine design and strengthen future epitope selection methods. 

## 5. Limitations 

AutoEpiCollect produced ranked Class I and II epitopes through a probabilistic logistic regression scoring function. While the Class I function demonstrated relatively high rates of accuracy at 76%, the Class II function had a significantly lower rate of 56%, due to epitope size constraints placed by in silico tools when collecting the training data. The model was ultimately trained based on limited experimental T-cell assays that were filtered to only include 15mers. This contributed to a lower diversity, as a significant portion of training data epitopes were removed. The lack of experimental data was also an issue when training the linear regression scoring functions, leading to low R^2^ values. Another limitation that affected the Class II dataset was the set of HLA alleles unaccounted for by IEDB’s population coverage analysis tool. Many of the top Class II epitopes output by AutoEpiCollect were restricted by the HLA alleles shown in Table 4. As a result, they were not included in the Class II population coverage analysis, leading to an underestimated world population coverage of 81.81%.

As our study also primarily focused on missense point mutations, the epitopes chosen only included a small portion of mutations. Mutations such as frameshift and deletion mutations were not considered in AutoEpiCollect. However, as mutations in the PIK3CA gene generally result in mosaicism or missense mutations in somatic cells, the software we created allows for the most common type of mutation to be targeted [56]. As we only focused on one particular gene to determine the quality and accuracy of AutoEpiCollect, it assumes homogeneity across different cancer types. 

With the epitopes derived from point mutations prevalent in our targeted cancer populations, we identified three top epitopes to model the interaction between the epitopes, HLA alleles, and T-cell receptors. All interactions portrayed energetically favored contributions with minimal clashes, excluding model A. The scores of these interactions are shown in Supplemental Tables S23–S25. Though model A was properly folded overall, it had less than optimal Ramachandran outlier regions. These regions demonstrate energetically unfavorable dihedral angle combinations, contributing to a slightly weakened backbone. To address this issue, macromolecular refinement software, such as Phenix, can be utilized, along with energy minimization software [52]. Analysis of these structures in MolProbity allows for more precise refinement. Moreover, it is crucial to highlight that modeling of the interactions between HLA alleles, epitopes, and T-cell receptors cannot accurately illustrate actual interactions within patients. With heterogeneity across different cancers and immune systems, in vitro and in vivo studies would need to be performed to reveal authentic interactions. 

## 6. Future Directions

AutoEpiCollect’s scoring functions rank epitopes based on experimentally validated T-cell data and in silico prediction tools for epitope characteristics. While probabilistic logistic regression scoring functions aim to leave final epitope selection up to researchers designing their own experimental studies, it is important to improve the accuracy of the overall model using updated experimental data. We plan to revamp the MHC Class I and II scoring functions solely with experimentally validated training data. Our goal is to boost the accuracy of the models to above 90%. In addition, we want to expand AutoEpiCollect’s capabilities to include other types of gene mutations such as deletions and insertions. Although these mutations are uncommon in PIK3CA, they could be prevalent in other common oncogenes. Adding this capability would significantly increase the applicability of AutoEpiCollect and widen the number of potential cancer types. 

The next step for validating AutoEpiCollect’s vaccine design process is through murine trials. Therefore, we will incorporate epitope selection methods targeting murine MHC molecules and oncogenes. This includes adding extra options to AutoEpiCollect’s GUI for users to toggle between human and murine epitope selection. These murine feasibility procedures will allow researchers to administer an intravenous (IV) vaccine containing the top predicted immunogenic epitopes. Our ultimate goals are to directly apply and validate AutoEpiCollect’s capabilities on the next steps of vaccine development.

## 7. Conclusions

This study aimed to develop a novel, machine learning-driven vaccine design using our GUI automation software, AutoEpiCollect. Our software automatically gathers MHC Class I and II epitopes and outputs a list of top immunogenic epitopes with the use of a novel probabilistic logistic regression scoring function. With the implementation of AutoEpiCollect, pre-clinical processes of epitope collection can be significantly streamlined, allowing more resources to be allocated toward gathering clinical data for cancer vaccines. These clinical data can facilitate the advancement of precision oncology by targeting the underlying mutated genetic signatures of cancer, instead of solely the symptoms. Along with the potential to be used in combination with conventional therapies to overcome tumor resistance mechanisms, AutoEpiCollect’s epitope pan-cancer vaccines are applicable on a global scale for early disease intervention. Our automated epitope selection software is an invaluable step for the development of future precision oncology treatments and cancer prevention.

We tested AutoEpiCollect’s pan-cancer vaccine design on common cancers containing prevalent PIK3CA mutations. Previous cancer vaccines targeting the PIK3CA gene primarily target small sets of missense mutations, thus reducing the scope and efficiency of the vaccine [1,2,3,4,5]. Therefore, no clinical trials have been conducted with pan-cancer vaccines targeting the PIK3CA gene. However, AutoEpiCollect aims to strengthen the efficacy of a pan-cancer vaccine by collecting missense mutations and ranking a large set of epitopes. After inputting 49 point mutations into AutoEpiCollect, we found MHC Class I epitopes targeting 34 of these mutations and MHC Class II epitopes targeting 11 mutations. Both Class I and II epitopes were ranked based on immunogenicity, antigenicity, allergenicity, and binding affinity and were subsequently filtered based on other clinically relevant parameters. Optimized Class I epitopes resulted in 98.09% world population coverage, while optimized Class II epitopes resulted in 81.81% world population coverage. This indicates that AutoEpiCollect’s pan-cancer vaccine design was able to generate a widely applicable vaccine for Class I epitopes. Due to the HLA allele constraints of the IEDB population coverage analysis tool, further analysis must be done to validate the world coverage of Class II epitope/HLA combinations. Modeled interactions of peptides, HLA alleles, and T-cell receptors from Figure 8 were analyzed with MolProbity to demonstrate properly folded structures with minimal clashing. 

One challenge when developing AutoEpiCollect’s vaccine design process was when collecting training data for the Class II scoring function. The lack of available experimental data for 15mers epitopes significantly decreased the variety of epitopes used to train the function; as a result, the accuracy for the Class II scoring function was 56% (AUC = 0.62) when implemented on the test data. To address this issue, more experimental data and alternative in silico tools will be explored that include predictions for non-15mers epitopes. This will expand the size and diversity of the Class II scoring function training dataset, potentially leading to higher model accuracies. Epitope selection is a complex process that requires a more holistic and nuanced approach. The future architecture of AutoEpiCollect’s epitope selection algorithm will include a deep learning framework that considers a vast number of epitope characteristics before predicting top epitopes. The deep learning model will be able to handle more complex experimental training data as well, thereby representing a wider range of gold-standard epitopes for a cancer vaccine. These improvements to the selection algorithm are intended to increase confidence in using in silico methods among future clinical researchers when conducting experimental cancer vaccine trials. 

AutoEpiCollect is a comprehensive Python-based GUI application. To access the installation and user guide, please visit this project’s GitHub repository at https://github.com/mvsamudrala/AutoEpiCollect (accessed on 2 February 2024) and click on the link provided below the “About this project” section. This guide will provide detailed instructions on how to install and operate AutoEpiCollect.

## Figures and Tables

**Table 1:** 27-Allele HLA Class I and II allele references sets.

HLA Class I Allele Reference Set	HLA-A*30:01, HLA-B*40:01, HLA-A*11:01, HLA-A*68:02, HLA-A*02:06, HLA-A*31:01, HLA-A*02:03, HLA-B*44:03, HLA-A*02:01, HLA-A*01:01, HLA-A*33:01, HLA-A*23:01, HLA-B*35:01, HLA-B*08:01, HLA-B*15:01, HLA-B*07:02, HLA-B*53:01, HLA-A*26:01, HLA-A*68:01, HLA-B*57:01, HLA-A*30:02, HLA-A*24:02, HLA-B*51:01, HLA-B*44:02, HLA-A*32:01, HLA-A*03:01, HLA-B*58:01
HLA Class II Allele Reference Set	HLA-DRB4*01:01, HLA-DQA1*01:01/DQB1*05:01, HLA-DRB1*03:01, HLA-DQA1*01:02/DQB1*06:02, HLA-DRB1*13:02, HLA-DRB1*04:05, HLA-DQA1*05:01/DQB1*02:01, HLA-DRB1*15:01, HLA-DRB1*04:01, HLA-DRB3*02:02, HLA-DPA1*03:01/DPB1*04:02, HLA-DRB1*07:01, HLA-DQA1*05:01/DQB1*03:01, HLA-DRB1*12:01, HLA-DRB5*01:01, HLA-DRB1*08:02, HLA-DPA1*02:01/DPB1*01:01, HLA-DQA1*03:01/DQB1*03:02, HLA-DRB1*11:01, HLA-DRB1*09:01, HLA-DPA1*02:01/DPB1*05:01, HLA-DRB1*01:01, HLA-DPA1*02:01/DPB1*14:01, HLA-DPA1*01:03/DPB1*04:01, HLA-DRB3*01:01, HLA-DPA1*01:03/DPB1*02:01, HLA-DQA1*04:01/DQB1*04:02

**Table 2:** Binding affinity labeling scheme.

Binding Affinity (nM)	Label
BA ≤ 50	STRONG
50 < BA ≤ 500	NORMAL
500 < BA ≤ 5000	WEAK
BA > 5000	N/A

**Table 3:** Exclusion criteria determined for each parameter after ranking.

Parameter	Tool Name	Tool Link	Threshold
Instability Index	ProtParam	https://web.expasy.org/protparam/ (accessed on 2 February 2024)	<40
Half-Life	ProtParam	https://web.expasy.org/protparam/ (accessed on 2 February 2024)	>1 h
Aliphatic Index	ProtParam	https://web.expasy.org/protparam/ (accessed on 2 February 2024)	N/A
GRAVY Score	ProtParam	https://web.expasy.org/protparam/ (accessed on 2 February 2024)	N/A
Isoelectric Point	ProtParam	https://web.expasy.org/protparam/ (accessed on 2 February 2024)	N/A
Toxicity	ToxinPred	https://webs.iiitd.edu.in/raghava/toxinpred/multi_submit.php (accessed on 2 February 2024)	Non-Toxin
IFN-γ release	IFNepitope	https://webs.iiitd.edu.in/raghava/ifnepitope/predict.php (accessed on 2 February 2024)	Positive

**Table 4:** Unaccounted HLA alleles in population coverage analysis.

MHC Class II Alleles	HLA-DQA1*05:01/DQB1*02:01, HLA-DRB5*01:01, HLA-DRB3*01:01, HLA-DQA1*04:01/DQB1*04:02, HLA-DQA1*01:02/DQB1*06:02, HLA-DPA1*03:01/DPB1*04:02, HLA-DPA1*02:01/DPB1*01:01, HLA-DPA1*02:01/DPB1*14:01, HLA-DRB3*02:02, HLA-DPA1*02:01/DPB1*05:01, HLA-DQA*03:01/DQB1*03:02, HLA-DQA1*01:01/DQB1*05:01, HLA-DEB4*01:01, HLA-DPA1*01:03/DPB1*02:01, HLA-DPA1*01:03/DPB1*04:01, HLA-DQA1*05:01/HLA-DQB1*03:01

**Table 5:** Cancer types associated with all PIK3CA point mutations used in study.

Cancer	Point Mutations
Colorectal Adenocarcinoma	R38H, R88Q, G106V, C420R, E453Q, E542K, E545K, R1023Q, M1043I, H1047R
Meningioma	R108H, E110K, Y165H, N345K, I391M, E453K, E545K, G914R, H1047R
Breast Cancer	E81K, K111E, G118D, N345K, S405P, C420R, E453K, E542K, E542V, E545A, E545G, E545K, Q546E, Q546K, Q546R, E726K, H1047L, H1047R, M1043I, N1044K, G1049R
Endometrial Cancer	E542K, E542Q, E545K, E545G, G1007R, Y1021H, Y1021C, A1035V, M1043I, H1047Y, H1047R, G1050D, T1052K, H1065L
Glioblastoma Multiforme	R88E, R88Q, P298T, R310C, V344G, E453K, E542K, E545A, E545K, Y1021C, Y1021N, T1025N, T1031G, M1043I, N1044S, H1047Y, G1049S

**Table 6:** Number of point mutations containing immunogenic epitopes.

Cancer	Mutations with Class I Predicted Immunogenic Epitopes	Mutations with Class II Predicted Immunogenic Epitopes	Total Number of Mutations Analyzed	Mutations with Predicted Immunogenic Class I and II Epitopes
Colorectal Adenocarcinoma	R38H, E542K, R1023Q, M1043I, H1047R,	E453Q, M1043I, H1047R	10	M1043I, H1047R
Breast Carcinoma	E542K, M1043I, H1047R, E453K, E81K, G118D, S405P, E542V, E545A, E545G, Q546K, E726K, H1047L, N1044K, G1049R	E453K, M1043I, H1047R, H1047L, N1044K, G1049R	21	E453K, M1043I, H1047R, H1047L, N1044K, G1049R
Endometrial Carcinoma	E542K, M1043I, H1047R, E545G, E542Q, Y1021C, H1047Y, G1050D, T1052K, H1065L	M1043I, H1047R, H1047Y, G1050D, T1052K	14	M1043I, H1047R, H1047Y, G1050D, T1052K
Glioblastoma Multiforme	E542K, M1043I, E453K, E545A, Y1021C, H1047Y, P298T, R310C, V344G, T1025N, T1031G, N1044S, G1049S	E453K, M1043I, H1047Y, G1049S	17	E453K, M1043I, H1047Y, G1049S
Meningioma	H1047R, E110K, N345K, I391M, E453K, G914R	E453K, H1047R	9	E453K, H1047R

**Table 7:** World population coverage results for Class I and II epitopes.

Calculation	MHC Class I Filtered	MHC Class I Optimized	MHC Class II Filtered	MHC Class II Optimized
Population Coverage	98.09%	98.09%	81.81%	81.81%
Average Epitope Hit	27.36	3.82	11.87	1.01
PC90	7.96	2.02	4.4	0.55

## Data Availability

All the data generated in this study are displayed within the manuscript, and supplied in the Supplementary Materials. The AutoEpiCollect is a comprehensive Python-based GUI application code can be accessed through GitHub link: https://github.com/mvsamudrala/AutoEpiCollect (accessed on 2 February 2024).

## Supplementary Materials

The following supporting information can be downloaded at: https://www.mdpi.com/article/10.3390/bioengineering11040322/s1, Supplementary Tables S1–S4. These tables include the training data for the linear regression and logistic regression MHC Class I and II scoring functions. Supplementary Tables S5–S9. These tables include the unfiltered and unranked MHC Class I epitope/HLA allele data for each cancer type. Supplementary Tables S10–S14. These tables include the unfiltered and unranked MHC Class II epitope/HLA allele data for each cancer type. Supplementary Tables S15 and S16. These tables include the final lists of top filtered and ranked MHC Class I and II epitope/HLA allele data by point mutation. Supplementary Tables S17 and S18. These tables include the MHC Class I and II epitopes used to find the optimized population coverage. Supplementary Table S19 and S20. These tables include the filtered and optimized population coverages for MHC Class I epitope/HLA combinations. Supplementary Table S21 and S22. These tables include the filtered and optimized population coverages for MHC Class II epitope/HLA combinations. Supplementary Table S23–S25. These tables include the MolProbity results for the 3D models of predicted immunogenic peptide–MHC complexes bound to TCR complexes. Supplementary Table S26. This table includes the TCR α/β sequences of the TCR complexes bound to predicted immunogenic peptide–MHC complexes. Supplementary Figure S1. Population coverage graphs for the filtered MHC Class I final epitope data. Supplementary Figure S2. Population coverage graphs for the optimized MHC Class I final epitope data. Supplementary Figure S3. Population coverage graphs for the filtered MHC Class II final epitope data. Supplementary Figure S4. Population coverage graphs for the optimized MHC Class II final epitope data. Supplementary Files 3D coordinate PDB Files of 3D pMHC-TCR Models.
