# Supplementary material for: AutoEpiCollect, a Novel Machine Learning-Based GUI Software for Vaccine Design: Application to Pan-Cancer Vaccine Design Targeting PIK3CA Neoantigens
Source: Bioengineering (Basel). 2024 Mar 27;11(4):322. doi: 10.3390/bioengineering11040322 (PMC11048108; doi:10.3390/bioengineering11040322)
Supplement: Supplementary file 1 [file bioengineering-11-00322-s001.zip › Supplementary Data/Supplementary Figures.zip!/Supplementary Figures/Supplementary Figure S1.pdf]

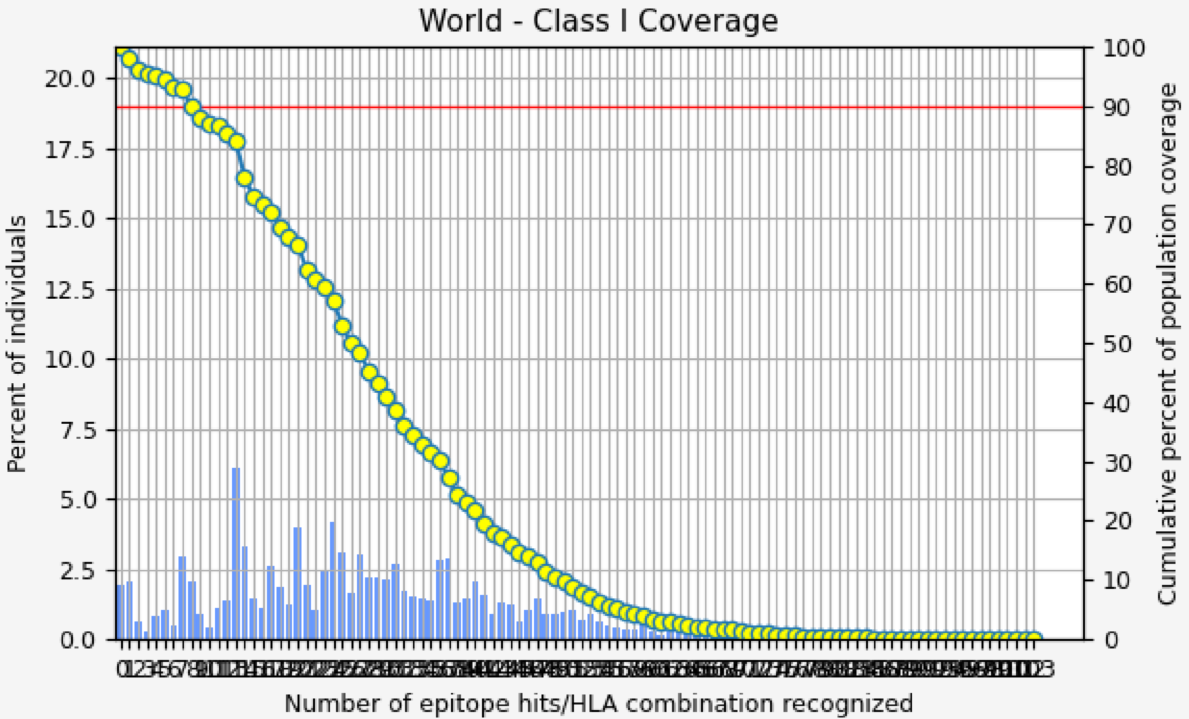

World - Class I Coverage
Percent of individuals
Cumulative percent of population coverage
Number of epitope hits/HLA combination recognized
0.0
2.5
5.0
7.5
10.0
12.5
15.0
17.5
20.0
0
10
20
30
40
50
60
70
80
90
100

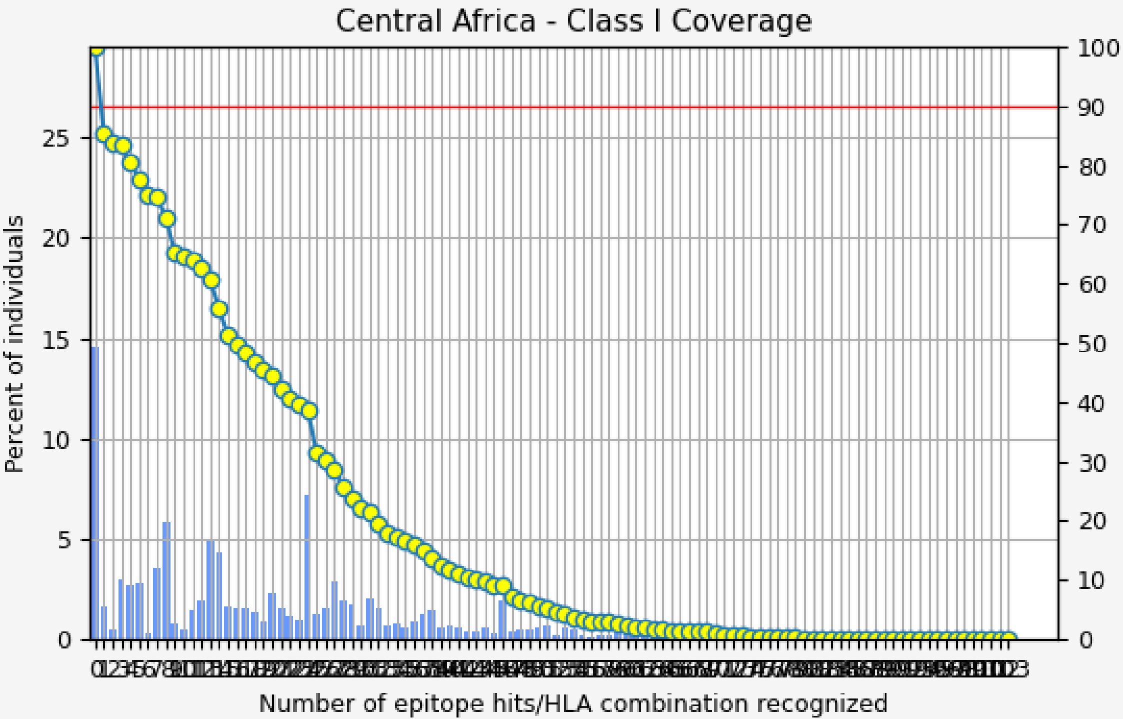

Central Africa - Class I Coverage
Percent of individuals
Cumulative percent of population coverage
Number of epitope hits/HLA combination recognized
0
5
10
15
20
25
0
10
20
30
40
50
60
70
80
90
100

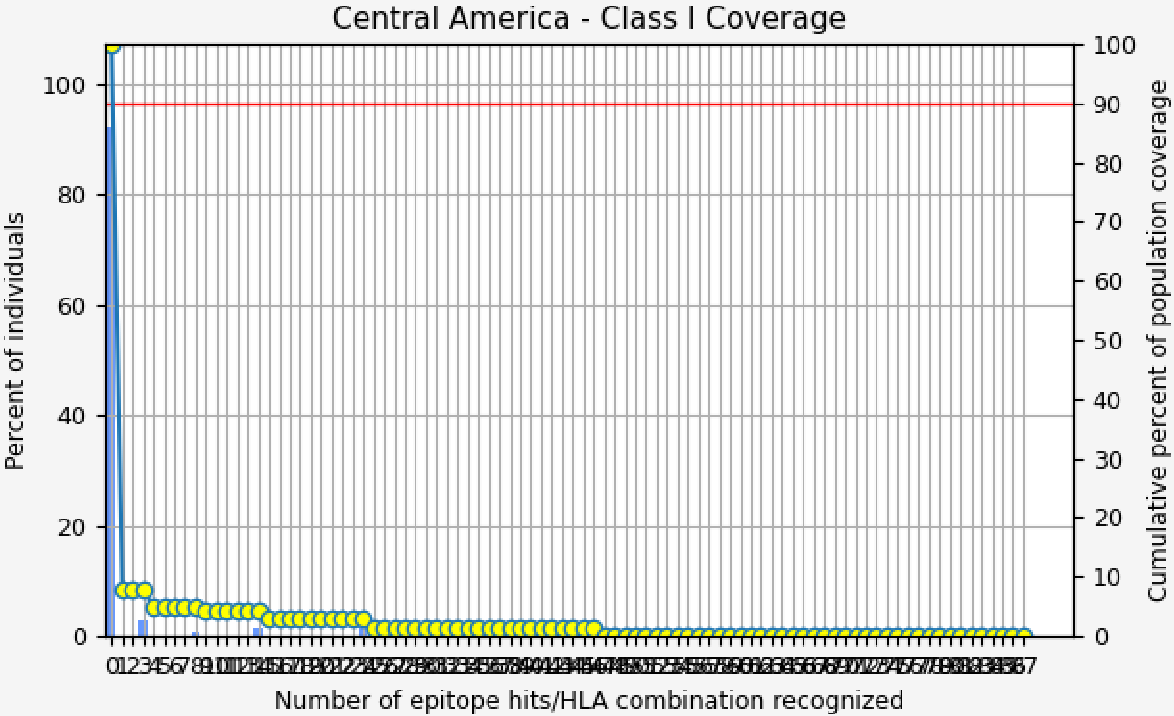
Central America - Class I Coverage
Percent of individuals
Cumulative percent of population coverage
Number of epitope hits/HLA combination recognized
0
20
40
60
80
100
0
10
20
30
40
50
60
70
80
90
100

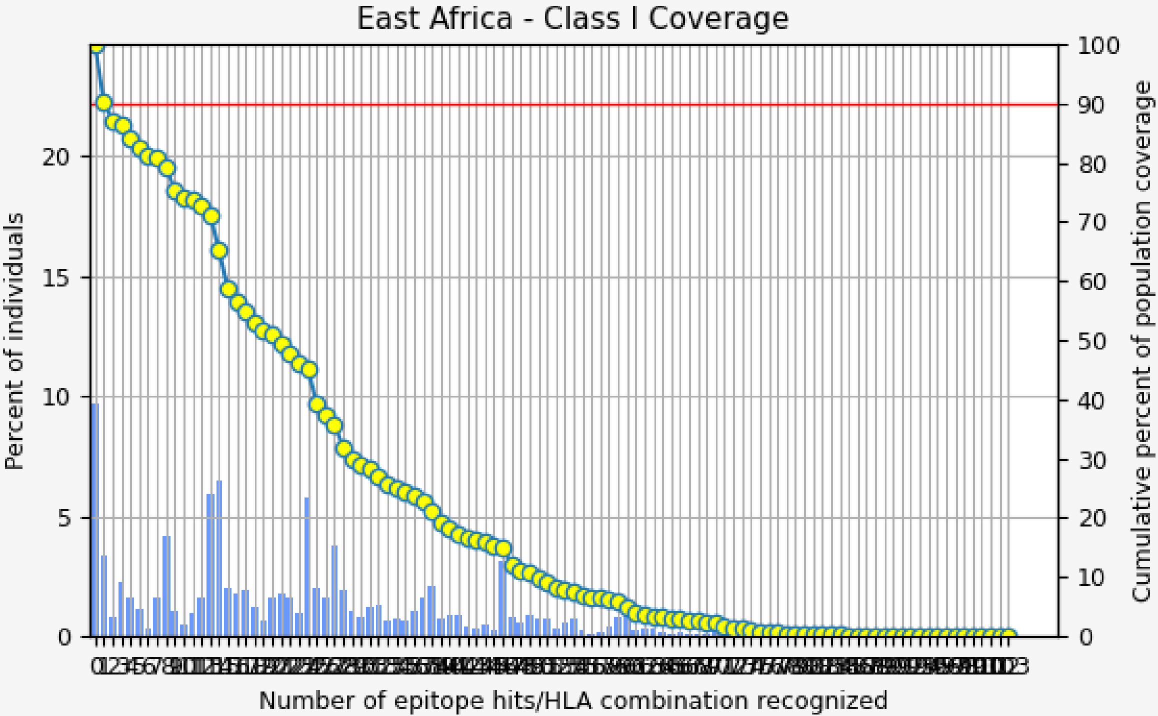

East Africa - Class I Coverage
Percent of individuals
Cumulative percent of population coverage
Number of epitope hits/HLA combination recognized
0
5
10
15
20
0
10
20
30
40
50
60
70
80
90
100

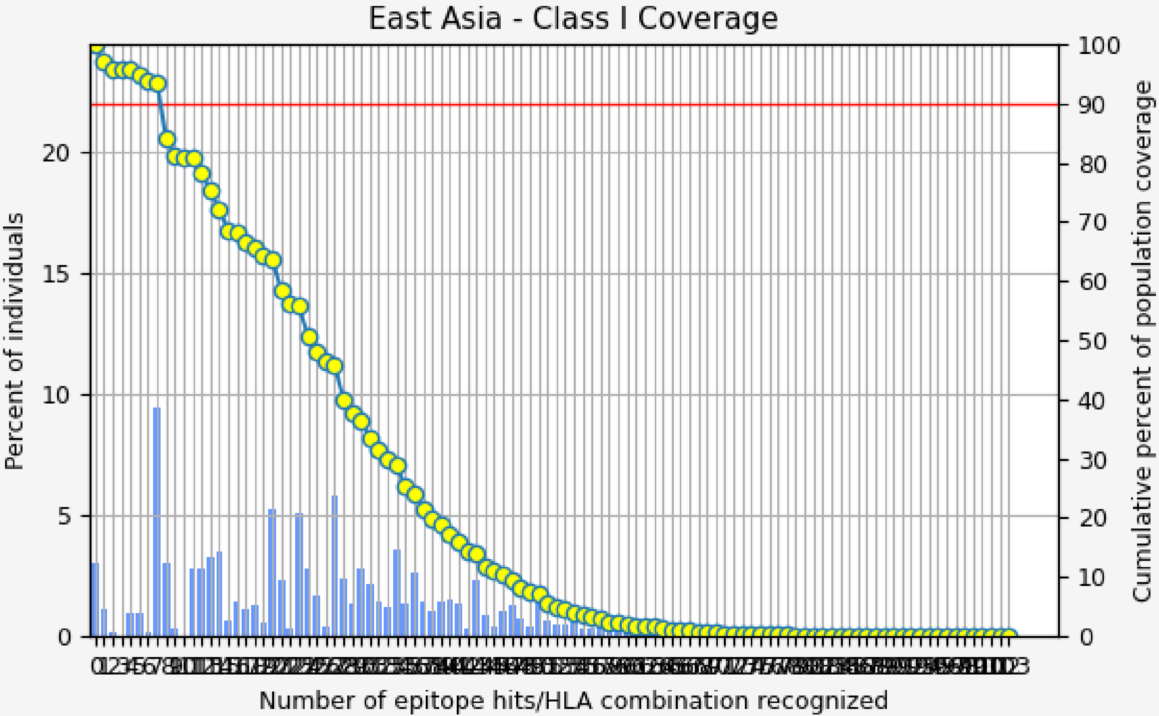
East Asia - Class I Coverage
Percent of individuals
Cumulative percent of population coverage
Number of epitope hits/HLA combination recognized
0
5
10
15
20
0
10
20
30
40
50
60
70
80
90
100

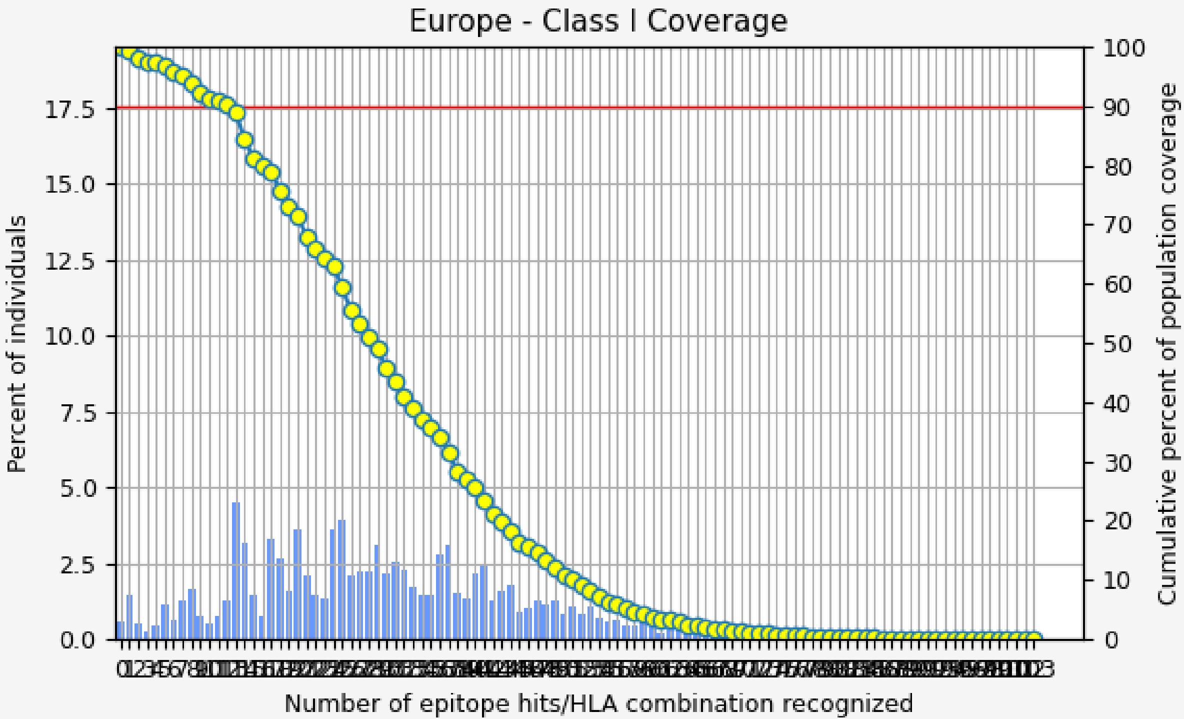

Europe - Class I Coverage
Percent of individuals
0.0
2.5
5.0
7.5
10.0
12.5
15.0
17.5
Cumulative percent of population coverage
0
10
20
30
40
50
60
70
80
90
100
Number of epitope hits/HLA combination recognized

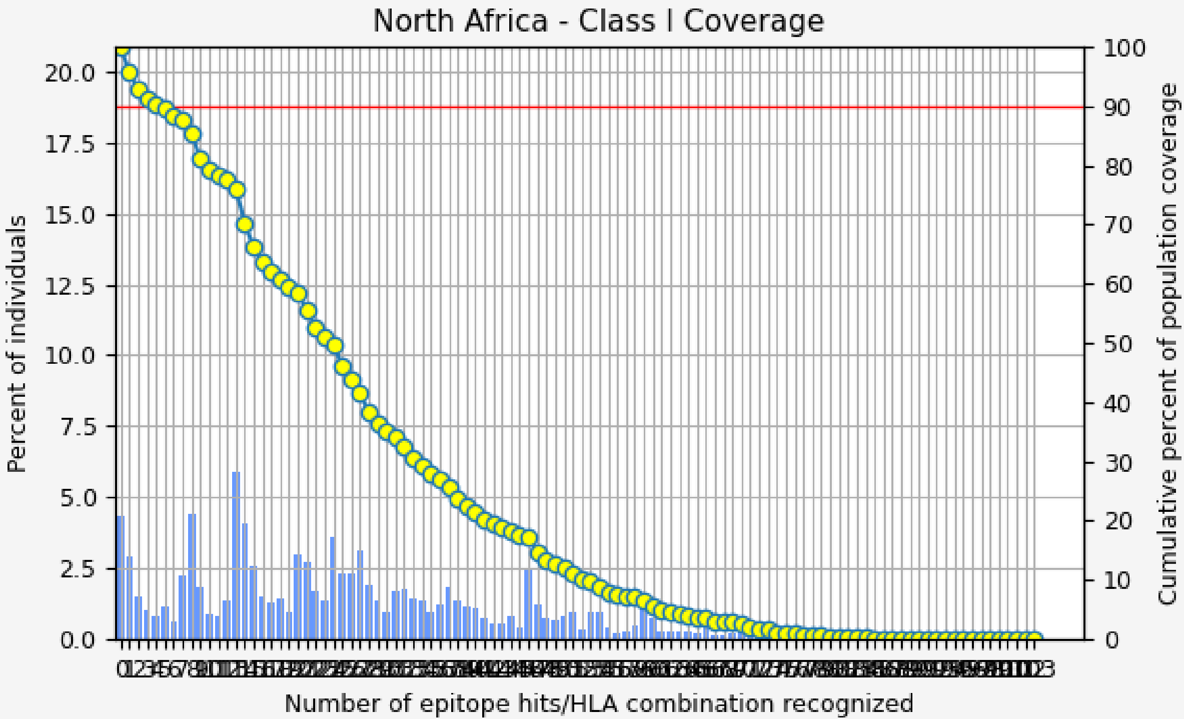

North Africa - Class I Coverage
Percent of individuals
Cumulative percent of population coverage
Number of epitope hits/HLA combination recognized
0.0
2.5
5.0
7.5
10.0
12.5
15.0
17.5
20.0
0
10
20
30
40
50
60
70
80
90
100

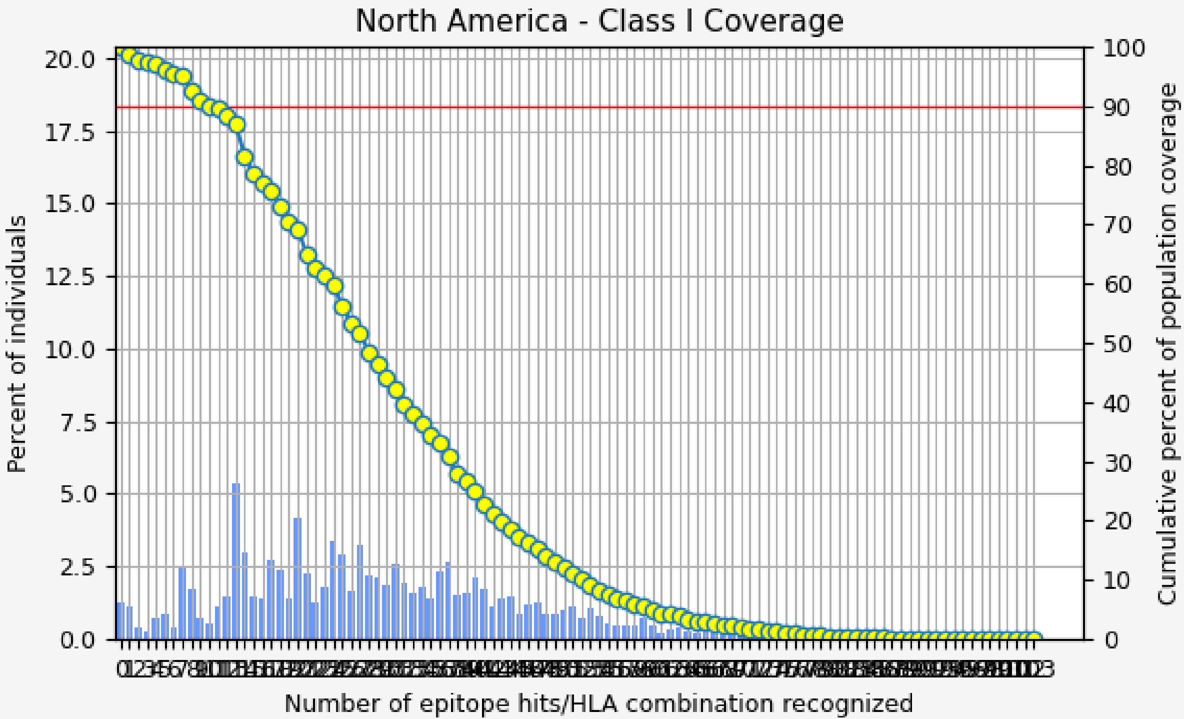
North America - Class I Coverage
Percent of individuals
20.0
17.5
15.0
12.5
10.0
7.5
5.0
2.5
0.0
Cumulative percent of population coverage
100
90
80
70
60
50
40
30
20
10
0
Number of epitope hits/HLA combination recognized

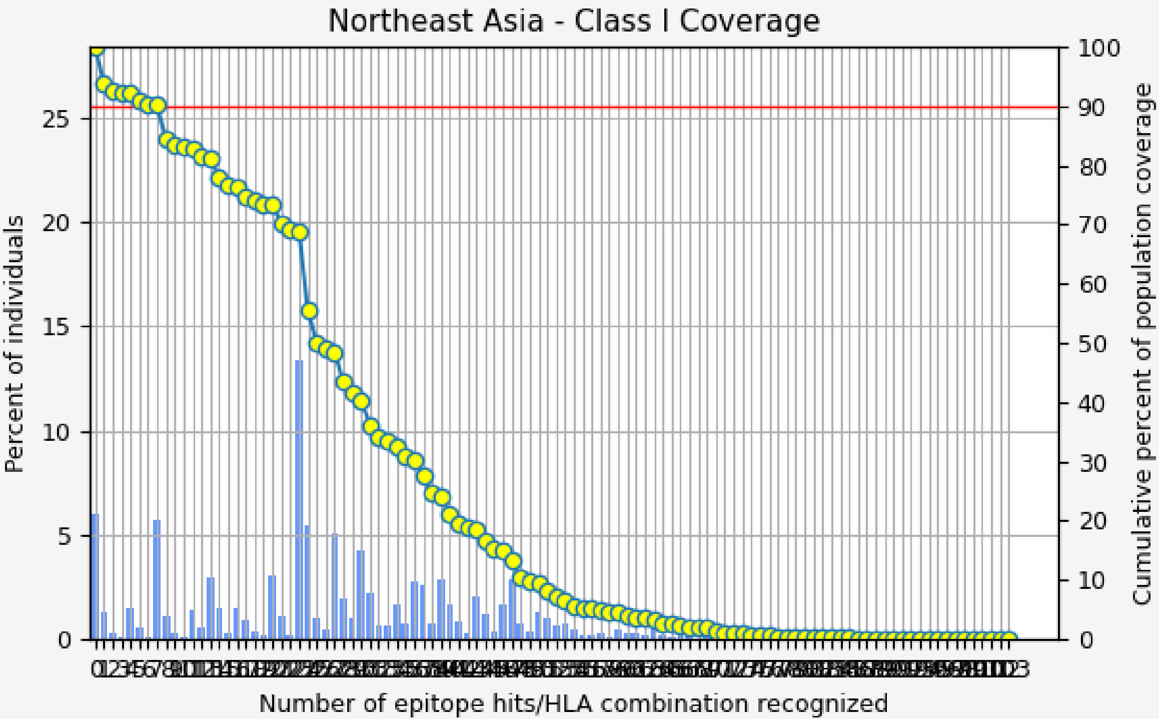

Northeast Asia - Class I Coverage
Percent of individuals
Cumulative percent of population coverage
Number of epitope hits/HLA combination recognized
0
5
10
15
20
25
0
10
20
30
40
50
60
70
80
90
100

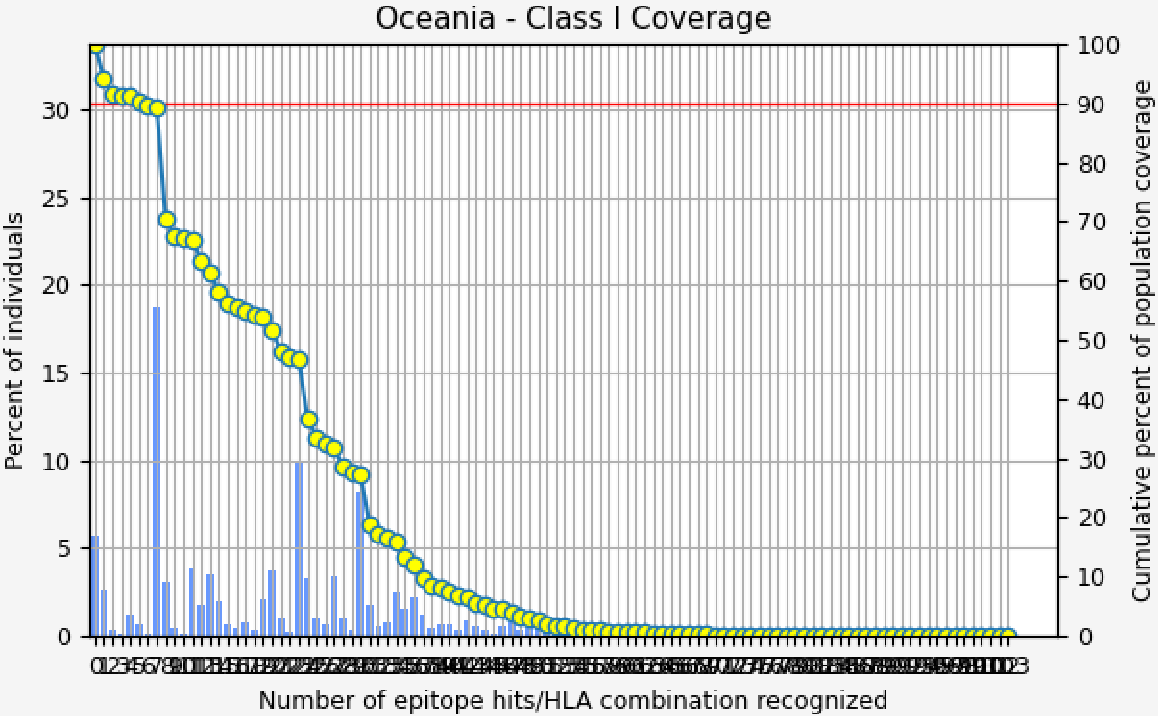

Oceania - Class I Coverage
Percent of individuals
Cumulative percent of population coverage
Number of epitope hits/HLA combination recognized
0
5
10
15
20
25
30
0
10
20
30
40
50
60
70
80
90
100

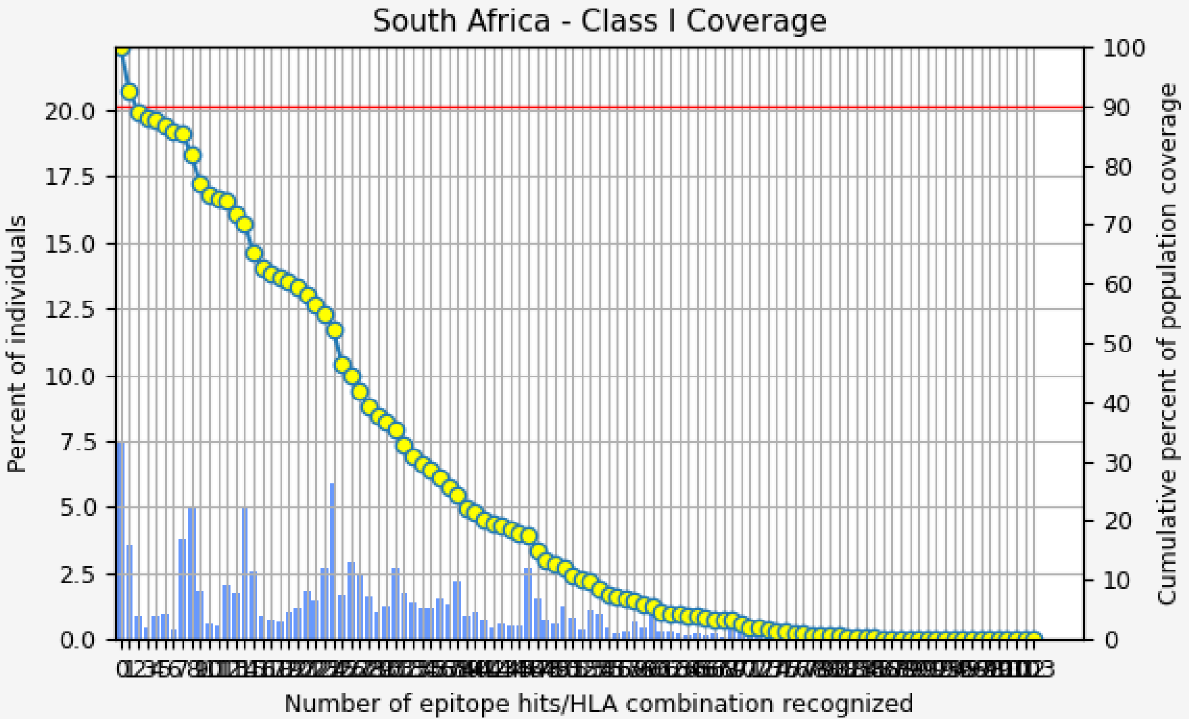

South Africa - Class I Coverage
Percent of individuals
Cumulative percent of population coverage
Number of epitope hits/HLA combination recognized
0.0
2.5
5.0
7.5
10.0
12.5
15.0
17.5
20.0
0
10
20
30
40
50
60
70
80
90
100

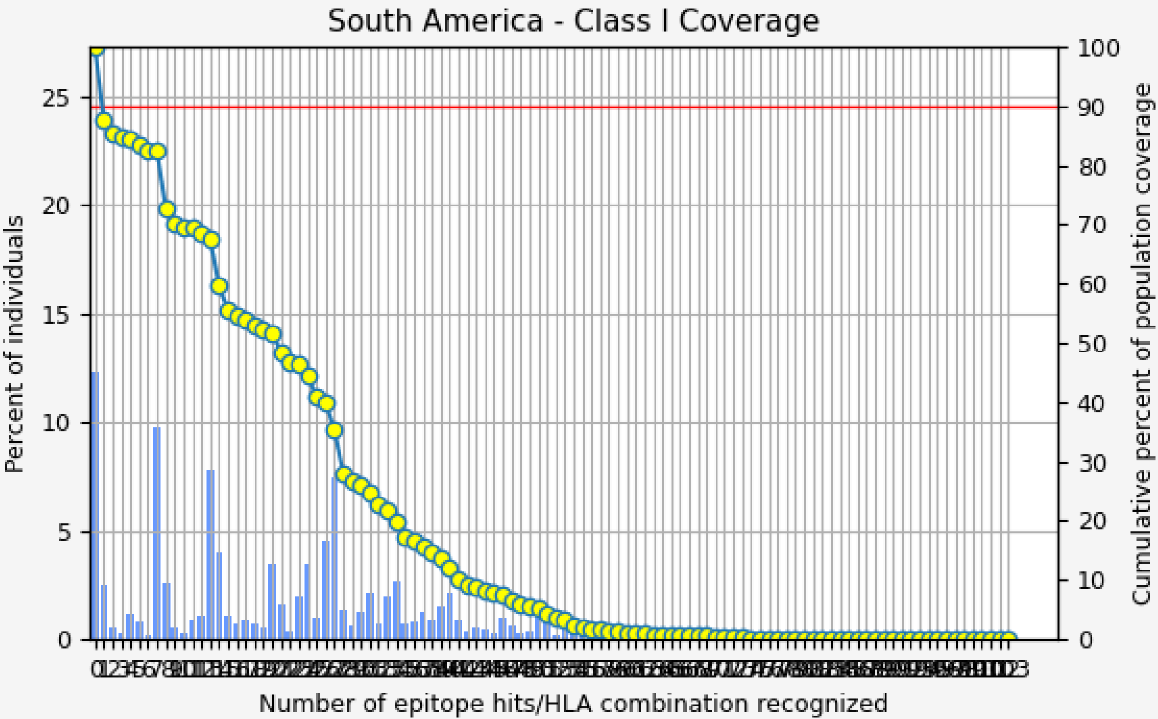
South America - Class I Coverage
Percent of individuals
Cumulative percent of population coverage
Number of epitope hits/HLA combination recognized
0
5
10
15
20
25
0
10
20
30
40
50
60
70
80
90
100

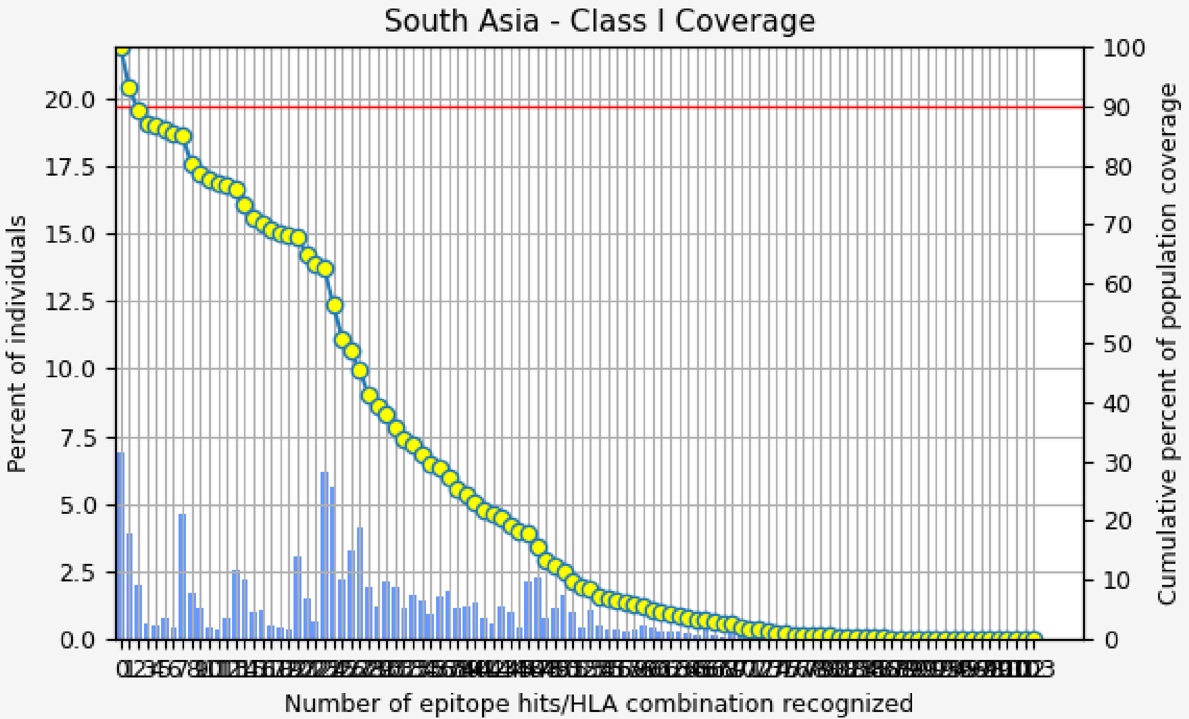
South Asia - Class I Coverage
Percent of individuals
Cumulative percent of population coverage
Number of epitope hits/HLA combination recognized
0.0
2.5
5.0
7.5
10.0
12.5
15.0
17.5
20.0
0
10
20
30
40
50
60
70
80
90
100

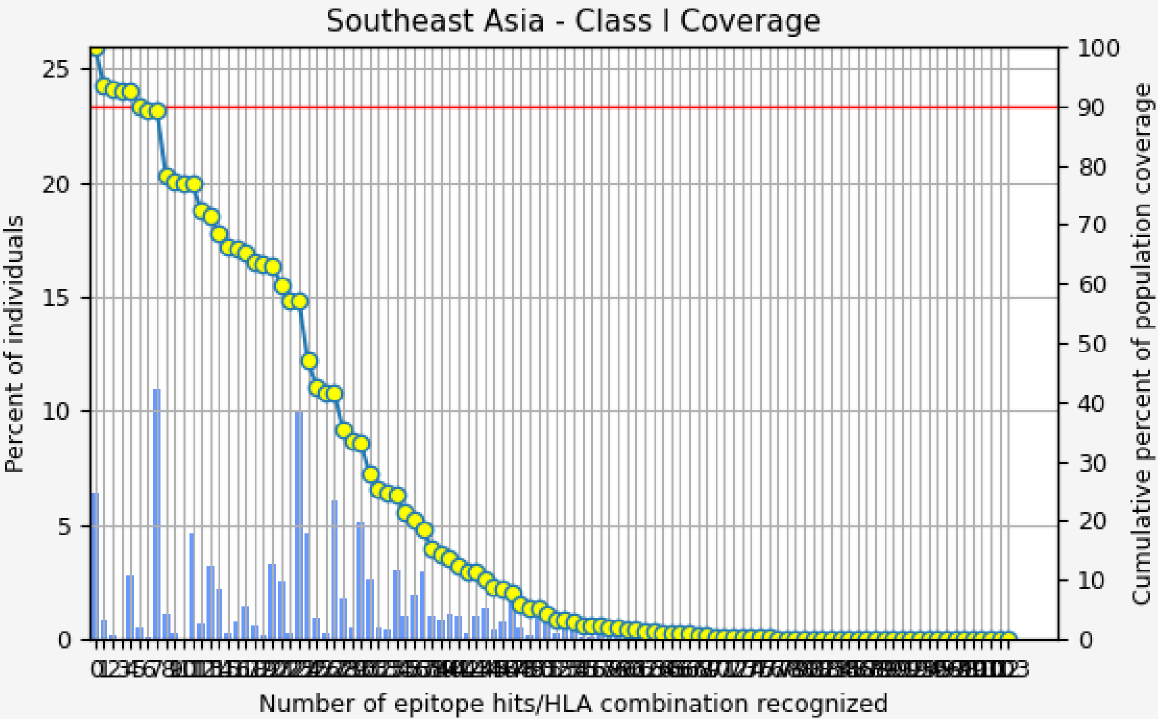

Southeast Asia - Class I Coverage
Percent of individuals
Cumulative percent of population coverage
Number of epitope hits/HLA combination recognized

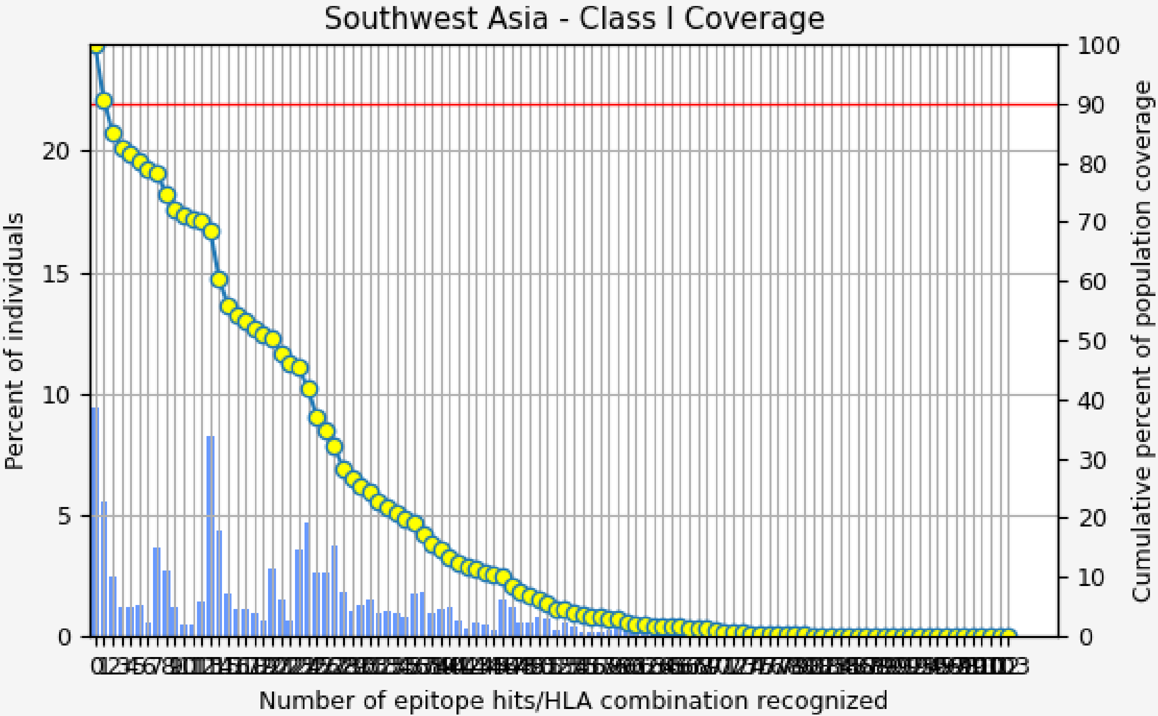

Southwest Asia - Class I Coverage
Percent of individuals
Cumulative percent of population coverage
Number of epitope hits/HLA combination recognized
0
5
10
15
20
0
10
20
30
40
50
60
70
80
90
100

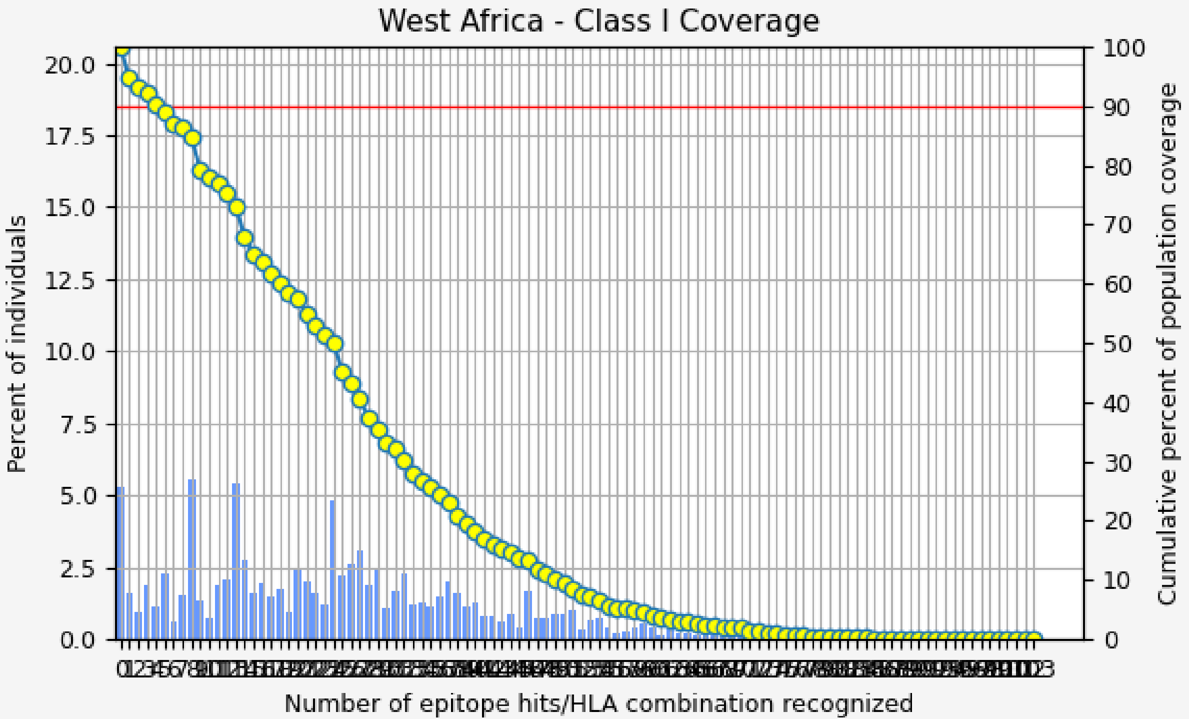

West Africa - Class I Coverage
Percent of individuals
Cumulative percent of population coverage
Number of epitope hits/HLA combination recognized
0.0
2.5
5.0
7.5
10.0
12.5
15.0
17.5
20.0
0
10
20
30
40
50
60
70
80
90
100

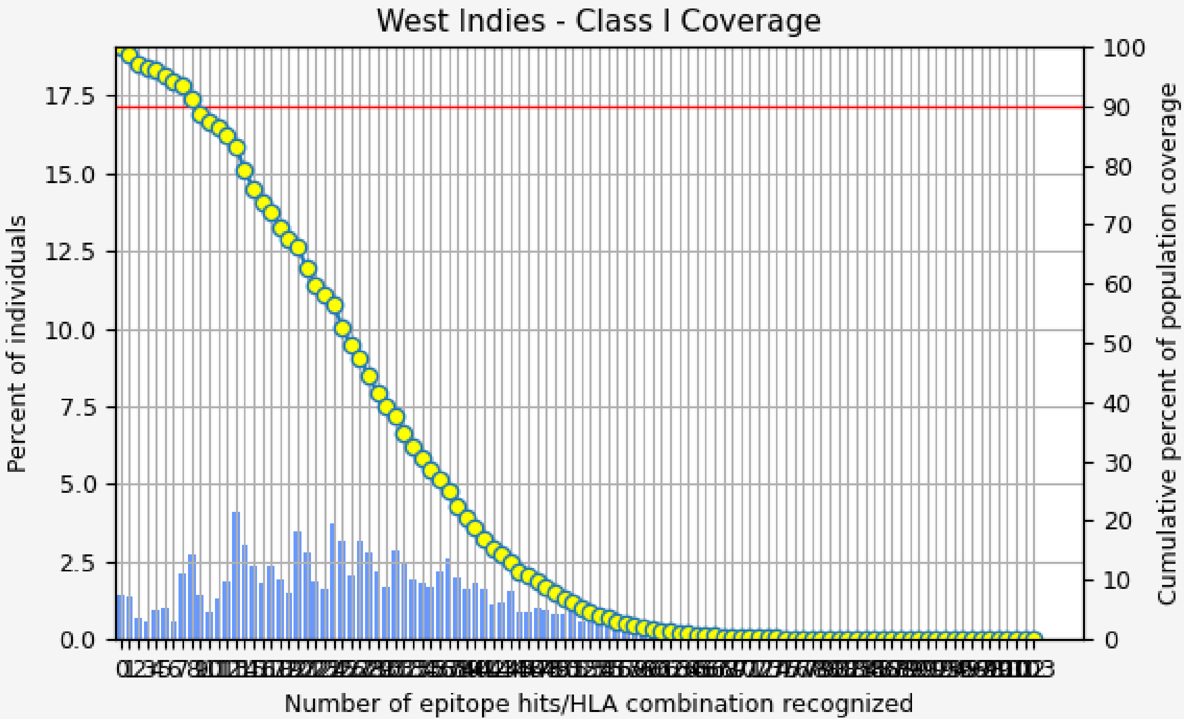
West Indies - Class I Coverage
Percent of individuals
Cumulative percent of population coverage
Number of epitope hits/HLA combination recognized
0.0
2.5
5.0
7.5
10.0
12.5
15.0
17.5
0
10
20
30
40
50
60
70
80
90
100
