# Supplementary material for: AutoEpiCollect, a Novel Machine Learning-Based GUI Software for Vaccine Design: Application to Pan-Cancer Vaccine Design Targeting PIK3CA Neoantigens
Source: Bioengineering (Basel). 2024 Mar 27;11(4):322. doi: 10.3390/bioengineering11040322 (PMC11048108; doi:10.3390/bioengineering11040322)
Supplement: Supplementary file 1 [file bioengineering-11-00322-s001.zip › Supplementary Data/Supplementary Figures.zip!/Supplementary Figures/Supplementary Figure S2.pdf]

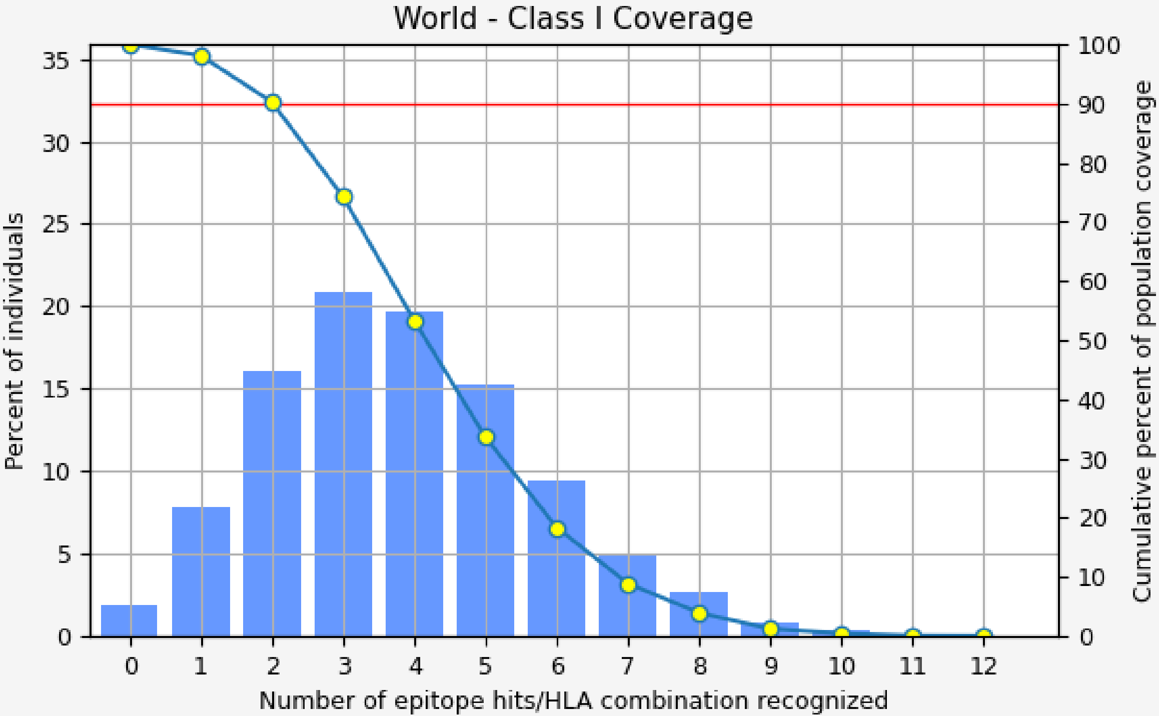
World - Class I Coverage
Percent of individuals
Cumulative percent of population coverage
Number of epitope hits/HLA combination recognized
0
5
10
15
20
25
30
35
0
10
20
30
40
50
60
70
80
90
100
0
1
2
3
4
5
6
7
8
9
10
11
12

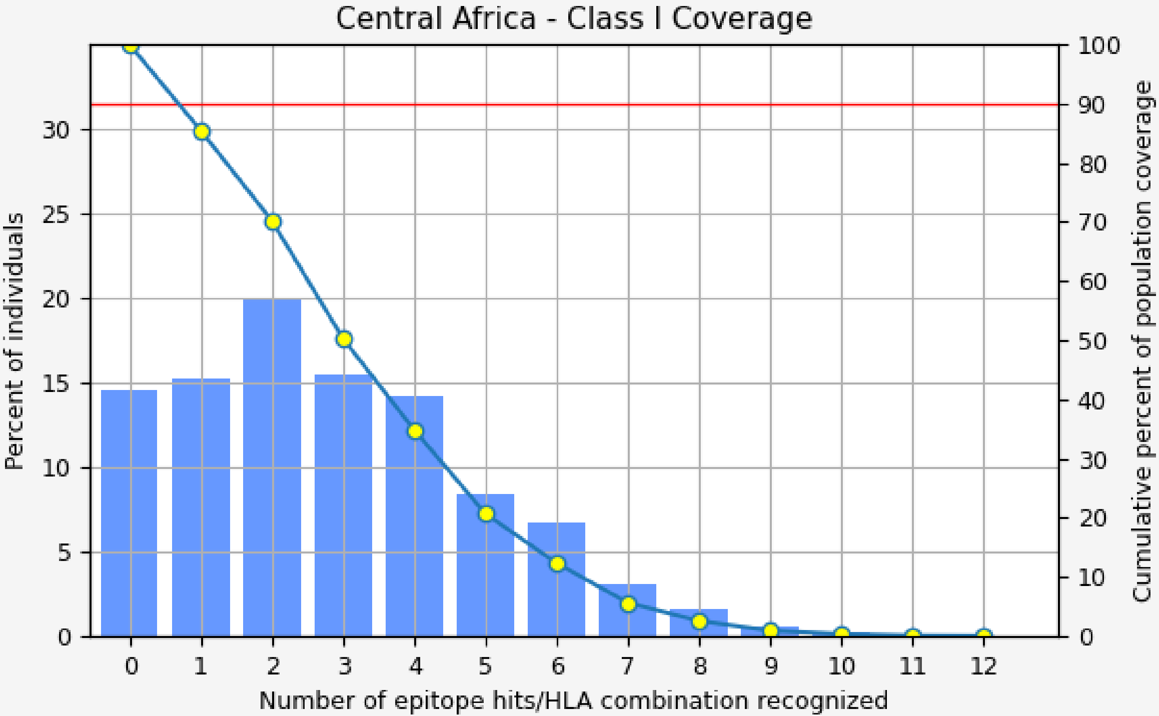
Central Africa - Class I Coverage
Percent of individuals
Cumulative percent of population coverage
Number of epitope hits/HLA combination recognized
0
5
10
15
20
25
30
100
90
80
70
60
50
40
30
20
10
0
0 1 2 3 4 5 6 7 8 9 10 11 12

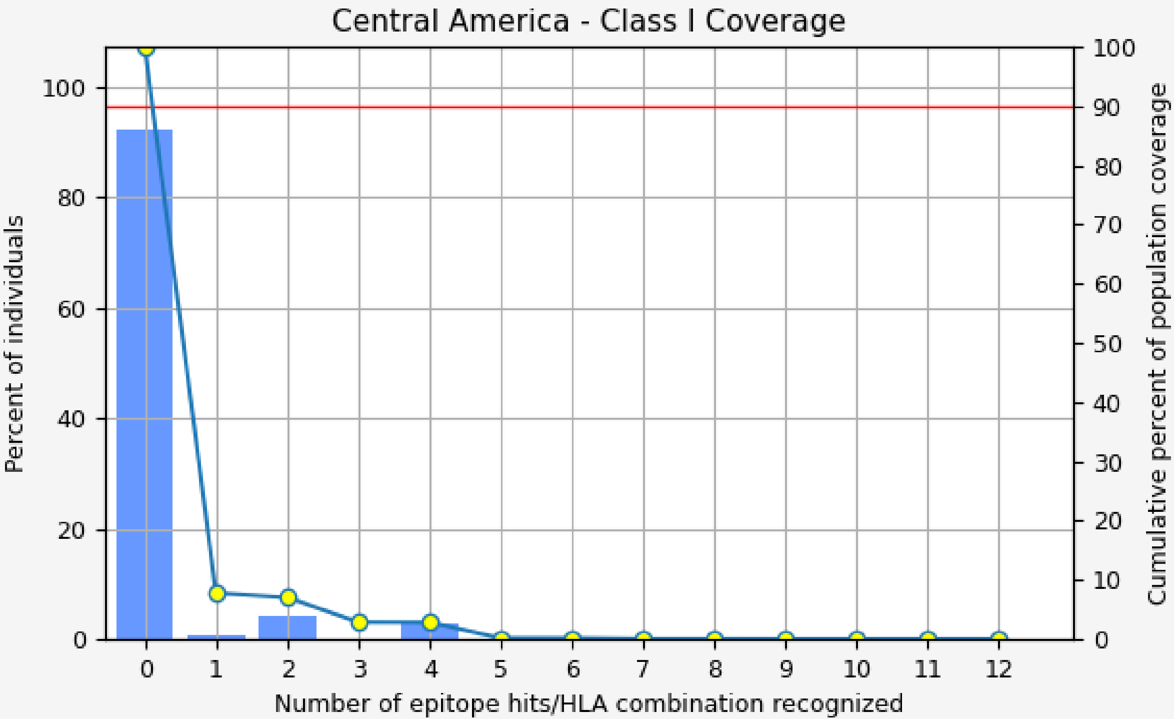
Central America - Class I Coverage
Percent of individuals
0
20
40
60
80
100
Cumulative percent of population coverage
0
10
20
30
40
50
60
70
80
90
100
0
1
2
3
4
5
6
7
8
9
10
11
12
Number of epitope hits/HLA combination recognized

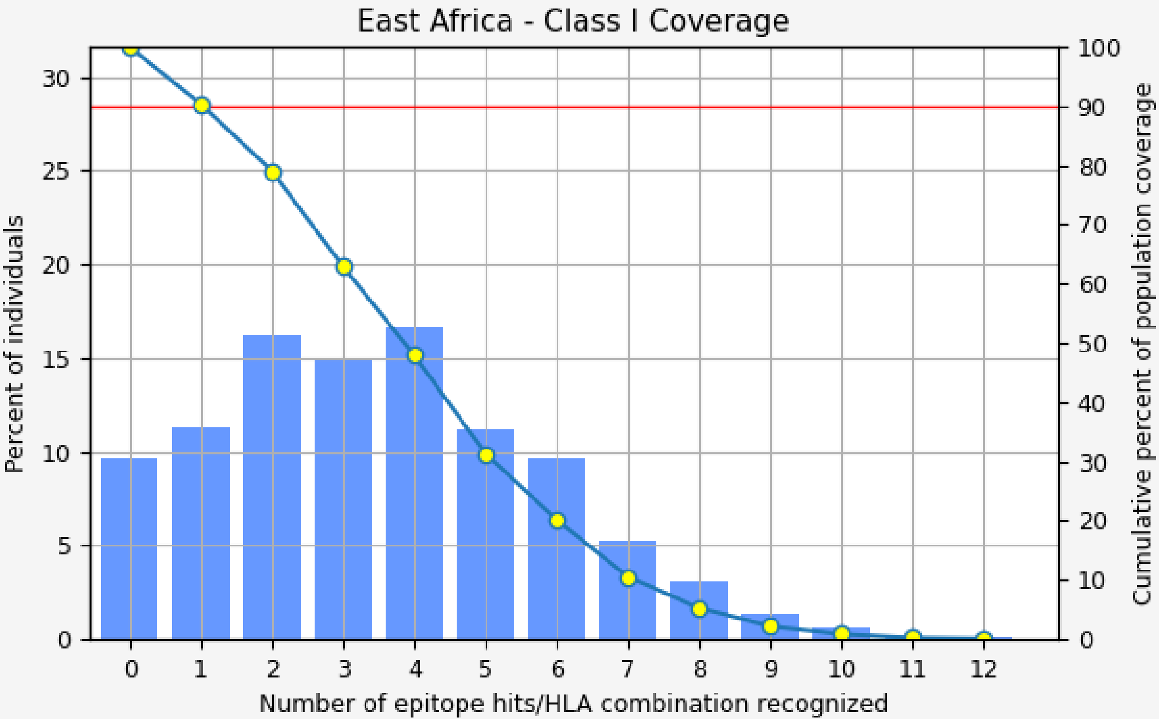
East Africa - Class I Coverage
Percent of individuals
Cumulative percent of population coverage
Number of epitope hits/HLA combination recognized
0
5
10
15
20
25
30
0
10
20
30
40
50
60
70
80
90
100
0
1
2
3
4
5
6
7
8
9
10
11
12

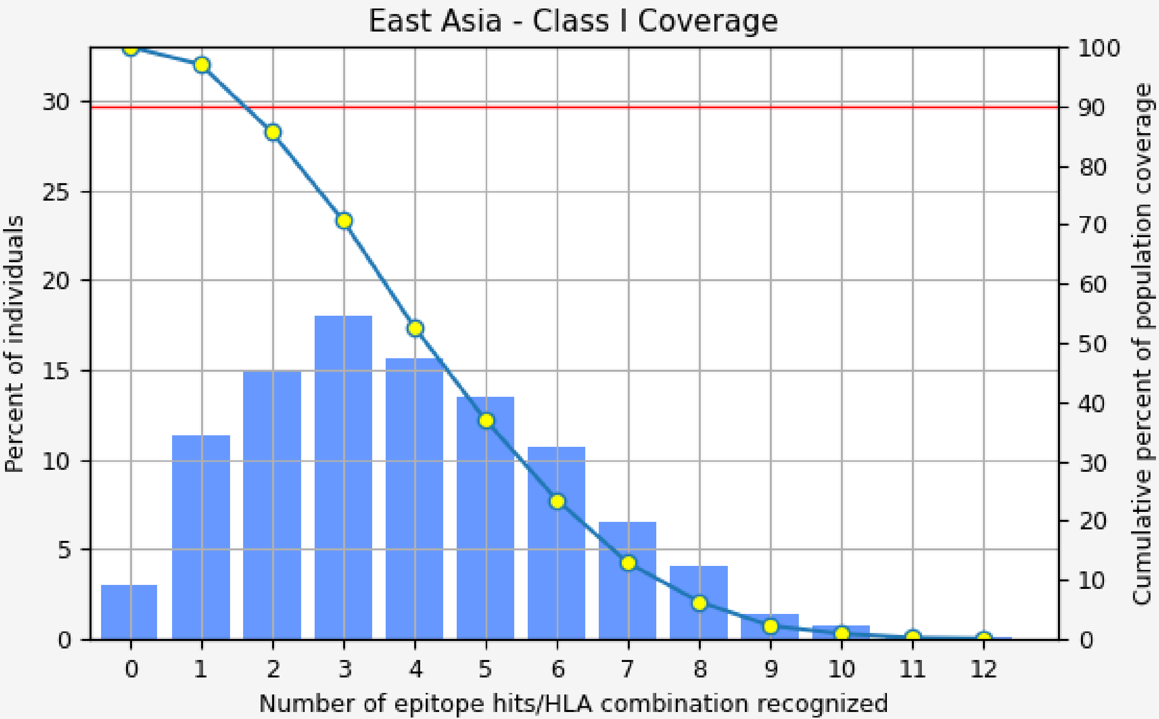

East Asia - Class I Coverage
Percent of individuals
Cumulative percent of population coverage
Number of epitope hits/HLA combination recognized
0
5
10
15
20
25
30
0
10
20
30
40
50
60
70
80
90
100
0
1
2
3
4
5
6
7
8
9
10
11
12

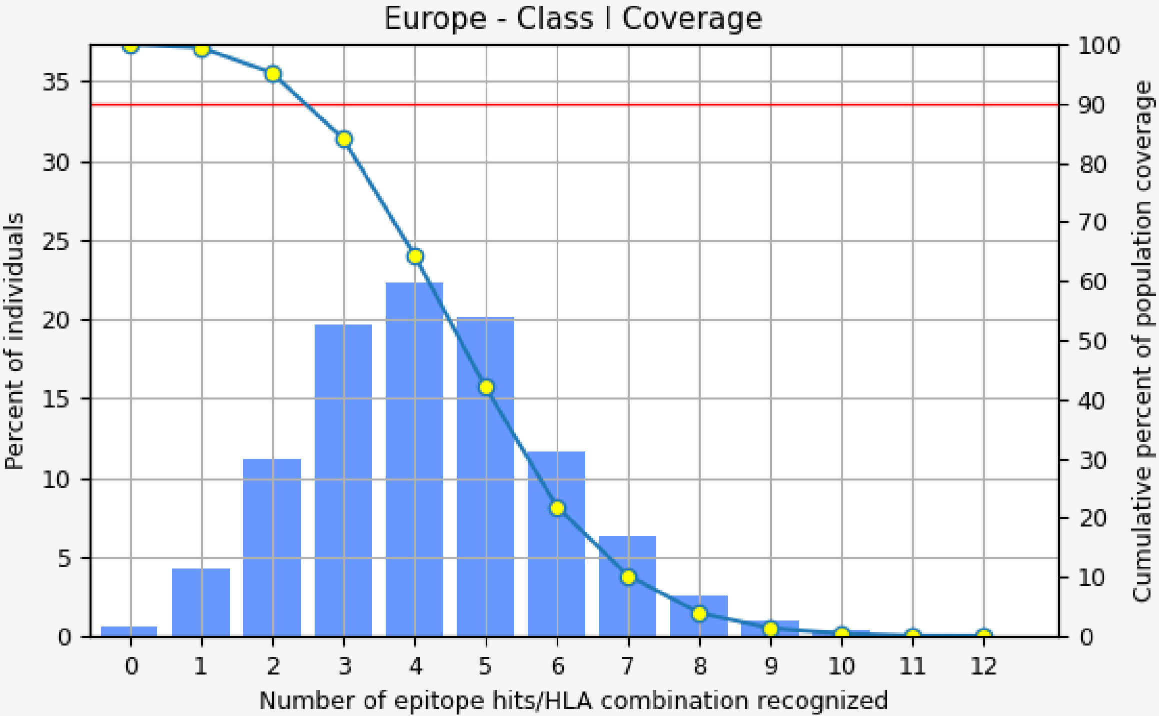

Europe - Class I Coverage
Percent of individuals
Cumulative percent of population coverage
Number of epitope hits/HLA combination recognized
0
1
2
3
4
5
6
7
8
9
10
11
12
0
5
10
15
20
25
30
35
100
90
80
70
60
50
40
30
20
10

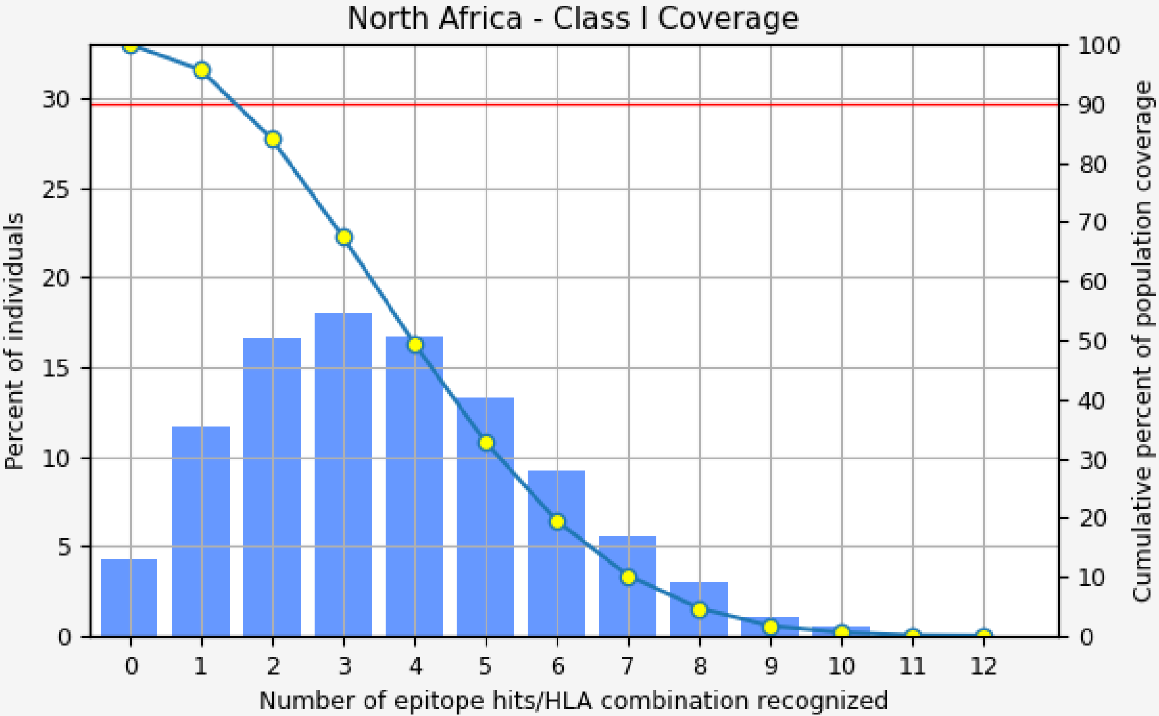

North Africa - Class I Coverage
Percent of individuals
Cumulative percent of population coverage
Number of epitope hits/HLA combination recognized
0
5
10
15
20
25
30
100
90
80
70
60
50
40
30
20
10
0
0 1 2 3 4 5 6 7 8 9 10 11 12

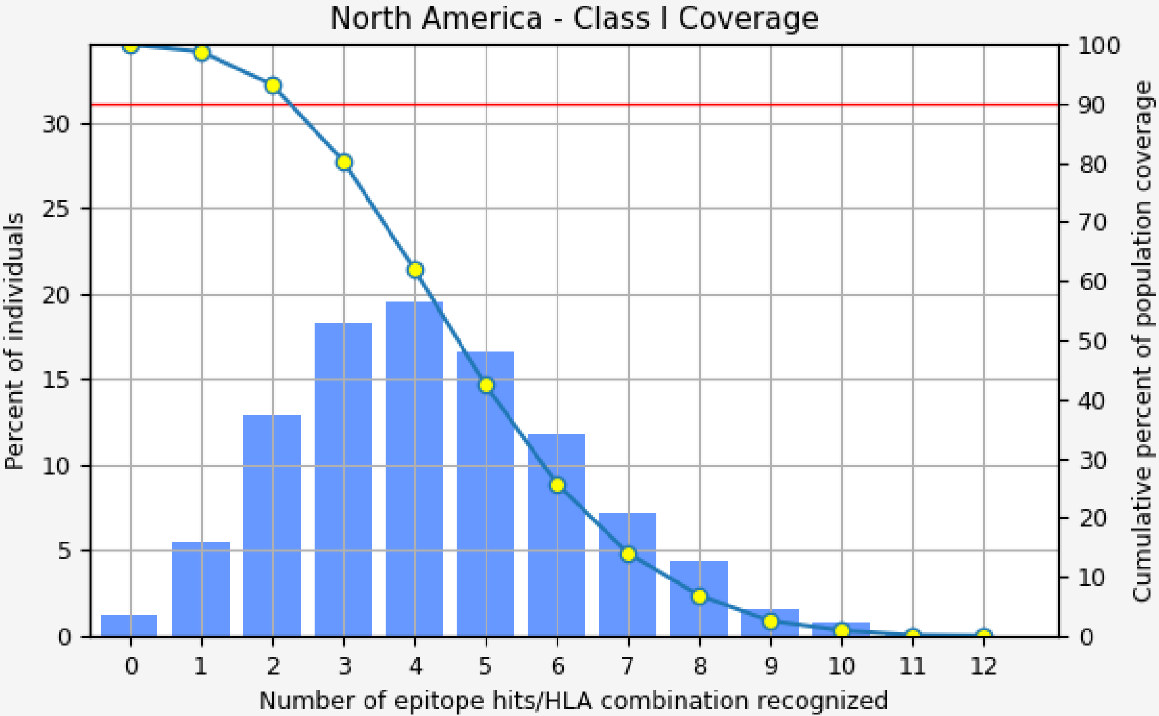

North America - Class I Coverage
Percent of individuals
Cumulative percent of population coverage
Number of epitope hits/HLA combination recognized
0
5
10
15
20
25
30
0
10
20
30
40
50
60
70
80
90
100
0
1
2
3
4
5
6
7
8
9
10
11
12

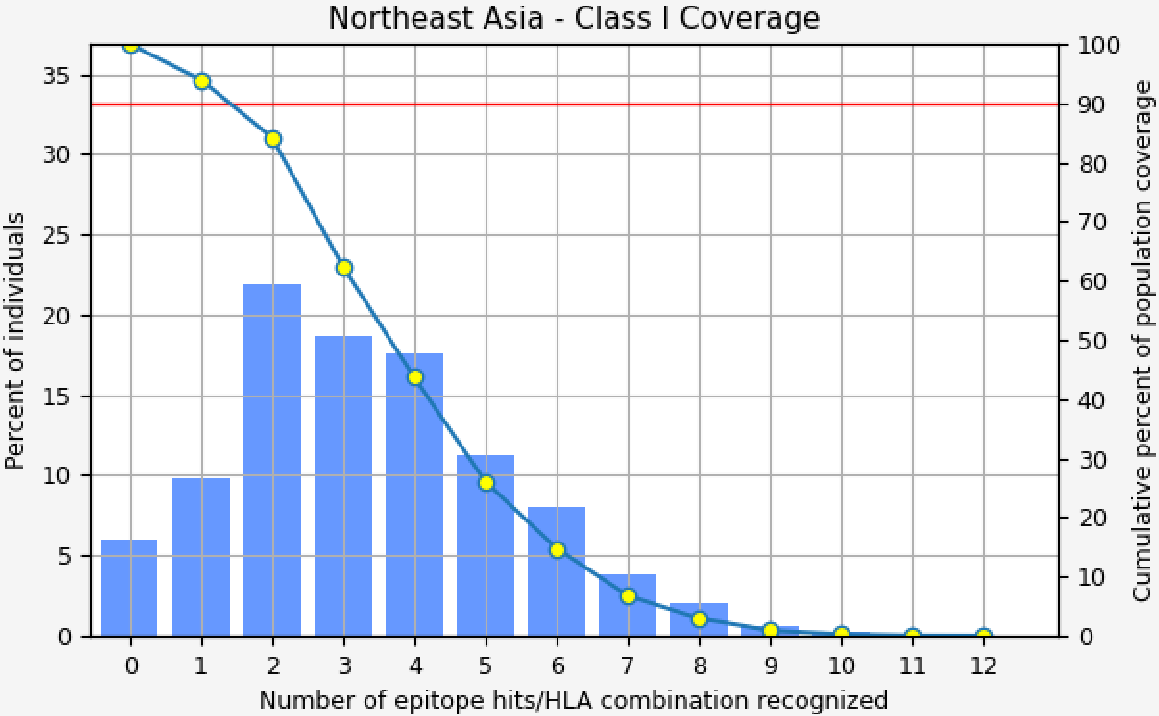
Northeast Asia - Class I Coverage
Percent of individuals
0
5
10
15
20
25
30
35
Cumulative percent of population coverage
0
10
20
30
40
50
60
70
80
90
100
0
1
2
3
4
5
6
7
8
9
10
11
12
Number of epitope hits/HLA combination recognized

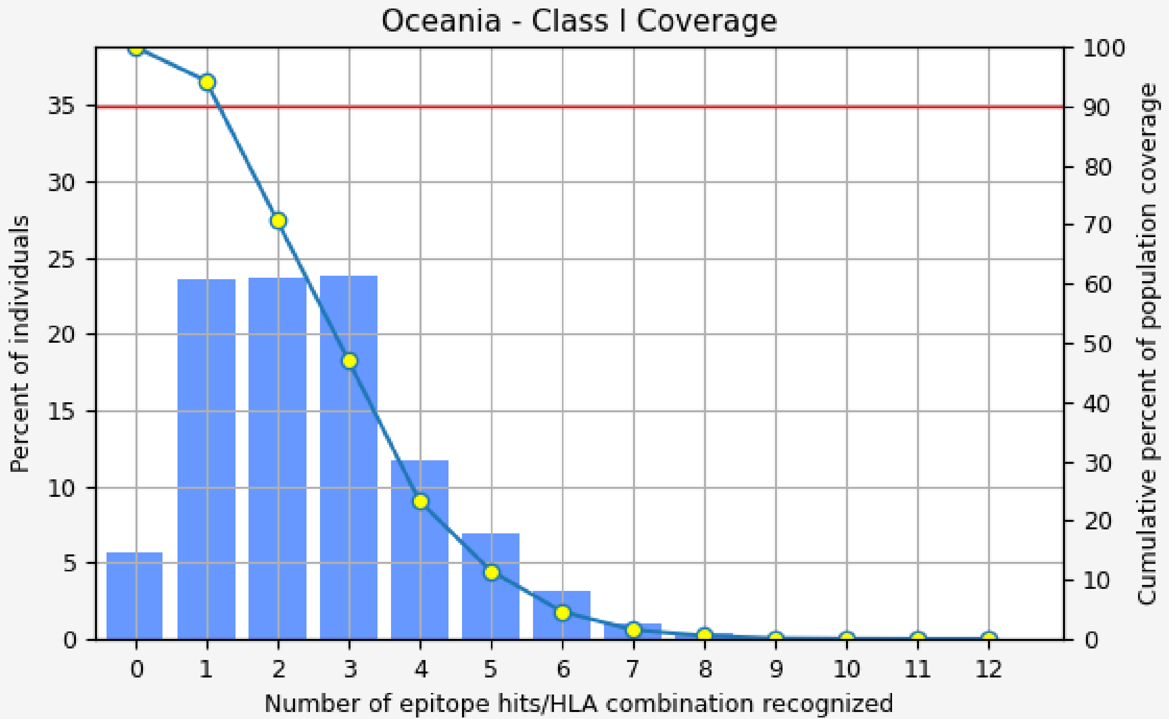

Oceania - Class I Coverage
Percent of individuals
Cumulative percent of population coverage
Number of epitope hits/HLA combination recognized
0
5
10
15
20
25
30
35
0
10
20
30
40
50
60
70
80
90
100
0
1
2
3
4
5
6
7
8
9
10
11
12

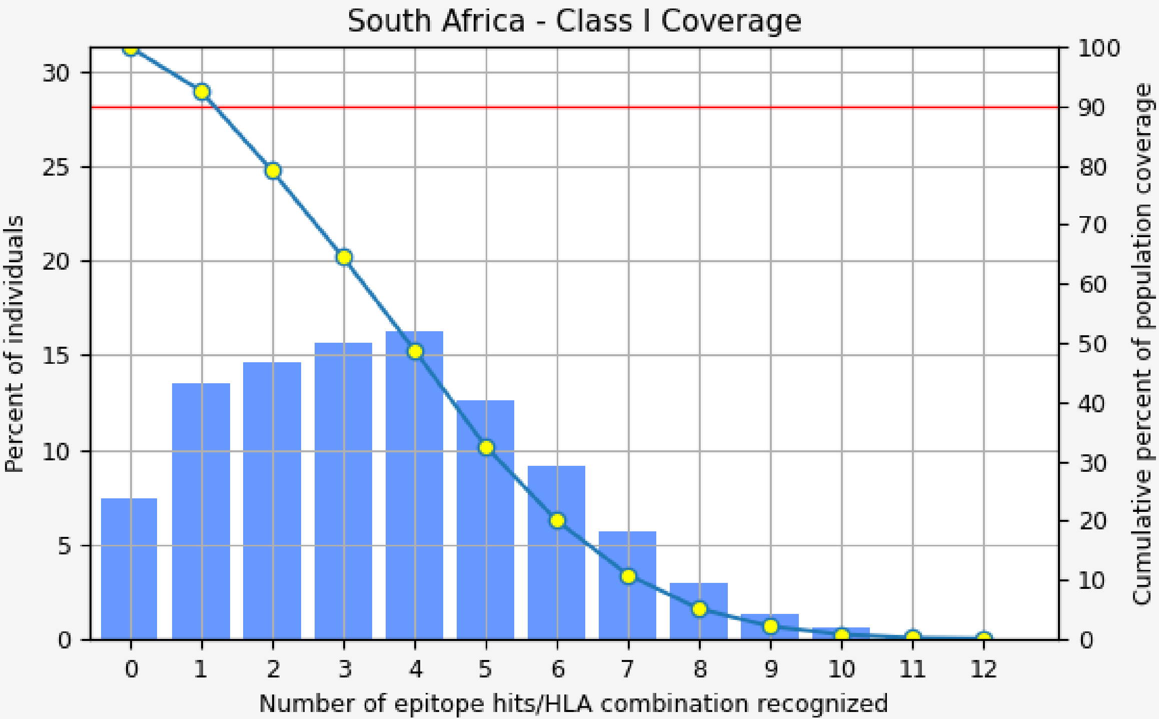

South Africa - Class I Coverage
Percent of individuals
Cumulative percent of population coverage
Number of epitope hits/HLA combination recognized
0
5
10
15
20
25
30
100
90
80
70
60
50
40
30
20
10
0
0 1 2 3 4 5 6 7 8 9 10 11 12

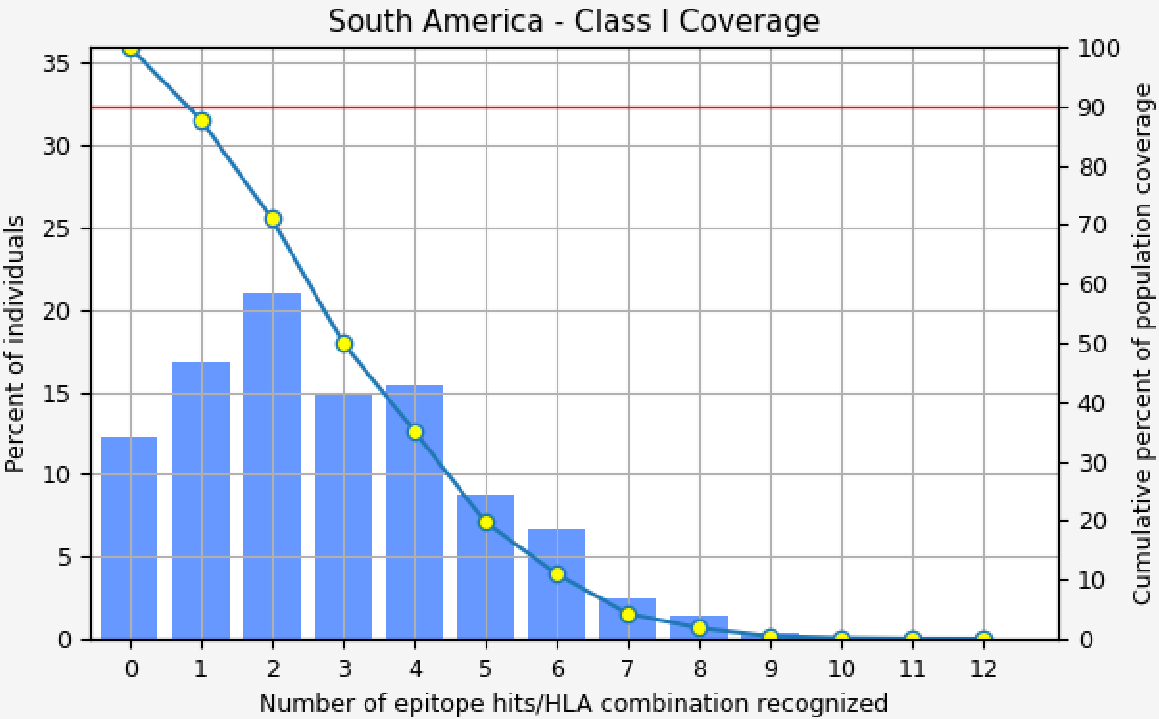

South America - Class I Coverage
Percent of individuals
Cumulative percent of population coverage
Number of epitope hits/HLA combination recognized
0
5
10
15
20
25
30
35
0
10
20
30
40
50
60
70
80
90
100
0
1
2
3
4
5
6
7
8
9
10
11
12

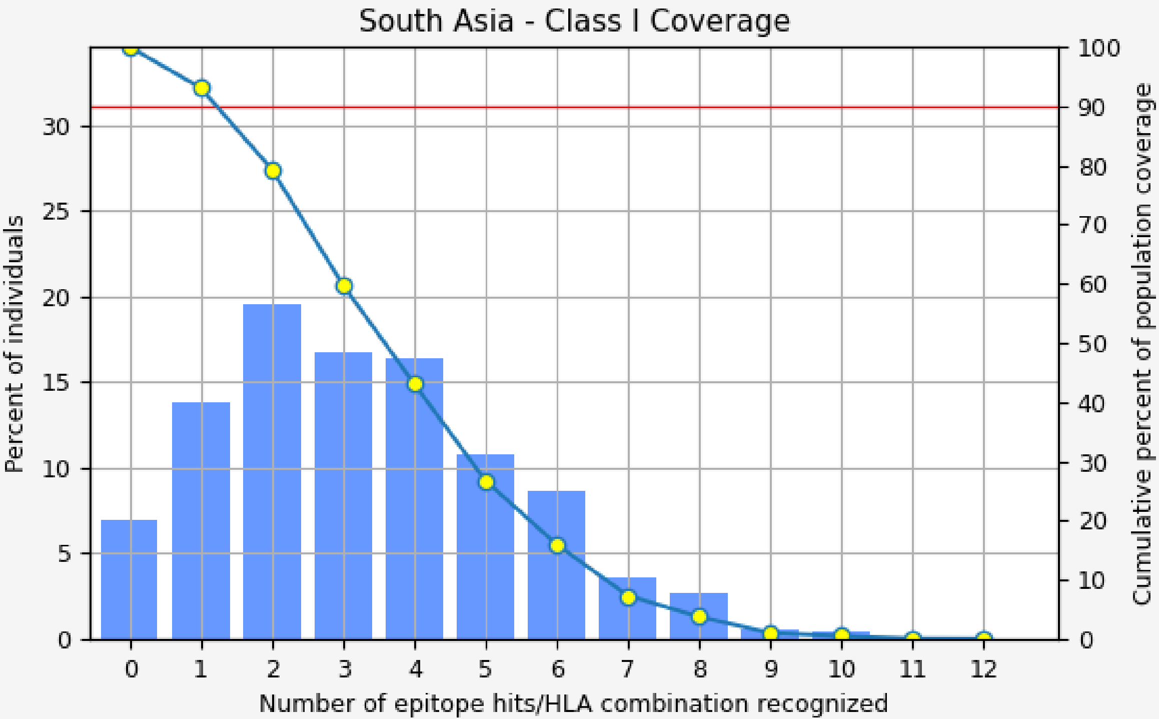

South Asia - Class I Coverage
Percent of individuals
Cumulative percent of population coverage
Number of epitope hits/HLA combination recognized
0
5
10
15
20
25
30
0
10
20
30
40
50
60
70
80
90
100
0
1
2
3
4
5
6
7
8
9
10
11
12

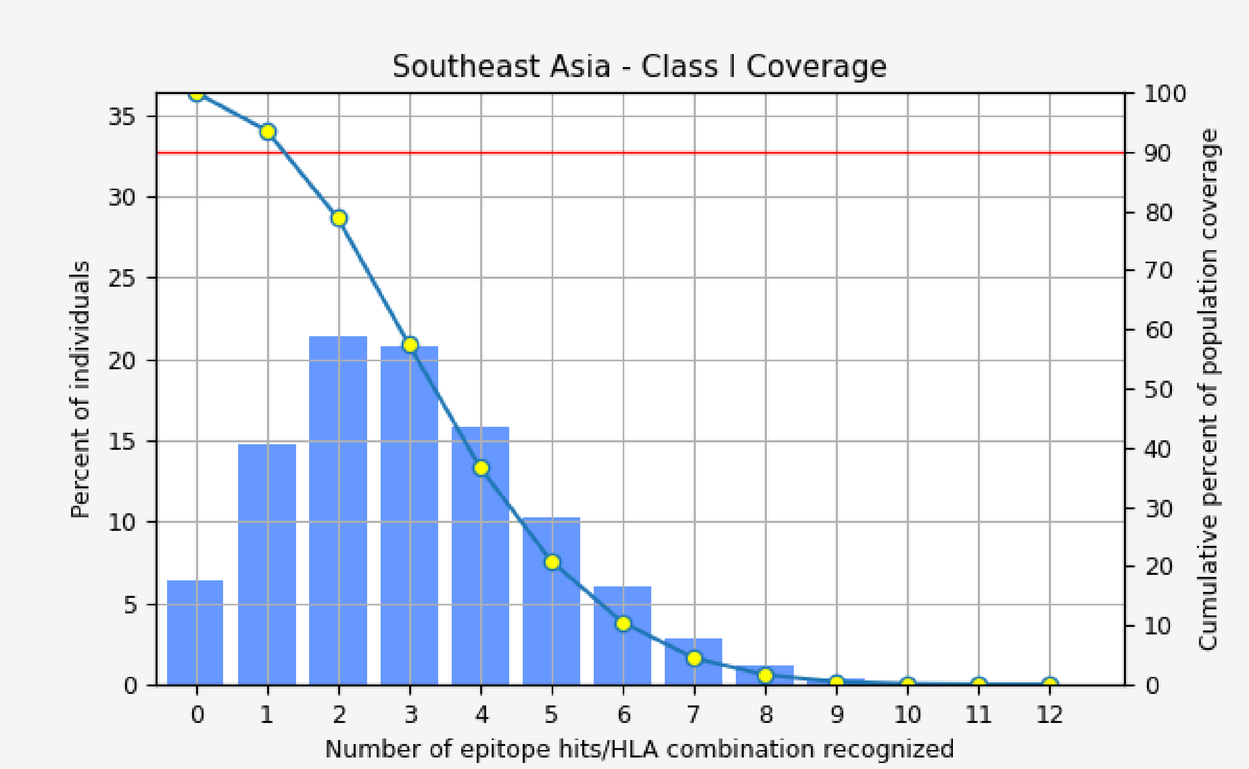

Southeast Asia - Class I Coverage
Percent of individuals
Cumulative percent of population coverage
Number of epitope hits/HLA combination recognized
0
5
10
15
20
25
30
35
0
10
20
30
40
50
60
70
80
90
100
0 1 2 3 4 5 6 7 8 9 10 11 12

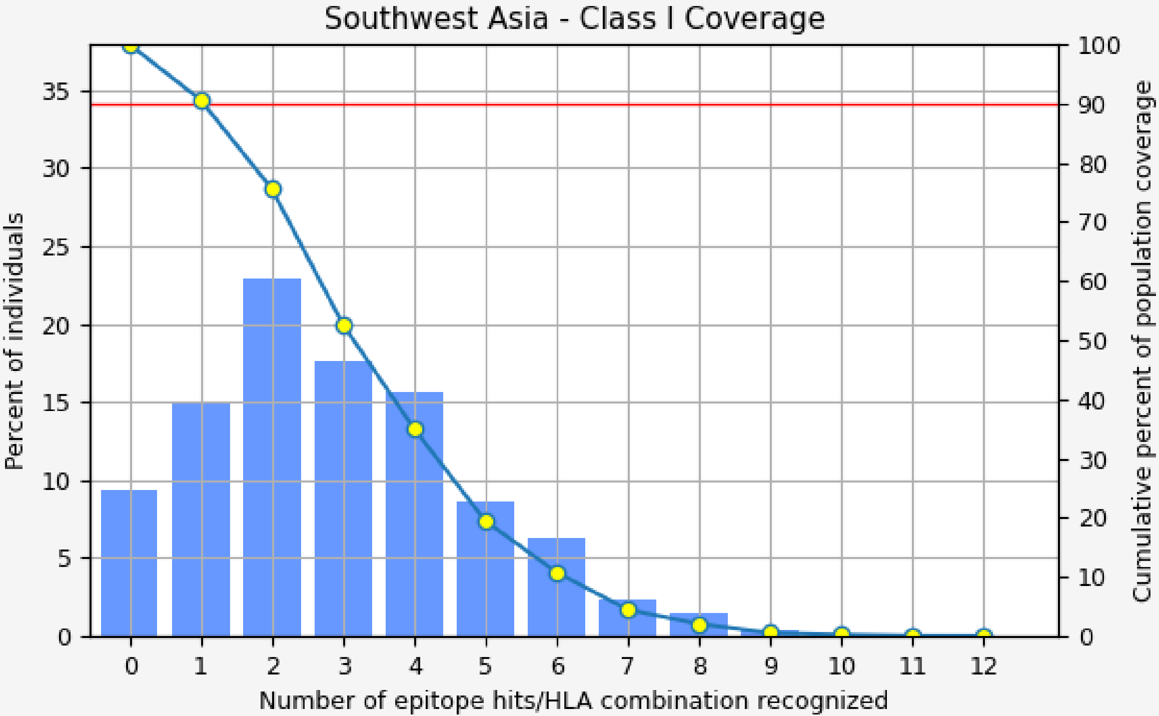

Southwest Asia - Class I Coverage
Percent of individuals
Cumulative percent of population coverage
Number of epitope hits/HLA combination recognized
0
5
10
15
20
25
30
35
0
10
20
30
40
50
60
70
80
90
100
0
1
2
3
4
5
6
7
8
9
10
11
12

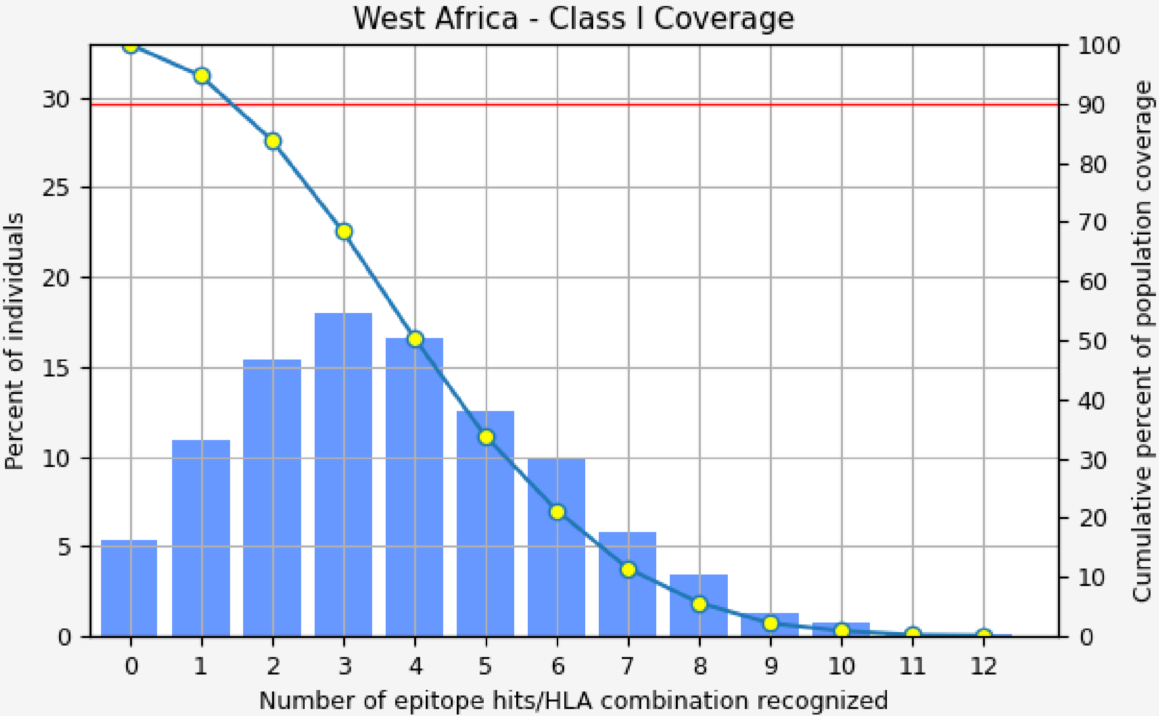
West Africa - Class I Coverage
Percent of individuals
Cumulative percent of population coverage
Number of epitope hits/HLA combination recognized
0
5
10
15
20
25
30
0
10
20
30
40
50
60
70
80
90
100
0
1
2
3
4
5
6
7
8
9
10
11
12

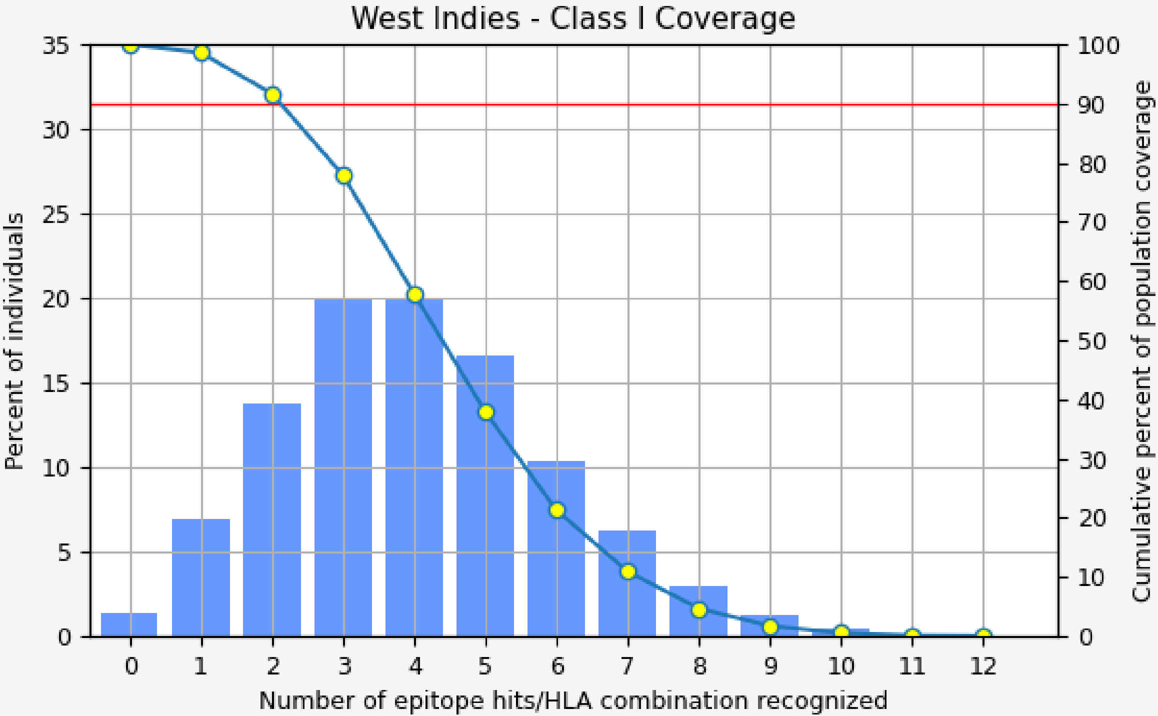
West Indies - Class I Coverage
Percent of individuals
Cumulative percent of population coverage
Number of epitope hits/HLA combination recognized
0 5 10 15 20 25 30 35
0 10 20 30 40 50 60 70 80 90 100
0 1 2 3 4 5 6 7 8 9 10 11 12
