# Supplementary material for: AutoEpiCollect, a Novel Machine Learning-Based GUI Software for Vaccine Design: Application to Pan-Cancer Vaccine Design Targeting PIK3CA Neoantigens
Source: Bioengineering (Basel). 2024 Mar 27;11(4):322. doi: 10.3390/bioengineering11040322 (PMC11048108; doi:10.3390/bioengineering11040322)
Supplement: Supplementary file 1 [file bioengineering-11-00322-s001.zip › Supplementary Data/Supplementary Figures.zip!/Supplementary Figures/Supplementary Figure S4.pdf]

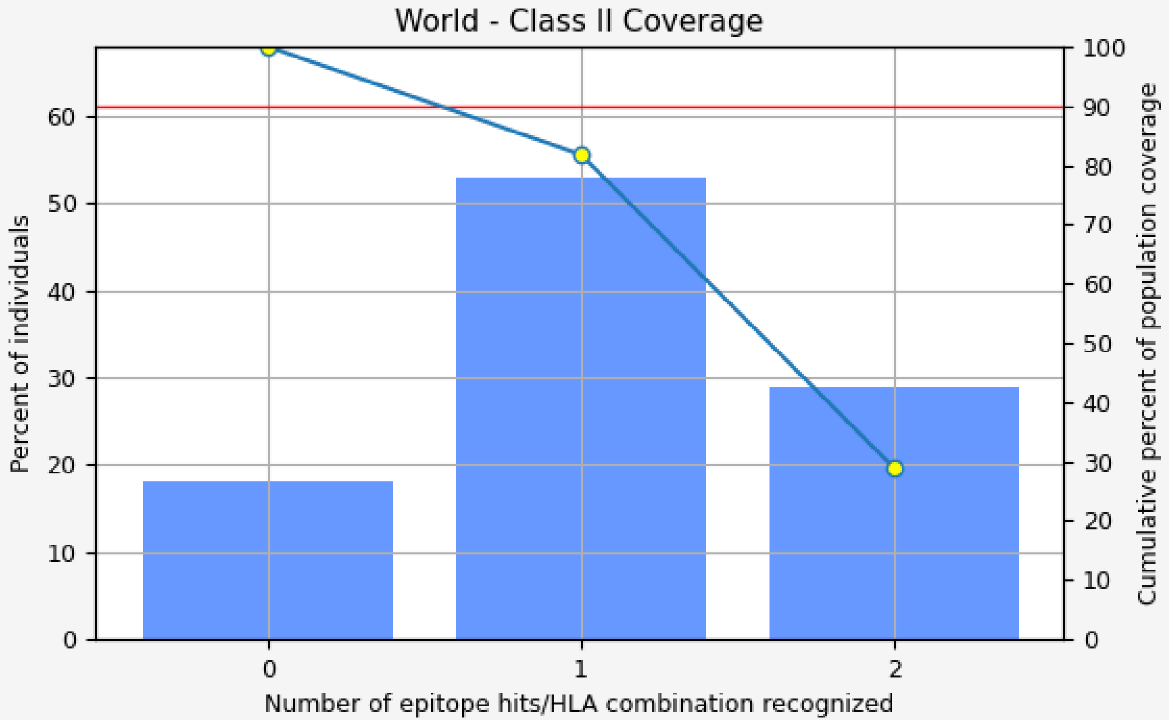
World - Class II Coverage
Percent of individuals
Cumulative percent of population coverage
Number of epitope hits/HLA combination recognized
0
1
2
0
10
20
30
40
50
60
0
10
20
30
40
50
60
70
80
90
100

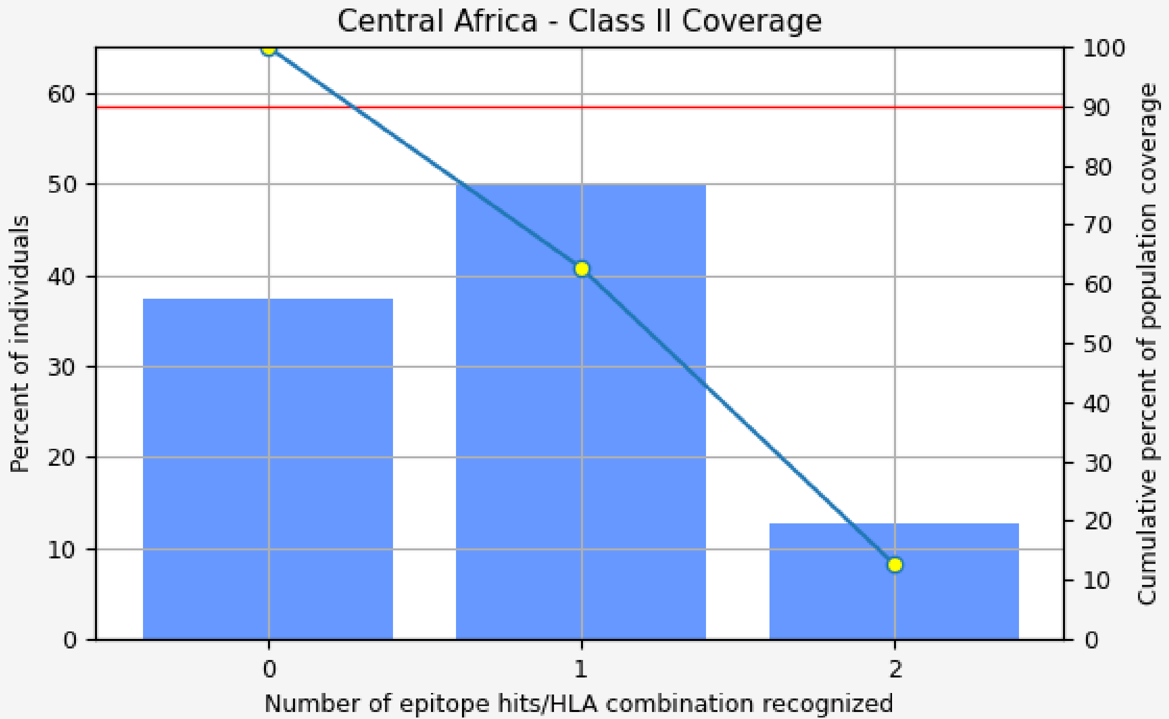

Central Africa - Class II Coverage
Percent of individuals
Cumulative percent of population coverage
Number of epitope hits/HLA combination recognized
0
10
20
30
40
50
60
0
10
20
30
40
50
60
70
80
90
100
0
1
2

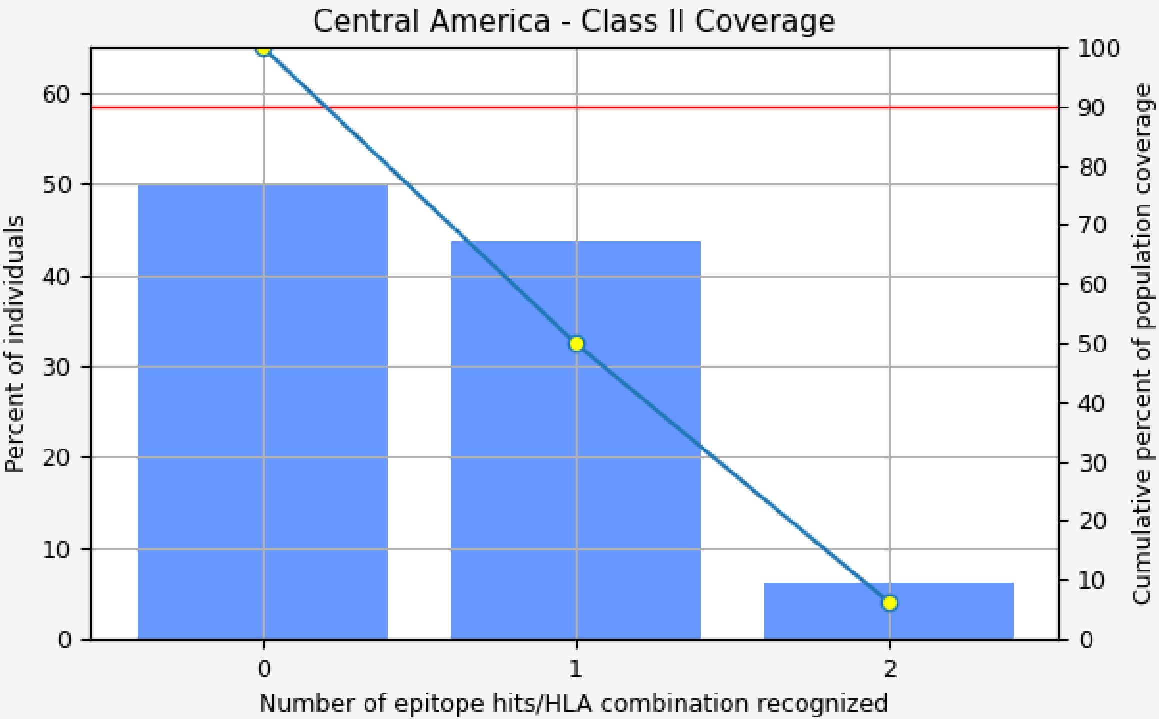

Central America - Class II Coverage
Percent of individuals
Cumulative percent of population coverage
Number of epitope hits/HLA combination recognized
0
10
20
30
40
50
60
70
80
90
100
1
2

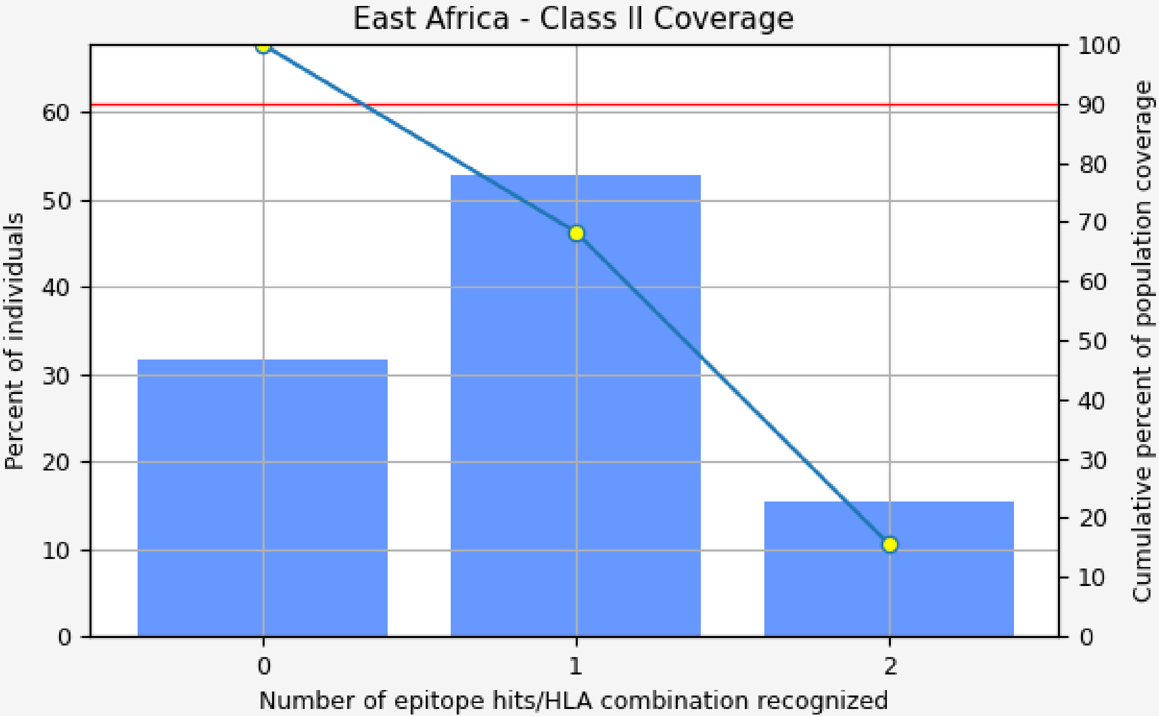

East Africa - Class II Coverage
Percent of individuals
Cumulative percent of population coverage
Number of epitope hits/HLA combination recognized
0
10
20
30
40
50
60
70
80
90
100
1
2

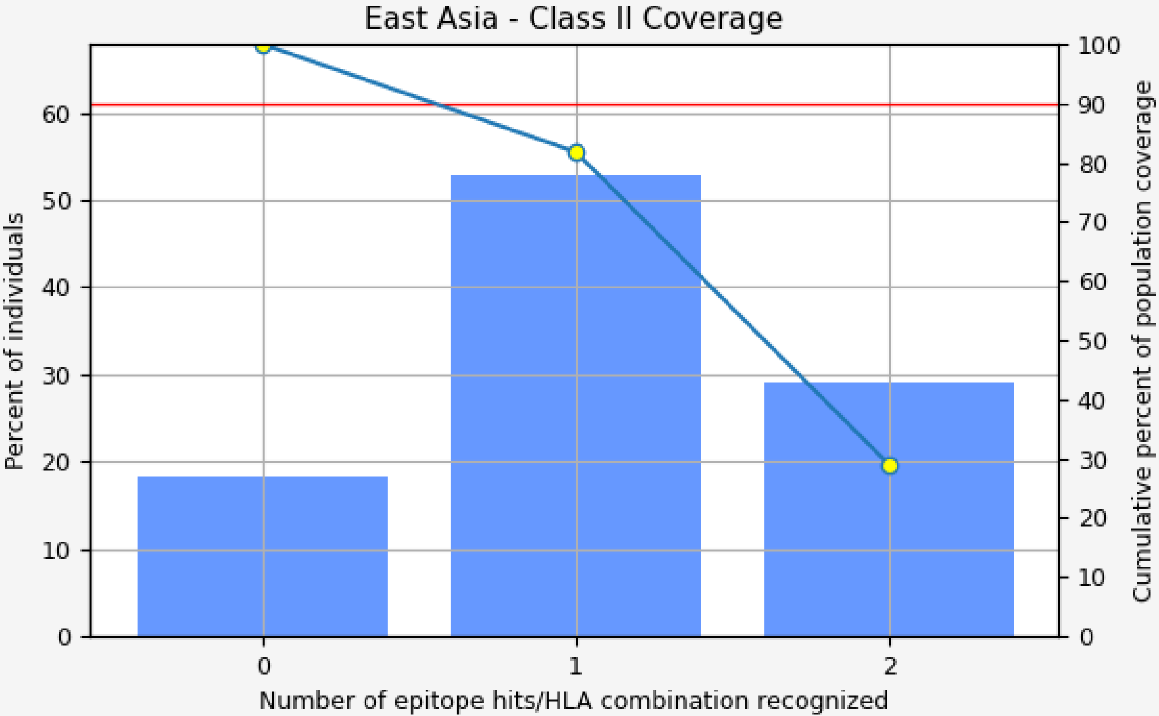
East Asia - Class II Coverage
Percent of individuals
Cumulative percent of population coverage
Number of epitope hits/HLA combination recognized
0
10
20
30
40
50
60
0
1
2
0
10
20
30
40
50
60
70
80
90
100

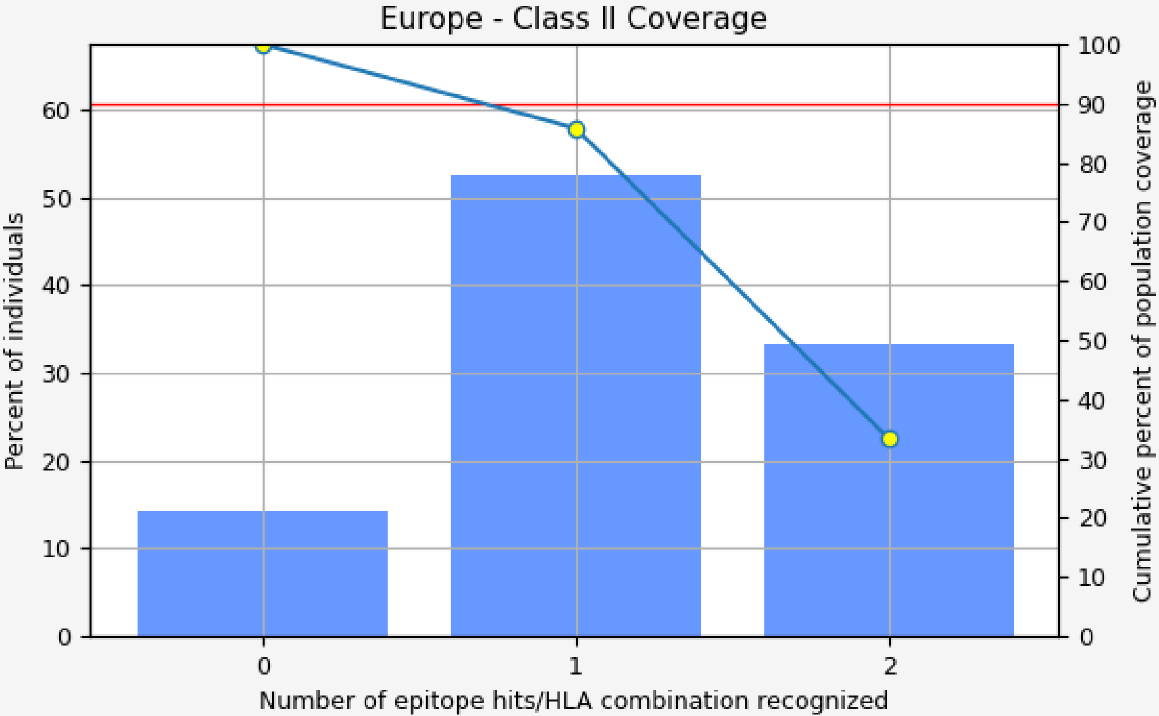
Europe - Class II Coverage
Percent of individuals
0
10
20
30
40
50
60
Cumulative percent of population coverage
0
10
20
30
40
50
60
70
80
90
100
0
1
2
Number of epitope hits/HLA combination recognized

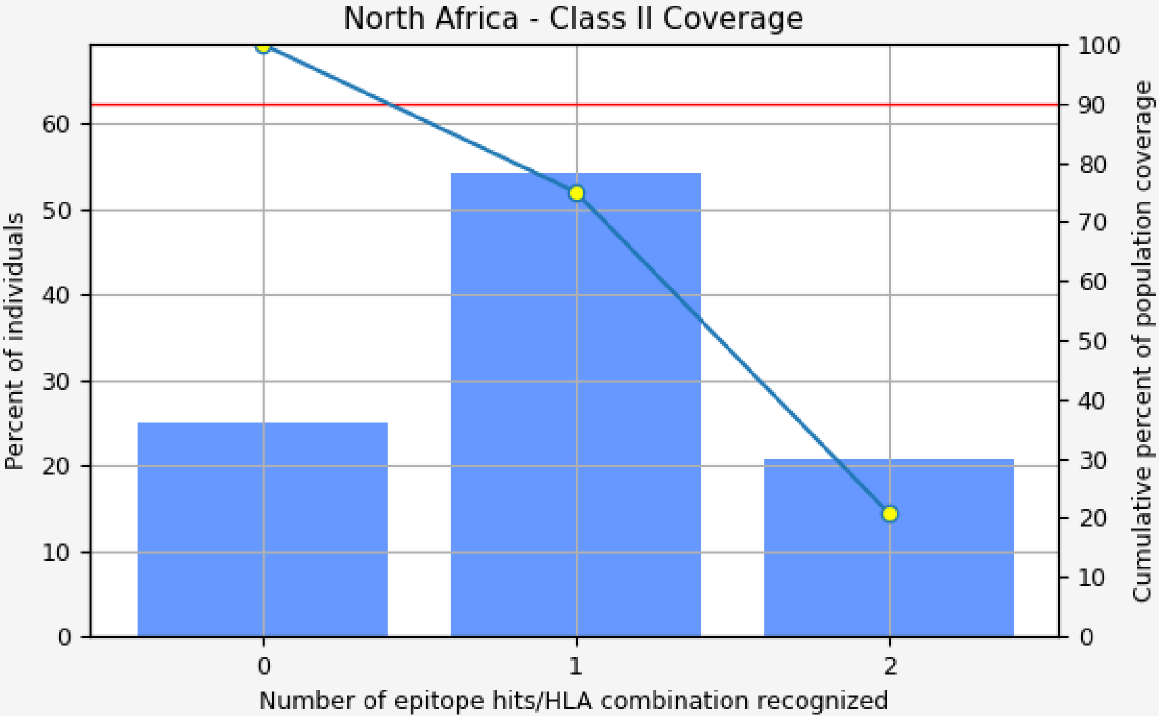
North Africa - Class II Coverage
Percent of individuals
Cumulative percent of population coverage
Number of epitope hits/HLA combination recognized
0
1
2
0
10
20
30
40
50
60
0
10
20
30
40
50
60
70
80
90
100

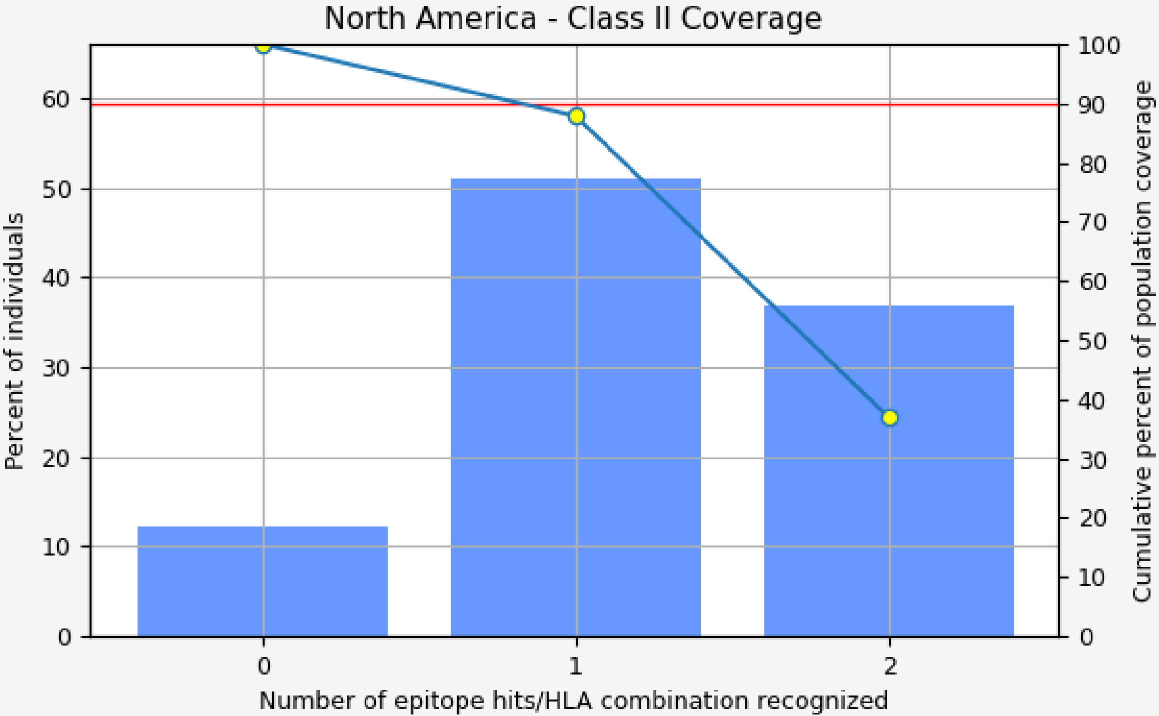
North America - Class II Coverage
Percent of individuals
0
10
20
30
40
50
60
Cumulative percent of population coverage
0
10
20
30
40
50
60
70
80
90
100
0
1
2
Number of epitope hits/HLA combination recognized

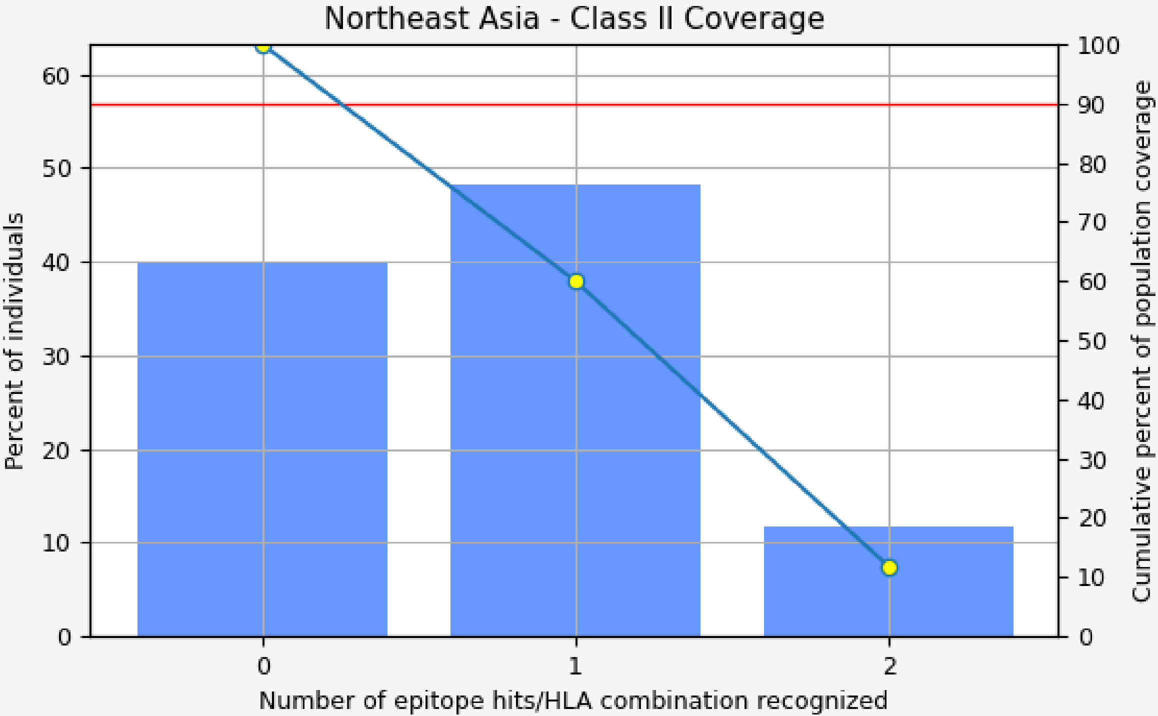
Northeast Asia - Class II Coverage
Percent of individuals
Cumulative percent of population coverage
Number of epitope hits/HLA combination recognized
0
10
20
30
40
50
60
0
1
2
0
10
20
30
40
50
60
70
80
90
100

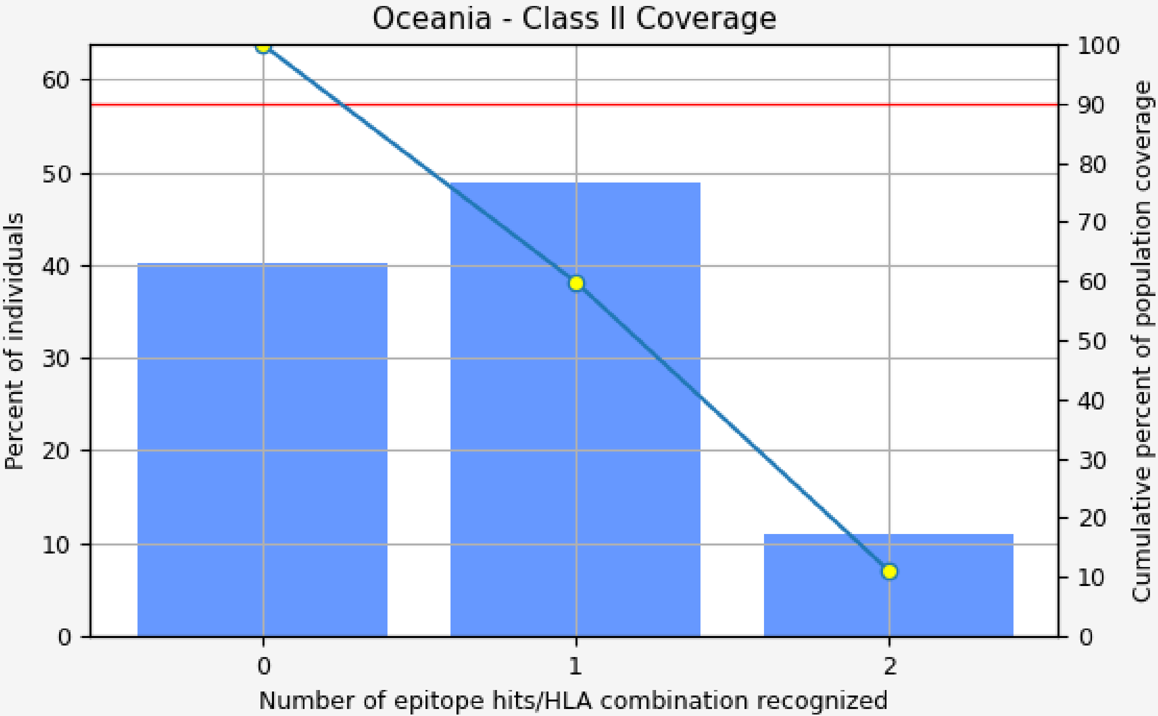
Oceania - Class II Coverage
Percent of individuals
Cumulative percent of population coverage
Number of epitope hits/HLA combination recognized
0
10
20
30
40
50
60
70
80
90
100
1
2

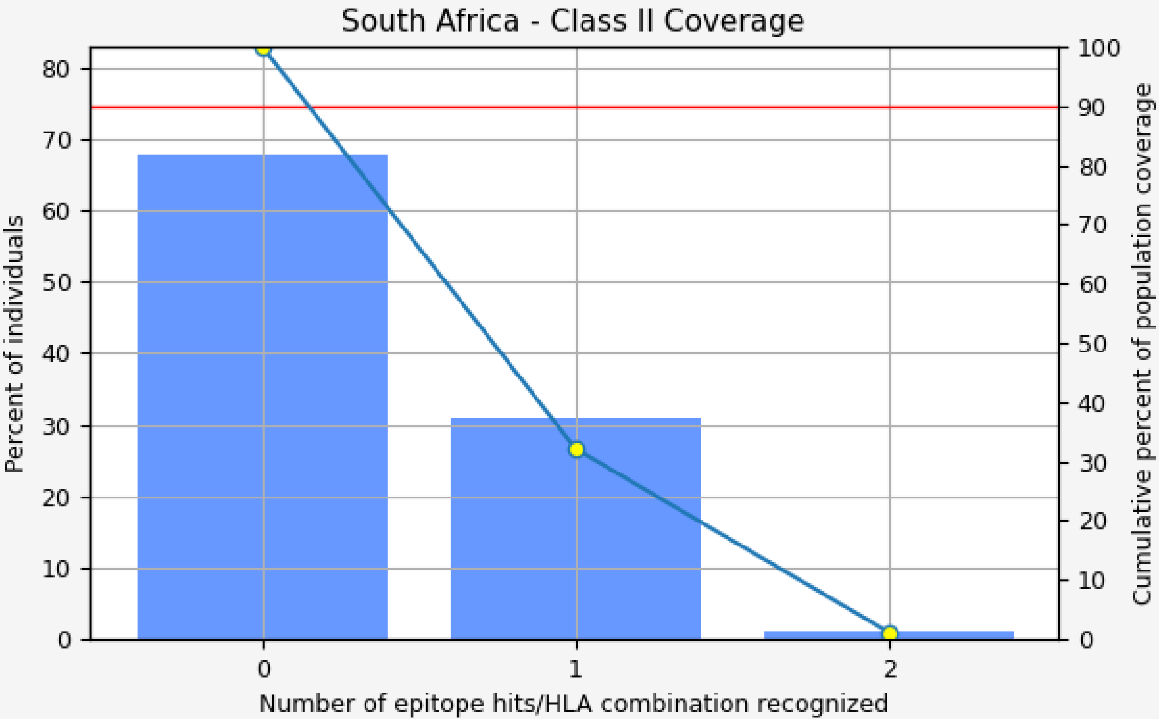
South Africa - Class II Coverage
Percent of individuals
Cumulative percent of population coverage
Number of epitope hits/HLA combination recognized
0
10
20
30
40
50
60
70
80
90
100
1
2

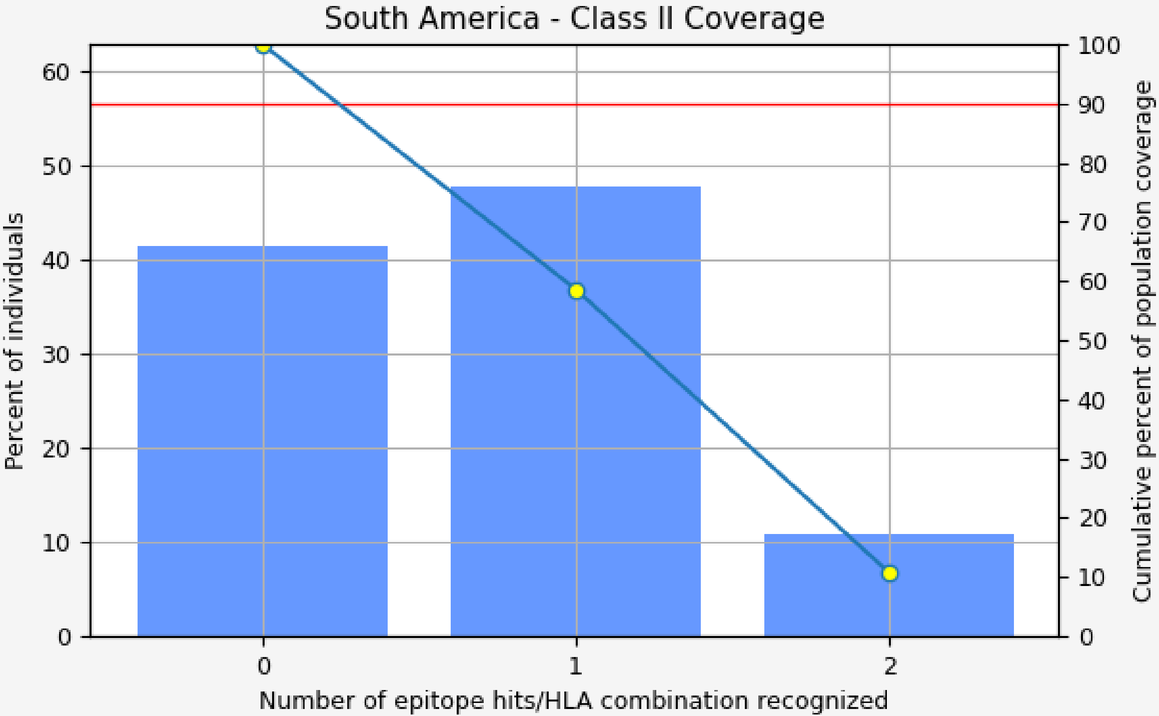

South America - Class II Coverage
Percent of individuals
Cumulative percent of population coverage
Number of epitope hits/HLA combination recognized
0
10
20
30
40
50
60
70
80
90
100
0
1
2

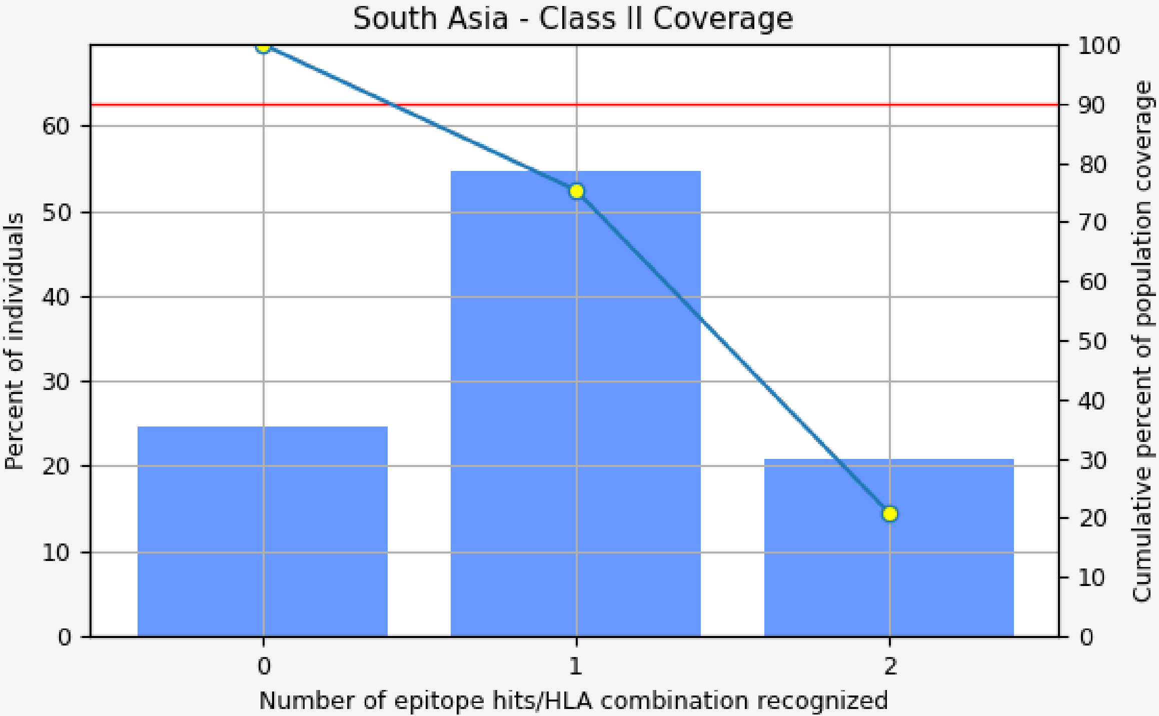
South Asia - Class II Coverage
Percent of individuals
Cumulative percent of population coverage
Number of epitope hits/HLA combination recognized
0
10
20
30
40
50
60
0
10
20
30
40
50
60
70
80
90
100
0
1
2

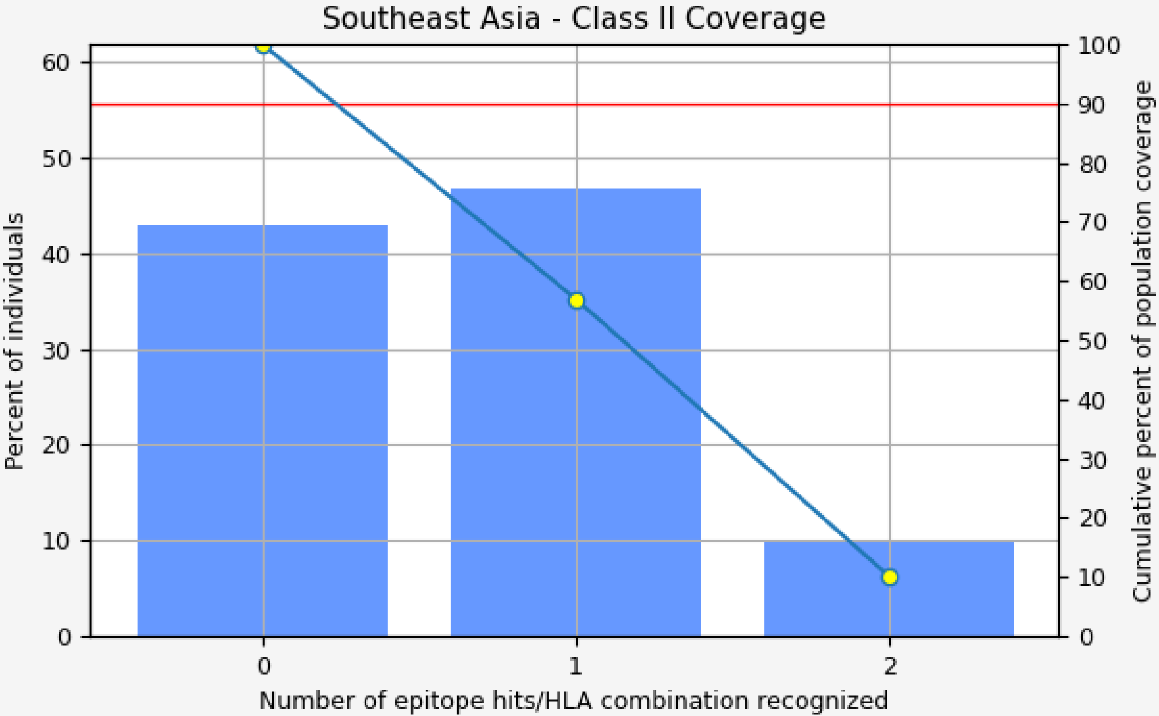

Southeast Asia - Class II Coverage
Percent of individuals
Cumulative percent of population coverage
Number of epitope hits/HLA combination recognized
0
10
20
30
40
50
60
0
10
20
30
40
50
60
70
80
90
100
0
1
2

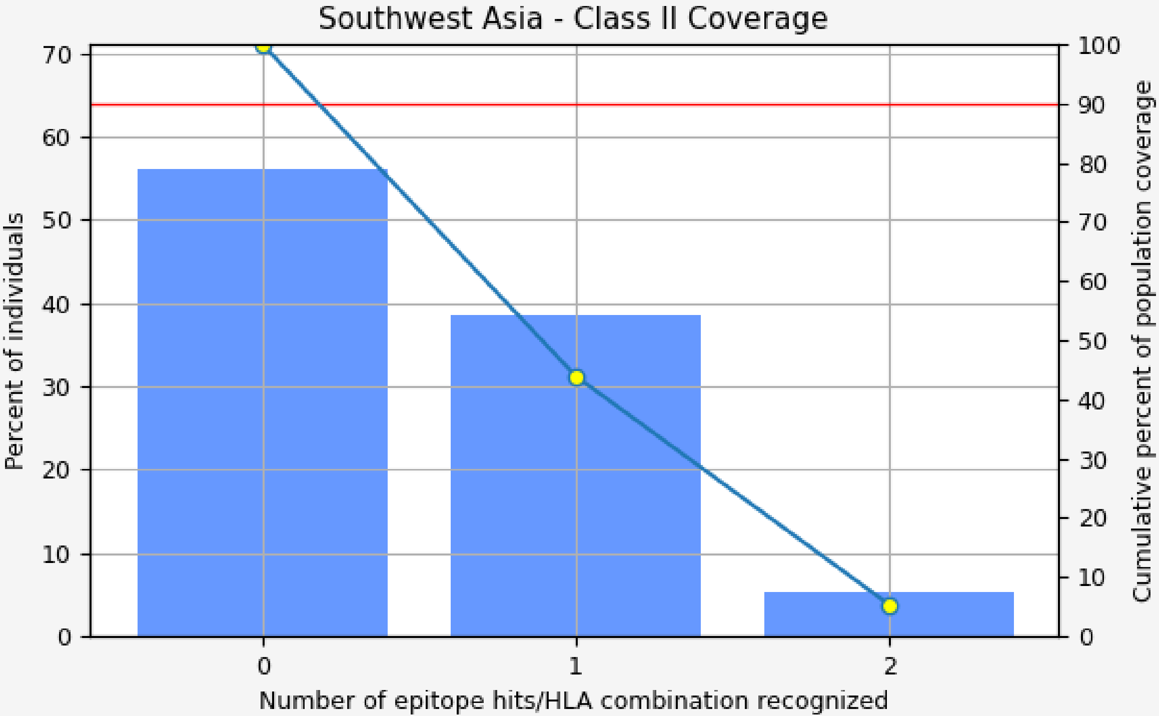

Southwest Asia - Class II Coverage
Percent of individuals
70
60
50
40
30
20
10
0
100
90
80
70
60
50
40
30
20
10
0
Cumulative percent of population coverage
0
1
2
Number of epitope hits/HLA combination recognized

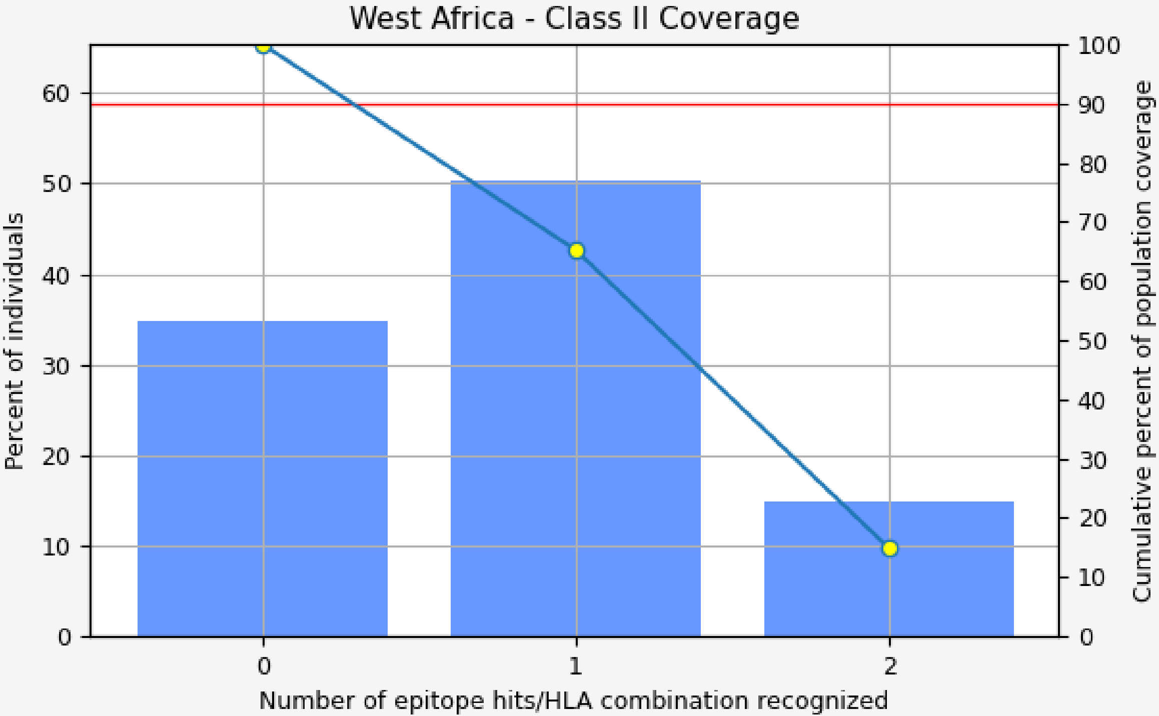
West Africa - Class II Coverage
Percent of individuals
0
10
20
30
40
50
60
Cumulative percent of population coverage
0
10
20
30
40
50
60
70
80
90
100
0
1
2
Number of epitope hits/HLA combination recognized

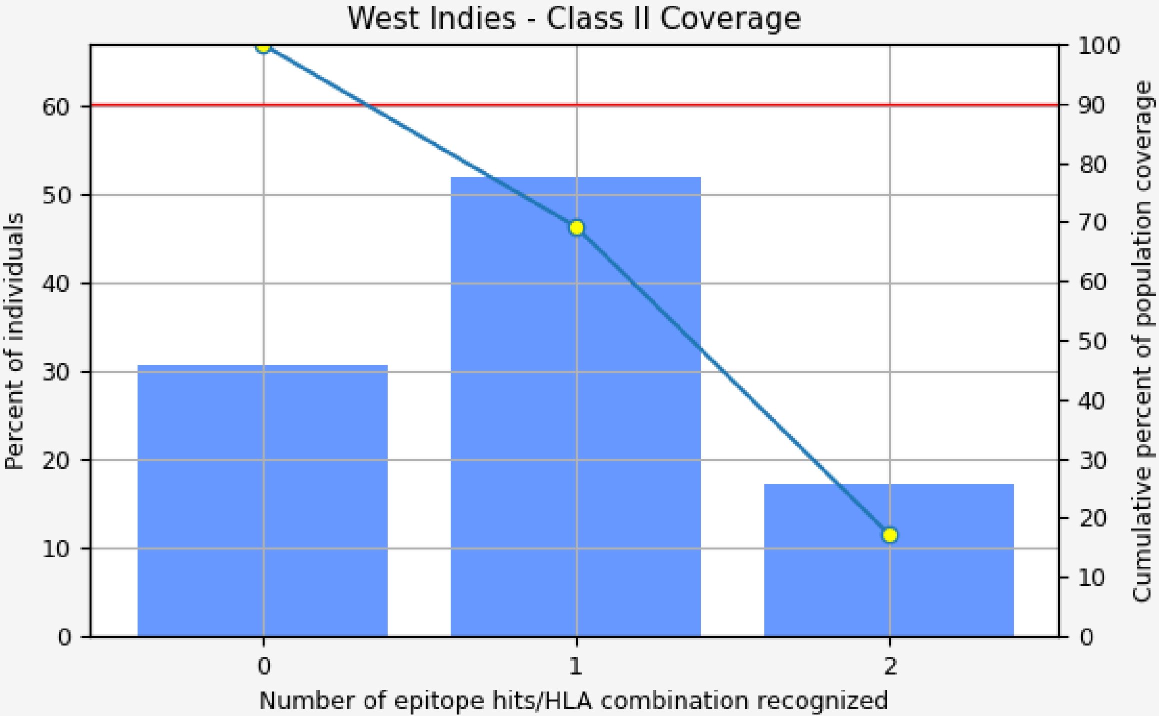
West Indies - Class II Coverage
Percent of individuals
Cumulative percent of population coverage
Number of epitope hits/HLA combination recognized
0
10
20
30
40
50
60
0
10
20
30
40
50
60
70
80
90
100
0
1
2
