# Supplementary material for: AutoEpiCollect, a Novel Machine Learning-Based GUI Software for Vaccine Design: Application to Pan-Cancer Vaccine Design Targeting PIK3CA Neoantigens
Source: Bioengineering (Basel). 2024 Mar 27;11(4):322. doi: 10.3390/bioengineering11040322 (PMC11048108; doi:10.3390/bioengineering11040322)
Supplement: Supplementary file 1 [file bioengineering-11-00322-s001.zip › Supplementary Data/Supplementary Figures.zip!/Supplementary Figures/Supplementary Figure S3.pdf]

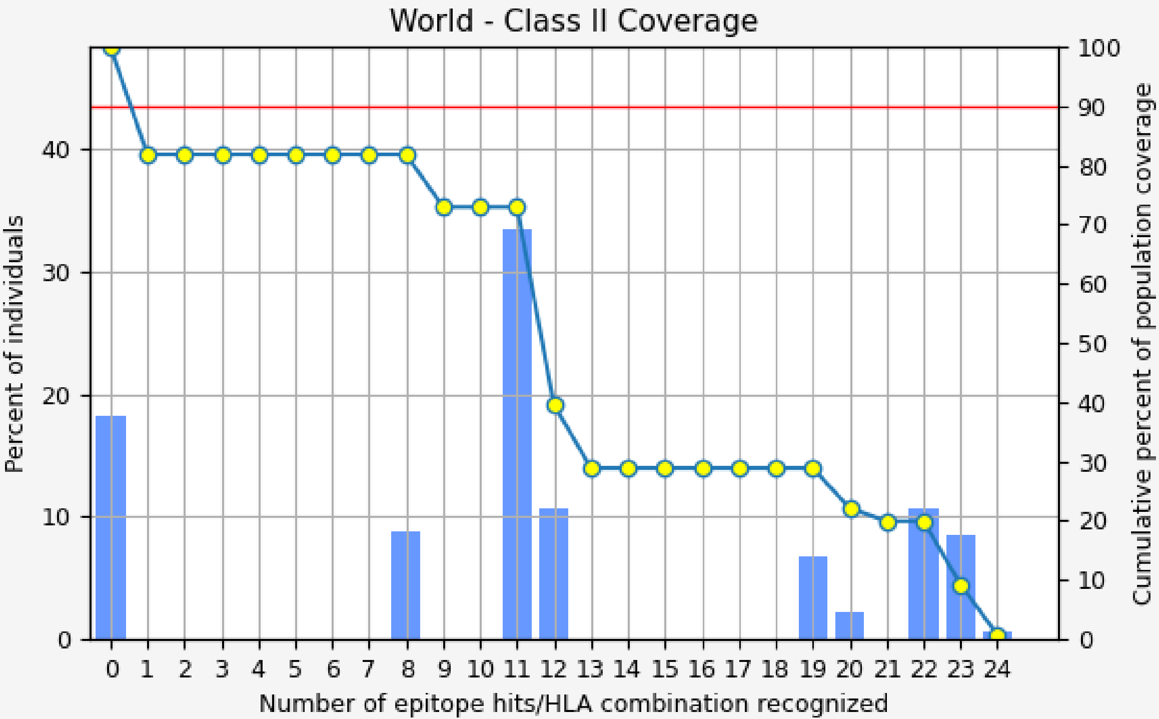

World - Class II Coverage
Percent of individuals
Cumulative percent of population coverage
Number of epitope hits/HLA combination recognized
0
10
20
30
40
0
10
20
30
40
50
60
70
80
90
100
0 1 2 3 4 5 6 7 8 9 10 11 12 13 14 15 16 17 18 19 20 21 22 23 24

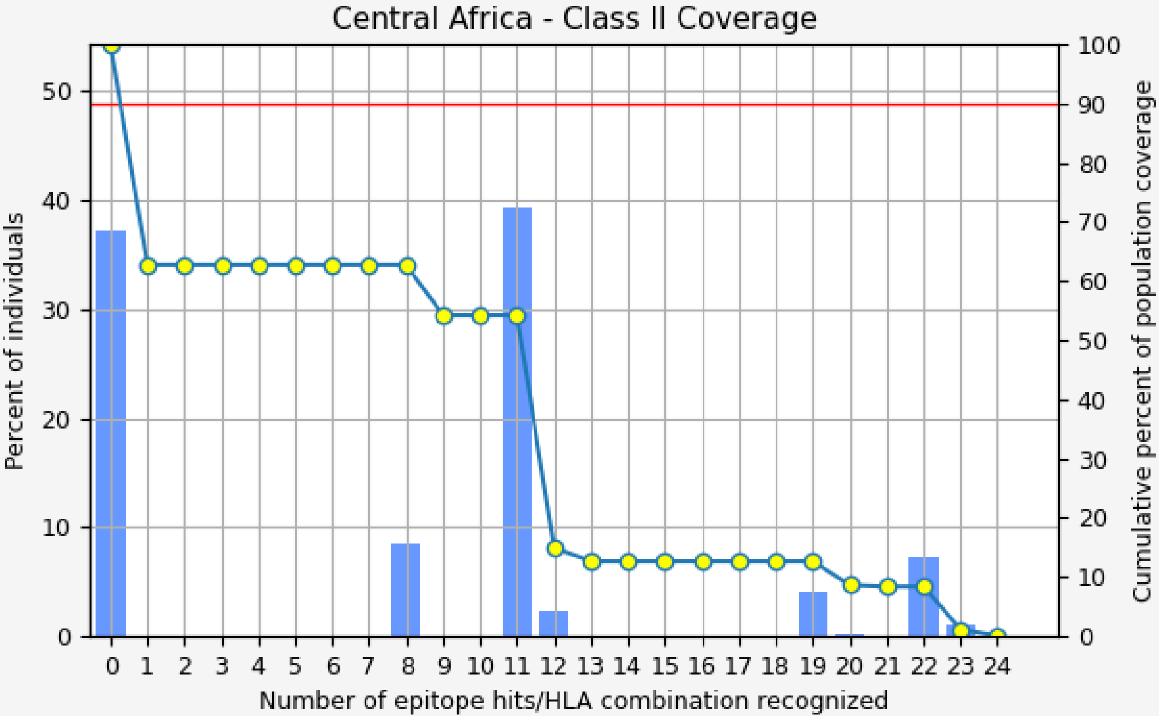

Central Africa - Class II Coverage
Percent of individuals
Cumulative percent of population coverage
Number of epitope hits/HLA combination recognized
0
10
20
30
40
50
0
10
20
30
40
50
60
70
80
90
100
0 1 2 3 4 5 6 7 8 9 10 11 12 13 14 15 16 17 18 19 20 21 22 23 24

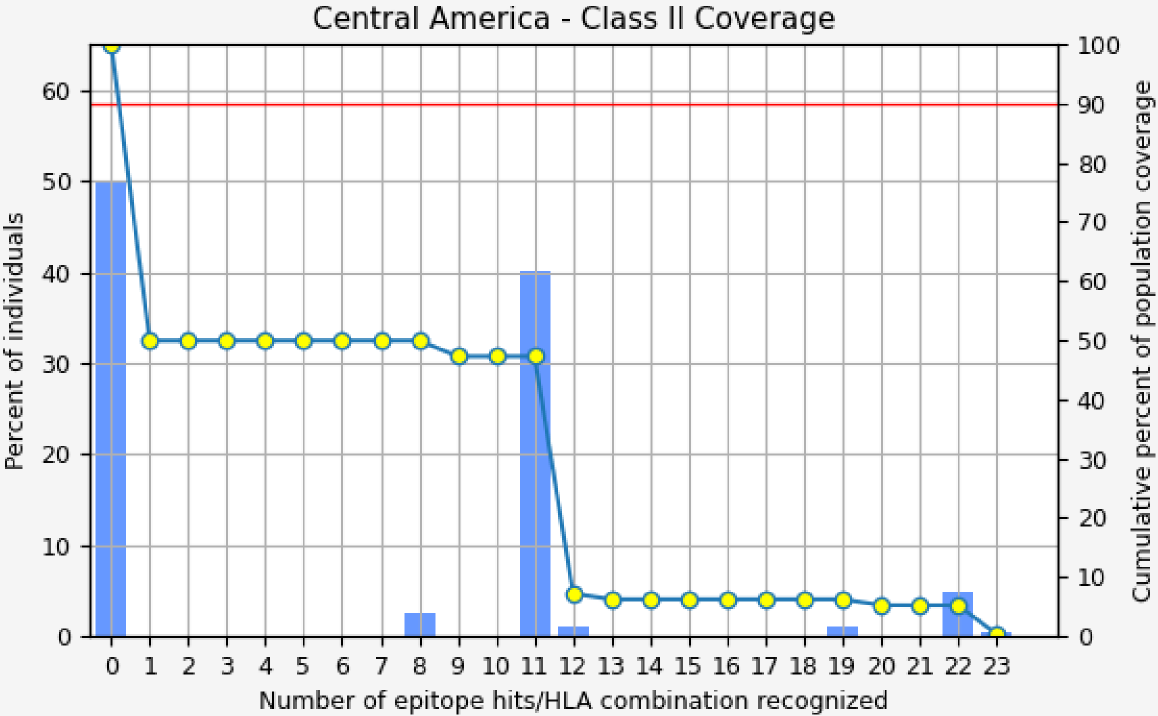
Central America - Class II Coverage
Percent of individuals
Cumulative percent of population coverage
Number of epitope hits/HLA combination recognized
0
10
20
30
40
50
60
70
80
90
100
0 1 2 3 4 5 6 7 8 9 10 11 12 13 14 15 16 17 18 19 20 21 22 23

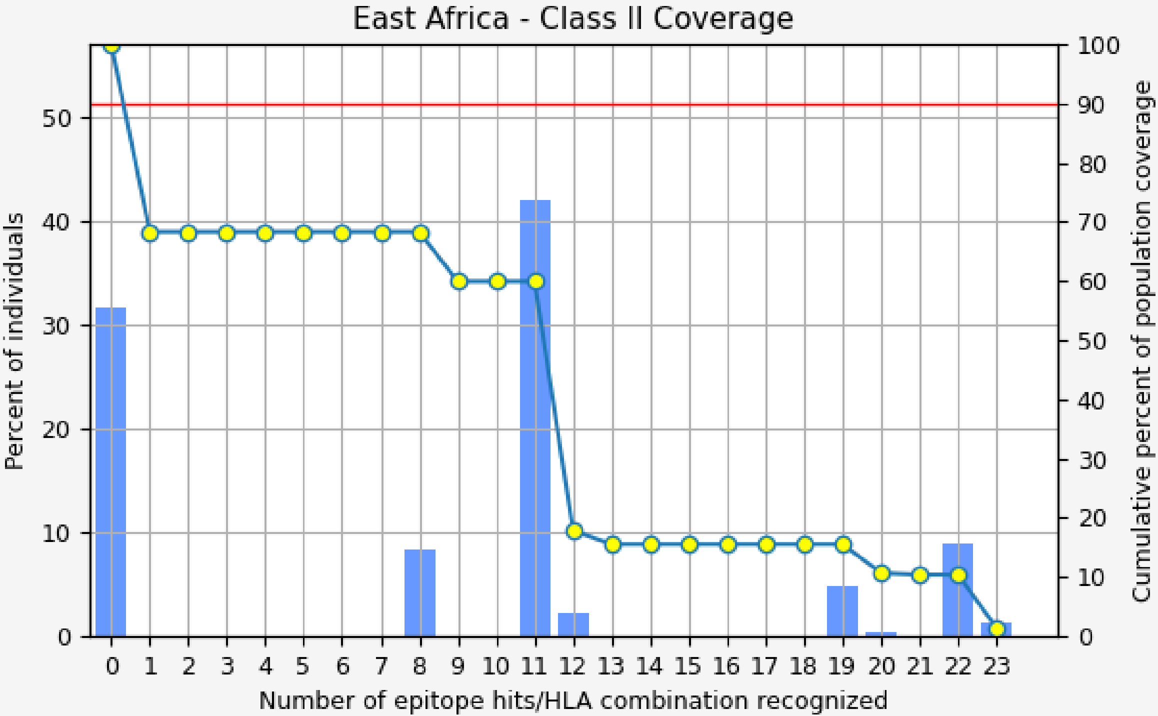
East Africa - Class II Coverage
Percent of individuals
Cumulative percent of population coverage
Number of epitope hits/HLA combination recognized
0
10
20
30
40
50
0
10
20
30
40
50
60
70
80
90
100
0 1 2 3 4 5 6 7 8 9 10 11 12 13 14 15 16 17 18 19 20 21 22 23

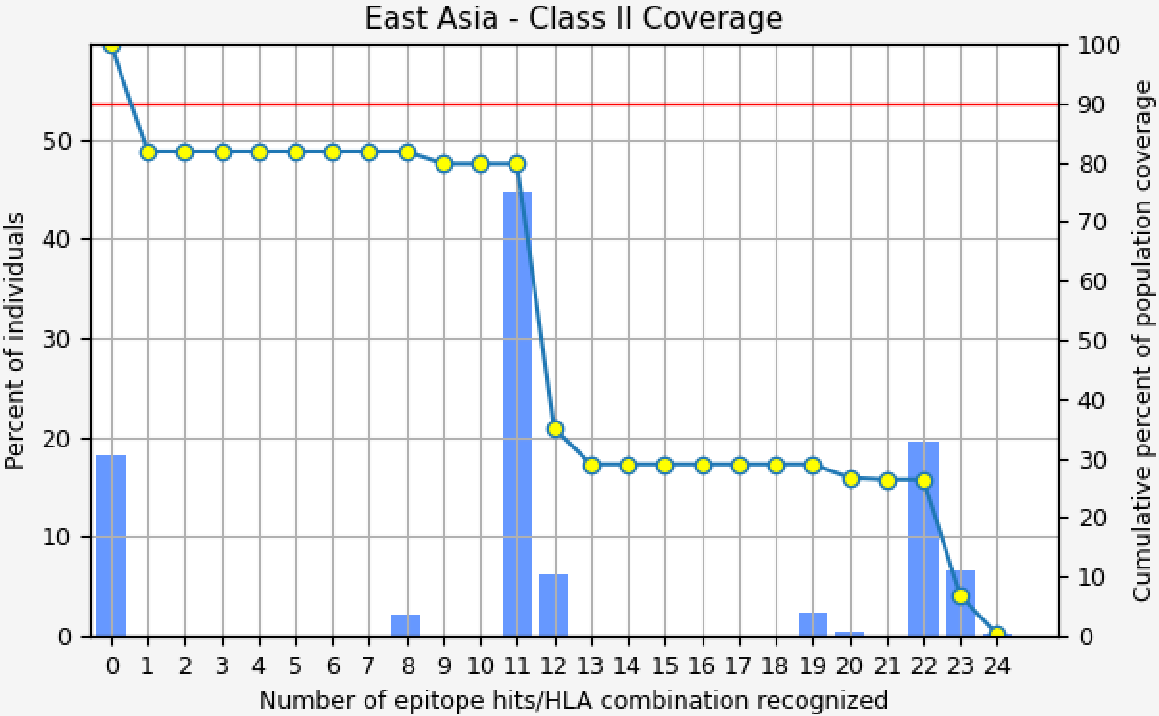

East Asia - Class II Coverage
Percent of individuals
Cumulative percent of population coverage
Number of epitope hits/HLA combination recognized
0
10
20
30
40
50
60
70
80
90
100
0 1 2 3 4 5 6 7 8 9 10 11 12 13 14 15 16 17 18 19 20 21 22 23 24

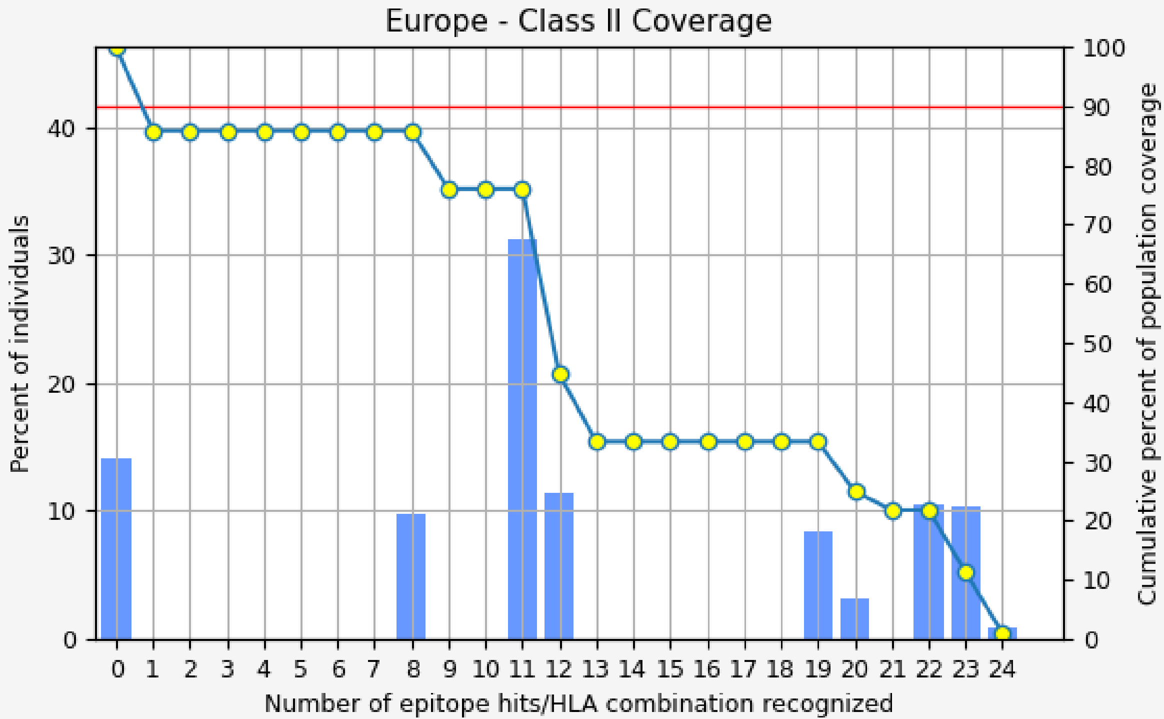

Europe - Class II Coverage
Percent of individuals
Cumulative percent of population coverage
Number of epitope hits/HLA combination recognized
0
10
20
30
40
0
10
20
30
40
50
60
70
80
90
100
0 1 2 3 4 5 6 7 8 9 10 11 12 13 14 15 16 17 18 19 20 21 22 23 24

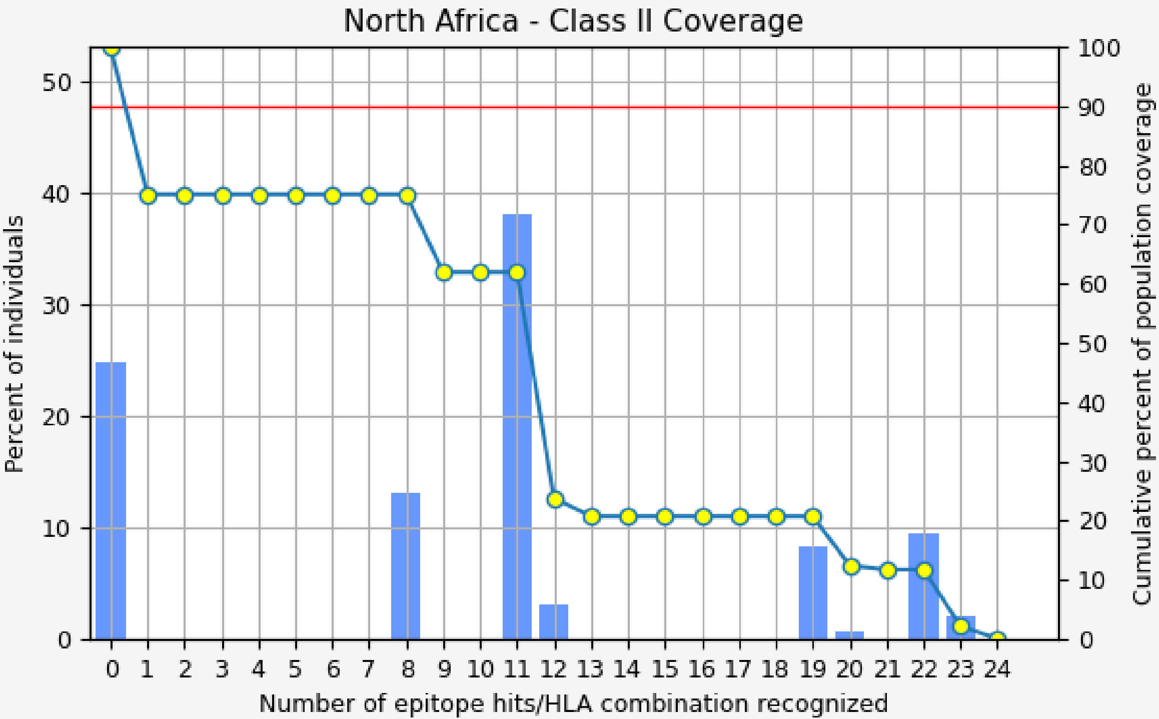
North Africa - Class II Coverage
Percent of individuals
Cumulative percent of population coverage
Number of epitope hits/HLA combination recognized
0
10
20
30
40
50
0
10
20
30
40
50
60
70
80
90
100
0 1 2 3 4 5 6 7 8 9 10 11 12 13 14 15 16 17 18 19 20 21 22 23 24

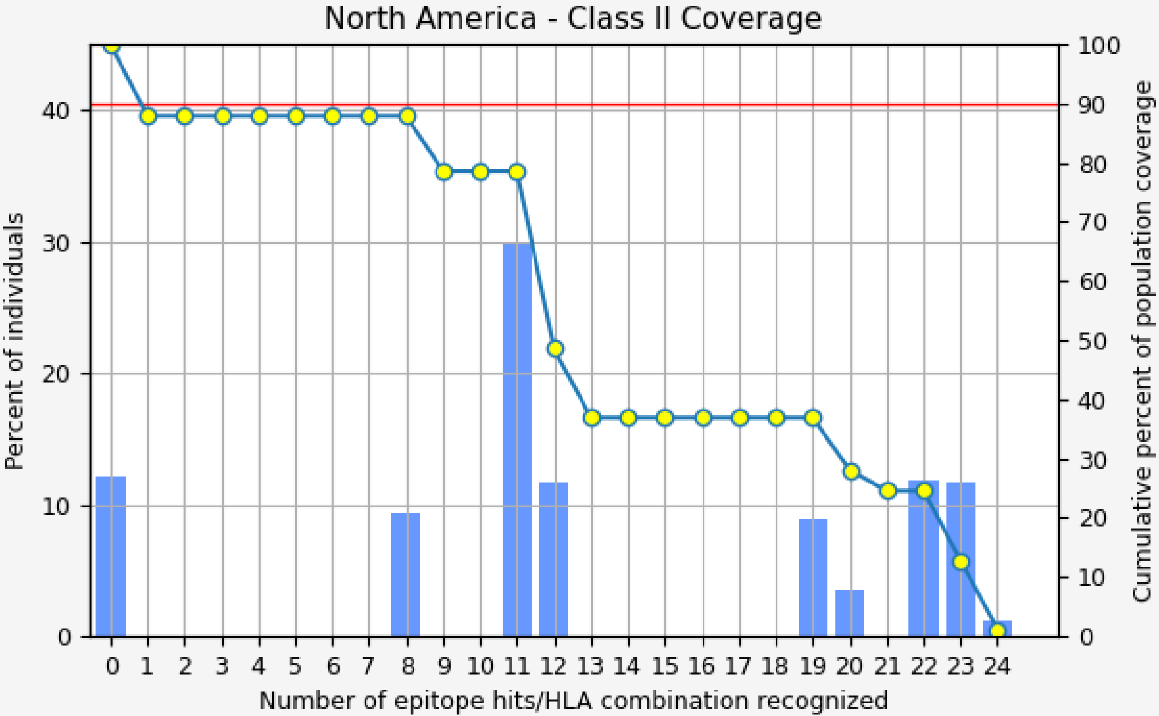

North America - Class II Coverage
Percent of individuals
Cumulative percent of population coverage
Number of epitope hits/HLA combination recognized
0
10
20
30
40
0
10
20
30
40
50
60
70
80
90
100
0 1 2 3 4 5 6 7 8 9 10 11 12 13 14 15 16 17 18 19 20 21 22 23 24

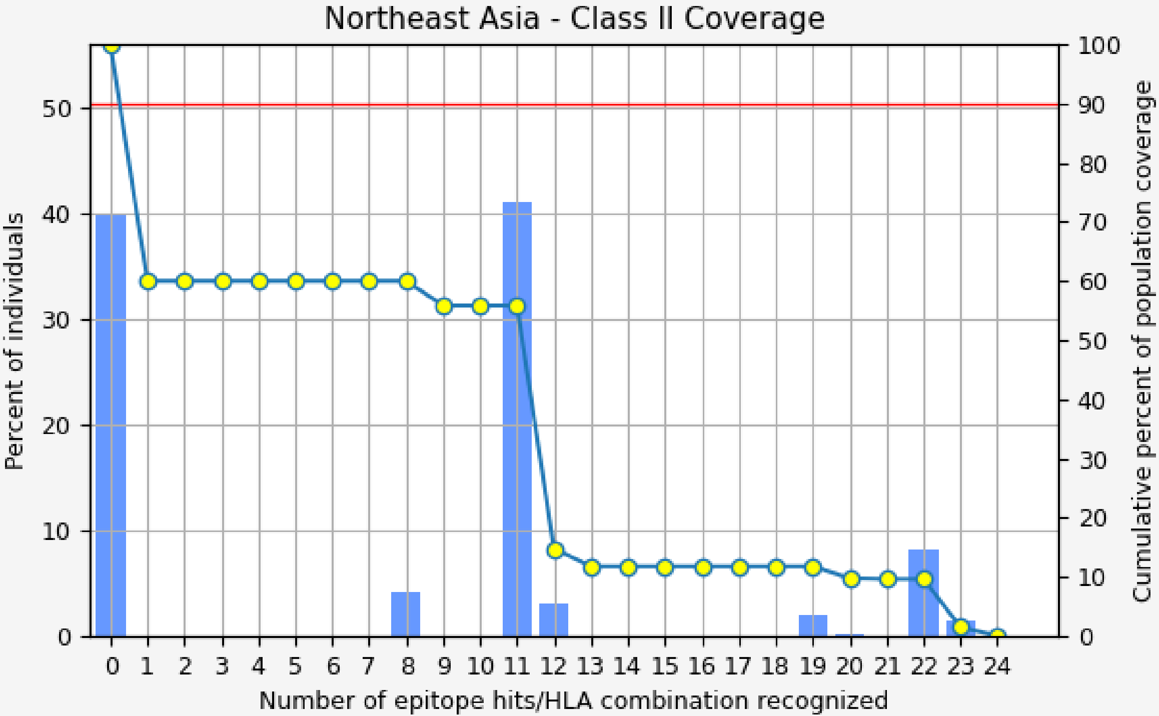

Northeast Asia - Class II Coverage
Percent of individuals
Cumulative percent of population coverage
Number of epitope hits/HLA combination recognized
0 10 20 30 40 50
0 10 20 30 40 50 60 70 80 90 100
0 1 2 3 4 5 6 7 8 9 10 11 12 13 14 15 16 17 18 19 20 21 22 23 24

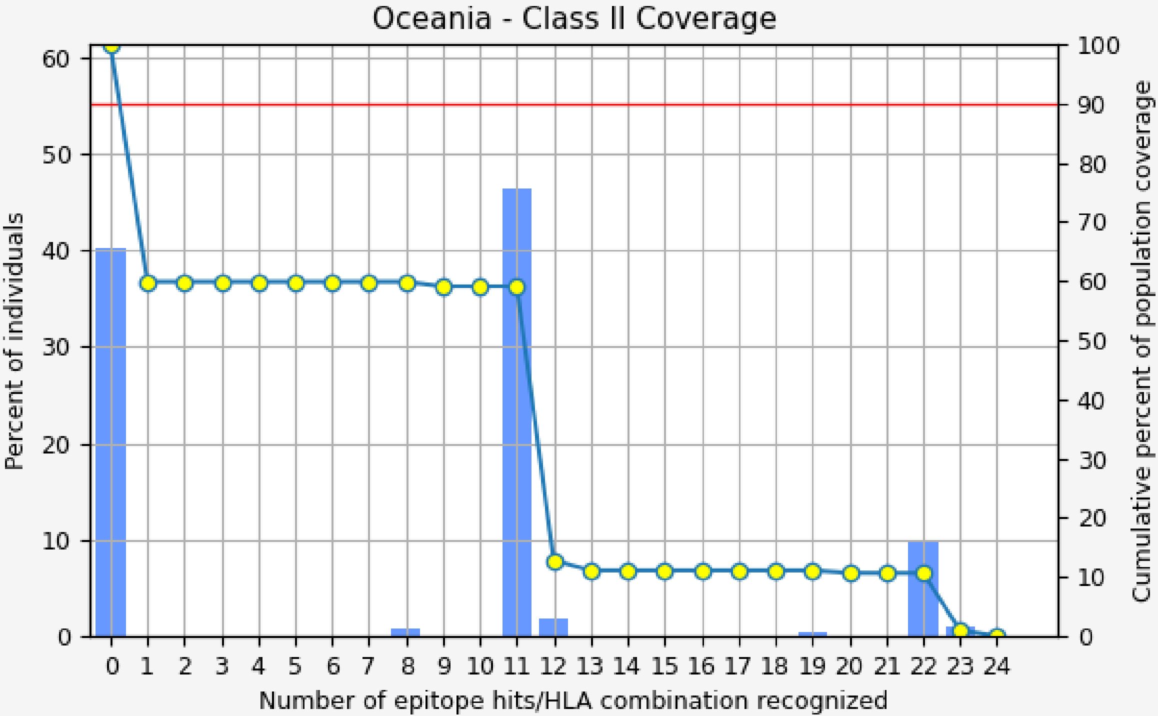
Oceania - Class II Coverage
Percent of individuals
Cumulative percent of population coverage
Number of epitope hits/HLA combination recognized
0
10
20
30
40
50
60
70
80
90
100
0 1 2 3 4 5 6 7 8 9 10 11 12 13 14 15 16 17 18 19 20 21 22 23 24

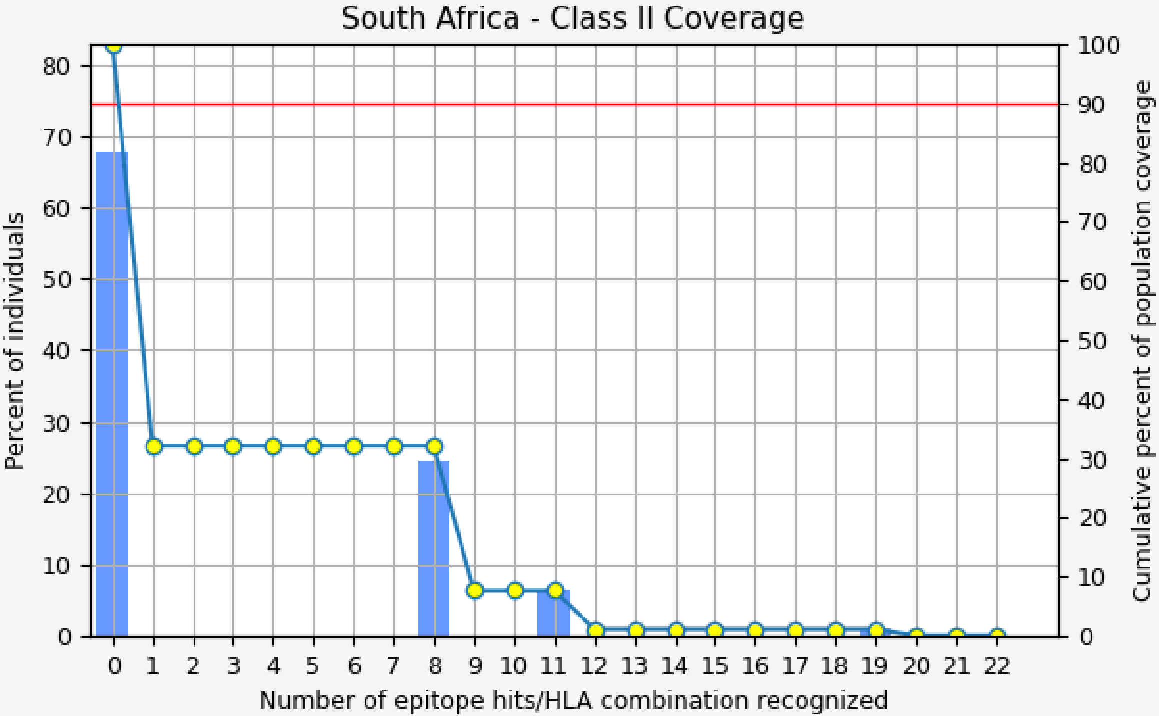
South Africa - Class II Coverage
Percent of individuals
Cumulative percent of population coverage
Number of epitope hits/HLA combination recognized
0
10
20
30
40
50
60
70
80
90
100
0 1 2 3 4 5 6 7 8 9 10 11 12 13 14 15 16 17 18 19 20 21 22

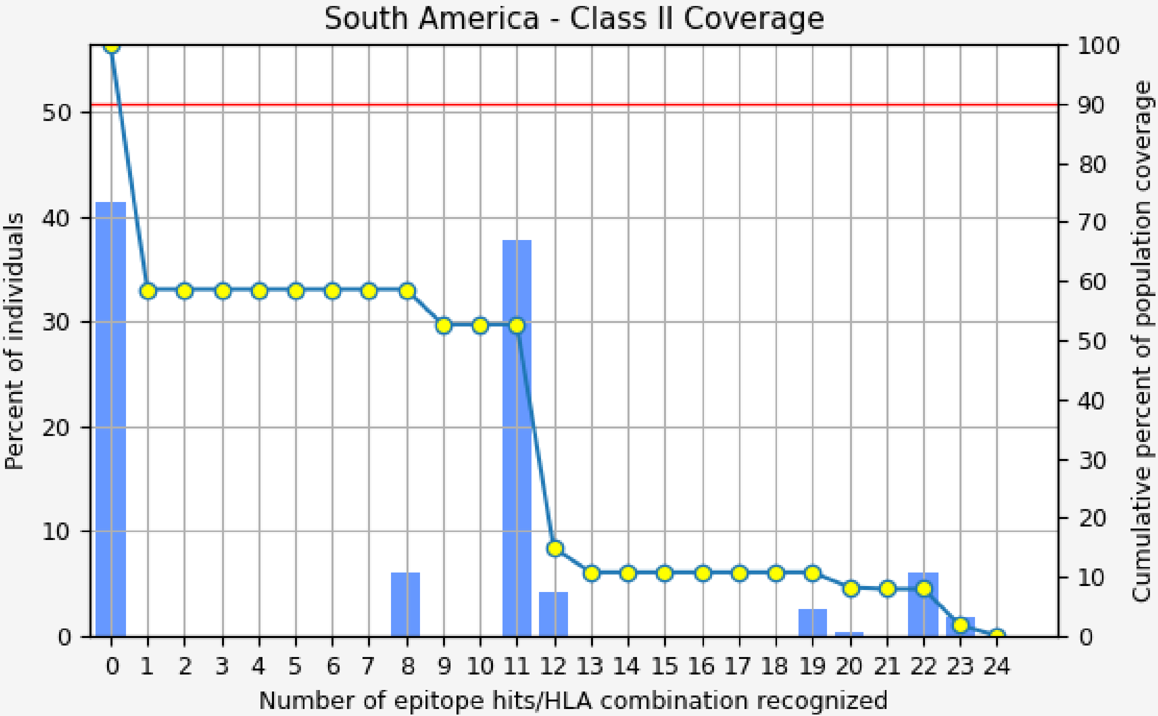

South America - Class II Coverage
Percent of individuals
Cumulative percent of population coverage
Number of epitope hits/HLA combination recognized
0
10
20
30
40
50
60
70
80
90
100
0 1 2 3 4 5 6 7 8 9 10 11 12 13 14 15 16 17 18 19 20 21 22 23 24

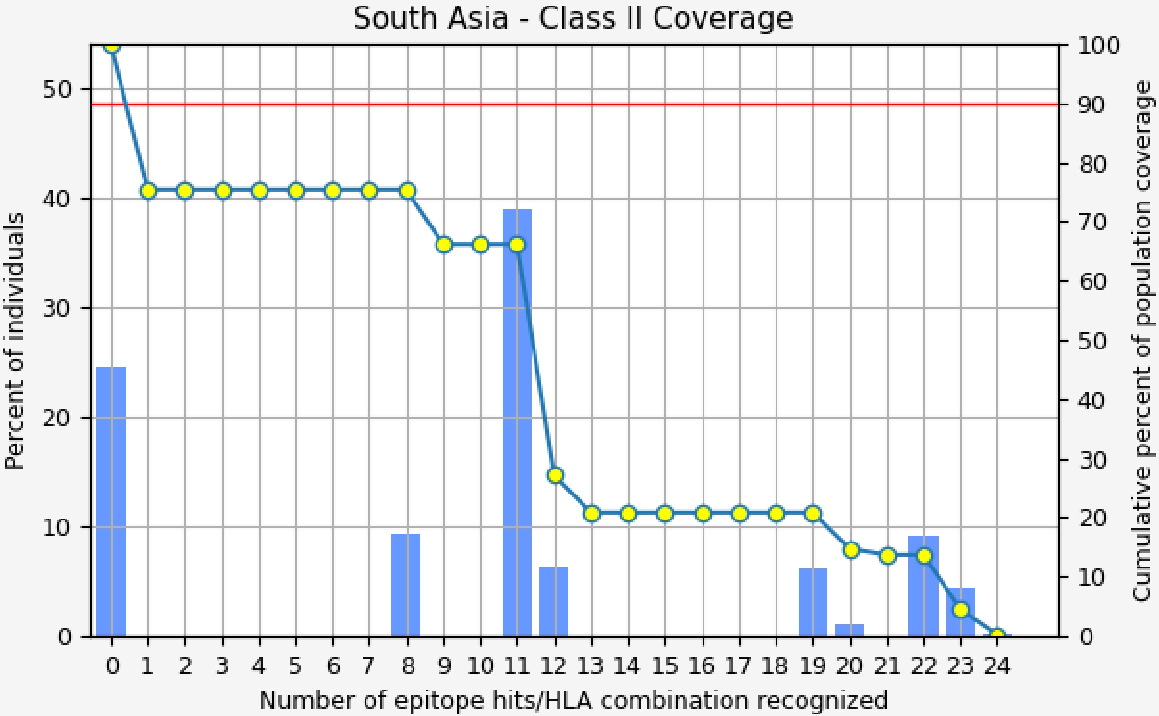
South Asia - Class II Coverage
Percent of individuals
Cumulative percent of population coverage
Number of epitope hits/HLA combination recognized
0
10
20
30
40
50
0
10
20
30
40
50
60
70
80
90
100
0 1 2 3 4 5 6 7 8 9 10 11 12 13 14 15 16 17 18 19 20 21 22 23 24

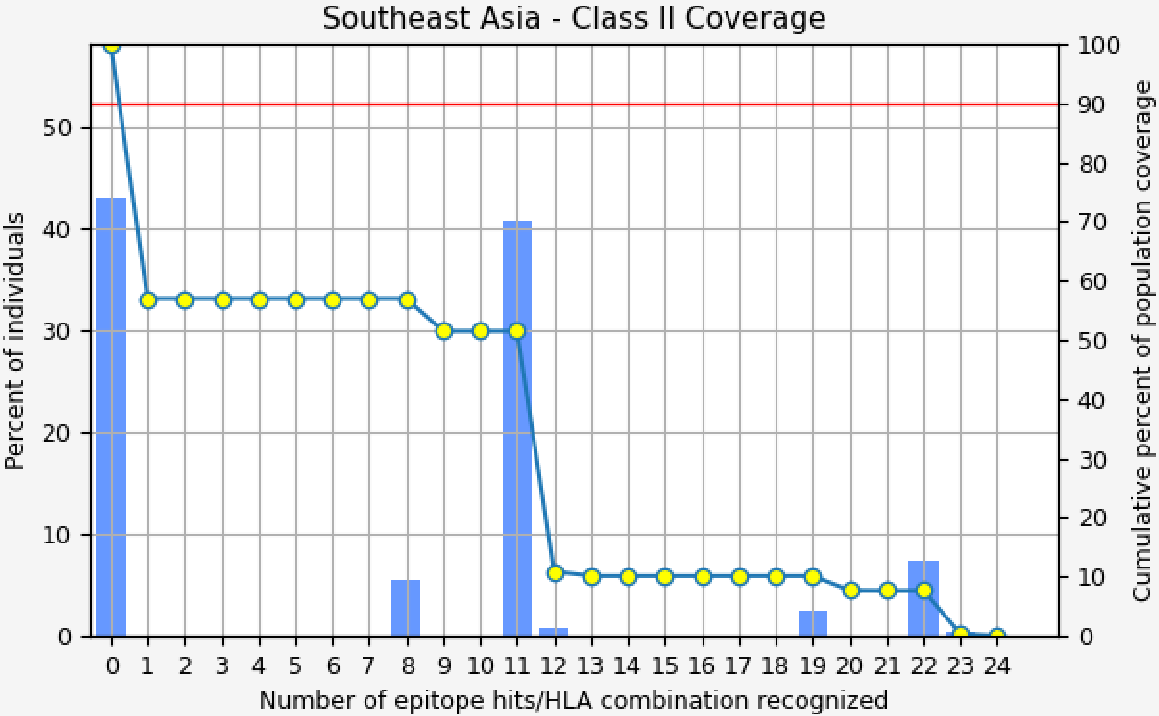
Southeast Asia - Class II Coverage
Percent of individuals
Cumulative percent of population coverage
Number of epitope hits/HLA combination recognized
0
10
20
30
40
50
0
10
20
30
40
50
60
70
80
90
100
0 1 2 3 4 5 6 7 8 9 10 11 12 13 14 15 16 17 18 19 20 21 22 23 24

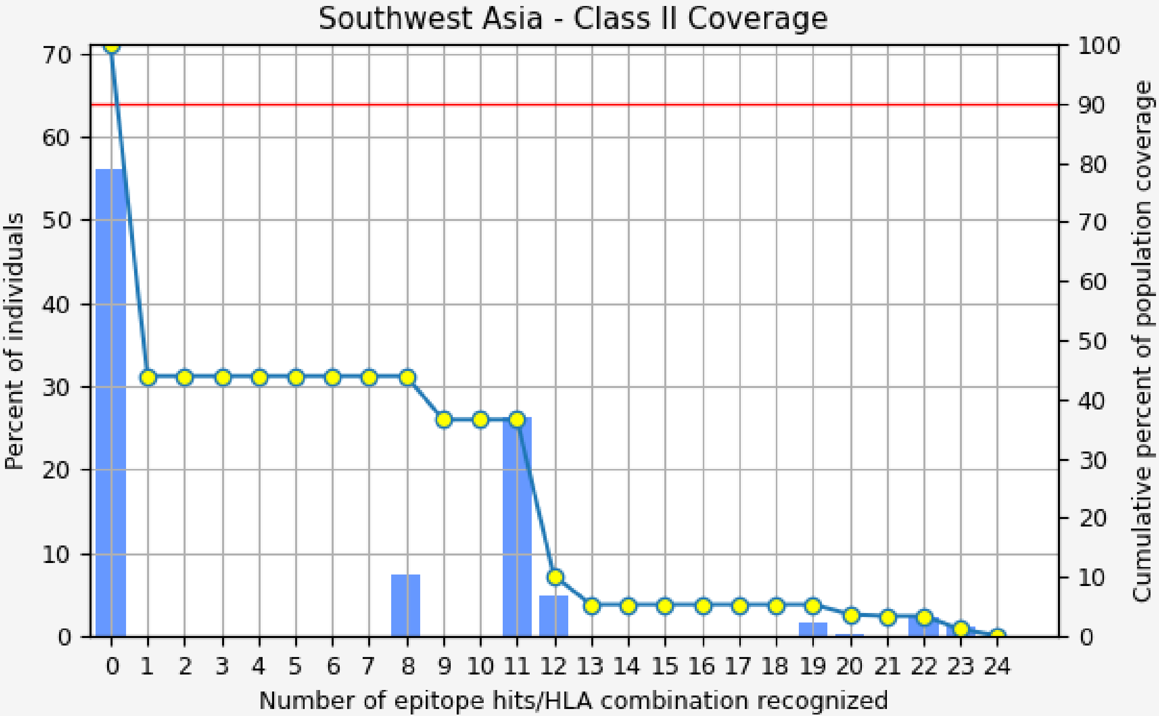
Southwest Asia - Class II Coverage
Percent of individuals
Cumulative percent of population coverage
Number of epitope hits/HLA combination recognized
0 10 20 30 40 50 60 70
0 10 20 30 40 50 60 70 80 90 100
0 1 2 3 4 5 6 7 8 9 10 11 12 13 14 15 16 17 18 19 20 21 22 23 24

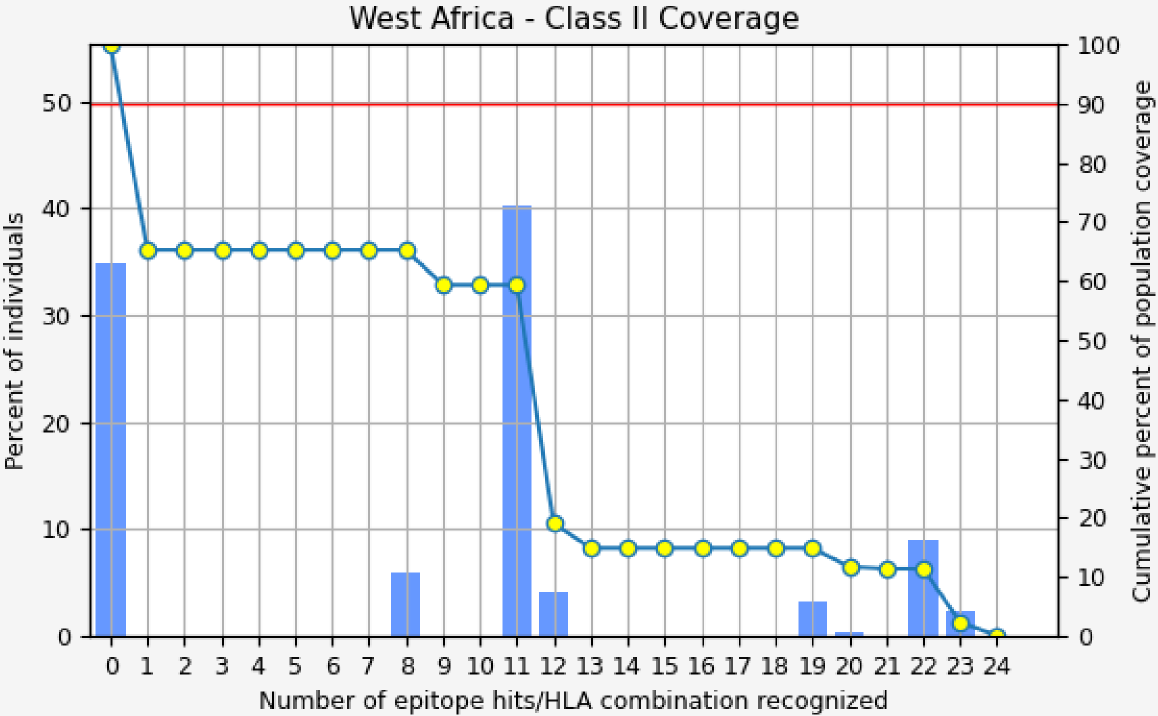

West Africa - Class II Coverage
Percent of individuals
Cumulative percent of population coverage
Number of epitope hits/HLA combination recognized
0
10
20
30
40
50
0 1 2 3 4 5 6 7 8 9 10 11 12 13 14 15 16 17 18 19 20 21 22 23 24
0 10 20 30 40 50 60 70 80 90 100

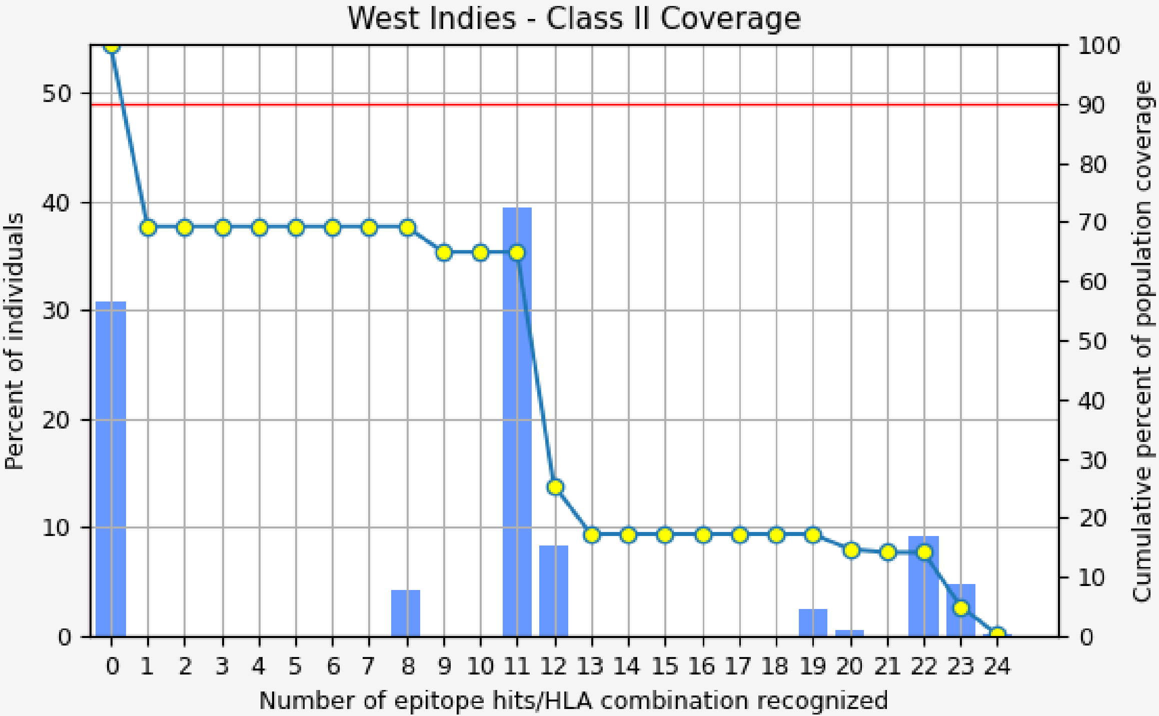
West Indies - Class II Coverage
Percent of individuals
Cumulative percent of population coverage
Number of epitope hits/HLA combination recognized
0
10
20
30
40
50
0
10
20
30
40
50
60
70
80
90
100
0 1 2 3 4 5 6 7 8 9 10 11 12 13 14 15 16 17 18 19 20 21 22 23 24
